# Oncogenic lncRNAs alter epigenetic memory at a fragile chromosomal site in human cancer cells

**DOI:** 10.1126/sciadv.abl5621

**Published:** 2022-03-02

**Authors:** Ganesan Arunkumar, Songjoon Baek, David Sturgill, Minh Bui, Yamini Dalal

**Affiliations:** Laboratory of Receptor Biology and Gene Expression, Center for Cancer Research, National Cancer Institute, NIH, Bethesda, MD 20892, USA.

## Abstract

Chromosome instability is a critical event in cancer progression. Histone H3 variant CENP-A plays a fundamental role in defining centromere identity, structure, and function but is innately overexpressed in several types of solid cancers. In the cancer background, excess CENP-A is deposited ectopically on chromosome arms, including 8q24/*cMYC* locus, by invading transcription-coupled H3.3 chaperone pathways. Up-regulation of lncRNAs in many cancers correlates with poor prognosis and recurrence in patients. We report that transcription of 8q24-derived oncogenic lncRNAs plays an unanticipated role in altering the 8q24 chromatin landscape by H3.3 chaperone–mediated deposition of CENP-A–associated complexes. Furthermore, a transgene cassette carrying specific 8q24-derived lncRNA integrated into a naïve chromosome locus recruits CENP-A to the new location in a cis-acting manner. These data provide a plausible mechanistic link between locus-specific oncogenic lncRNAs, aberrant local chromatin structure, and the generation of new epigenetic memory at a fragile site in human cancer cells.

## Teaser

Oncogenic transcripts drive histone variant misincorporation at fragile sites and induce aberrant chromatin structure.

## INTRODUCTION

Genomic instability, including chromosome breaks and translocations, is a hallmark of cancer cells, leading to tumor initiation and progression (*1*). Several key pathways contribute to this phenomenon (*2*). One such example is the misregulation of histones, which alters both global gene expression and physical-mechanical properties of chromosomes (*3*–*6*). Histones are conserved DNA packaging proteins in eukaryotic cells, expressed in nonallelic variant forms (*7*, *8*). Histone H3, one of the core histones, has four variants, of which centromeric protein-A (CENP-A) is essential for mitosis, and generally only enriched in centromere-specific nucleosomes (*9*), which in humans is composed of repetitive alpha-satellite elements (*10*, *11*). At this locus, CENP-A partners with key inner kinetochore proteins, such as CENP-C and CENP-N (*12*). In humans, CENP-A is deposited at the centromeres during the early G_1_ phase by its specific chaperone HJURP (*13*–*16*). Later work demonstrated that not only perturbations in the interaction between CENP-A and its chaperone HJURP (*17*) but also an abrogation of centromeric transcription (*18*–*25*) results in inefficient loading of CENP-A and weakening of the centromeres, resulting in downstream accumulation of mitotic defects (*26*).

Despite strict regulation of CENP-A in normal cells, several groups, including our own, have reported that in diverse human cancer cells and tumors, CENP-A is innately overexpressed and mislocalized to regions outside centromeres, typically to chromosome arms and telomeres (*27*–*34*). Overexpression of CENP-A is associated with poor clinical prognosis and currently serves as a predictive biomarker in cancer detection panels (*27*, *30*, *35*). In native and artificial CENP-A overexpression conditions, the mechanistic basis for CENP-A’s correlation with disease severity has been linked to the accumulation of this protein at ectopic loci, which sensitizes them to damage (*30*, *36*). In this context, several fundamental questions remain to be elucidated at the mechanistic level. The basis for deposition of CENP-A at specific noncentromeric genomic regions, the basis for epigenetic memory of such sites over extended periods of time, the potential conversion of such ectopic locations, some of which are fragile sites, into neocentromeres, and the relationship to DNA damage are yet to be fully understood.

In this report, we focus on these fundamental questions in the context of the subtelomeric chromosome locus 8q24. Ever since the landmark discovery of amplification and translocation of the 8q24 locus (which contains the *cMYC* gene) in lymphomas nearly 40 years ago, the fragility, amplification, and translocation of 8q24/*cMYC* have been documented in numerous cancers (*37*–*39*). We previously reported that 8q24 has a large CENP-A domain in patient-derived colon cancer cells, extending to several types of solid tumors but not in normal cells (*27*, *29*). Furthermore, a large DNA hypersensitive site over 8q24 is reliably correlated with ectopic CENP-A at this location (*27*). Deoxyribonuclease I (DNase I) hypersensitivity is associated with both, a more accessible chromatin state, and with active transcription (*40*, *41*). However, 8q24 is a gene desert, devoid of any protein-coding genes except *cMYC* (*42*). It does encode five unique sequence long noncoding RNAs (lncRNAs), namely, *PCAT1*, *PCAT2*, *CCAT1*, *CCAT2*, and *PVT1*. These oncogenic lncRNAs are clinically associated with cell proliferation, metastasis, and therapeutic resistance to treatment (*42*–*46*). Therefore, 8q24 serves as an excellent proxy to probe the epigenetic relationship between the transcriptional activity of oncogenic lncRNAs arising from a specific locus and the aberrant incorporation of a key histone variant at that locus.

We find that transcription of oncogenic lncRNAs from the native 8q24 locus enables robust recruitment of the histone variant CENP-A in colon cancer cells. Targeted destruction of these RNAs results in marked loss of CENP-A occupancy at the 8q24 locus. Furthermore, we report that CENP-A occupancy relies on cooperation between transcriptionally coupled H3.3 chaperones and lncRNAs, with a possible correlation to R-loop formation at the target locus. Last, we demonstrate that one of these lncRNAs, translocated to a naïve locus on another chromosome, is sufficient to drive CENP-A to that locus in a cis-acting manner. Together, these data suggest an oncogenic lncRNA-based mechanism by which aberrant epigenetic signatures can be generated. These data have implications for neocentromere formation, chromosome breaks, and amplification in the cancer epigenome.

## RESULTS

### Stability of ectopic CENP-A domains in human cancer cells

**Fig. 1.**
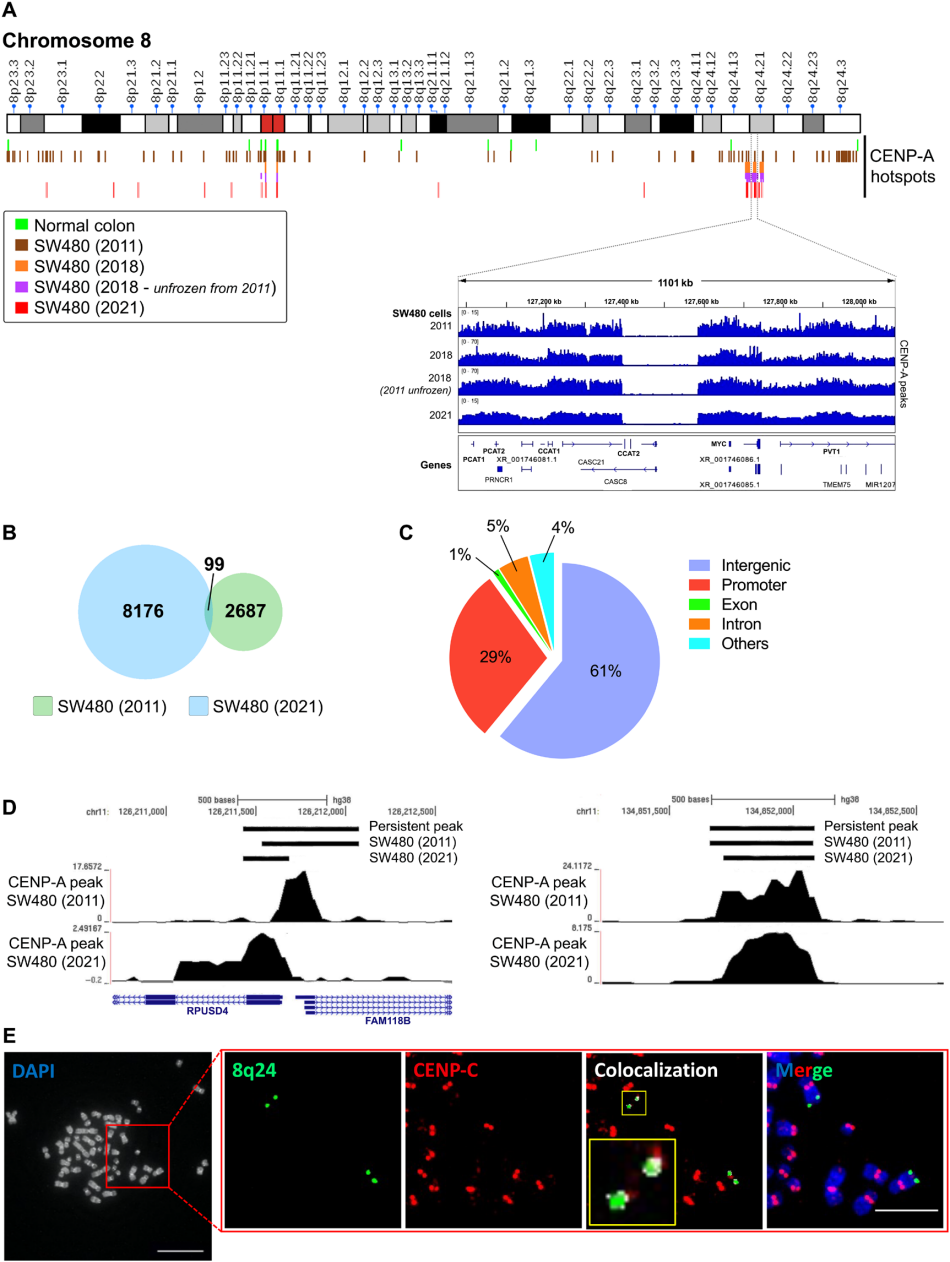
Chromosome 8q24/*cMYC* locus is a stable CENP-A ectopic site for CENP-A. (**A**) Graphical illustration of CENP-A peaks on chromosome 8 from normal colon cells (huEpiCol) and SW480 colon cancer cells. Representative CENP-A ChIP-seq peaks at the 8q24/*cMYC* locus from sample SW480 colon cancer cell line performed in the top to bottom order in 2011, 2018, 2011 (unfrozen samples performed along with the 2018 sample), and 2021. CENP-A signature at the 8q24 locus remains consistently robust for a decade in SW480 colon cancer cells, as shown in the ChIP-seq peaks in inset image. (**B**) Venn diagram showing the number of total CENP-A peaks and common peaks present in SW480 colon cancer cells that were sequenced in 2011 and 2021. (**C**) Pie chart showing the distribution of persistent ectopic CENP-A peaks in genic and intergenic regions of the SW480 colon cancer cells. (**D**) Representative persistent CENP-A ChIP-seq peaks at a gene promoter region (left) and an intergenic region (right) in chromosome 11 of SW480 colon cancer cells. (**E**) The metaphase chromosomes of SW480 colon cancer cell were IF-labeled for CENP-C (red) and the 8q24 locus by DNA-FISH (green). The inset images show the colocalization signal (white in the yellow inset) of CENP-C at the 8q24 region. The CENP-C channel (red) in the inset figure is enhanced to visualize the signal from ectopic sites. Scale bars, 10 μm (main merged image) and 1 μm (inset merged image).

We first investigated the stability of the ectopic CENP-A domains identified by genome-wide high-throughput sequencing of colon cancer cells continuously maintained over a decade in our laboratory (Fig. 1A). We find that persistent ectopic CENP-A sites are enriched in intergenic and gene promoter regions (Fig. 1, B and C). Notably, this ectopic CENP-A domain at the 8q24 locus remains stable over 10 years, even after the locus has undergone amplification and translocation (*27*, *29*). Furthermore, this locus also colocalizes with essential inner and outer kinetochore complex proteins such as CENP-C and NDC80, respectively (*47*). NDC80 bridges centromere attachment to microtubules (*47*), which suggests that, in these cells, 8q24 has the potential to act as a weak neocentromere (*29*). Notably, native human centromeres are composed of high-order repeats of alpha-satellite DNA enriched with centromere-specific CENP-B protein binding CENP-B box and transposable repeat elements (*48*). Therefore, we first systemically examined a possible genetic basis for CENP-A retention at stable ectopic CENP-A domains that naturally occur in these colon cancer cells (table S1) (*27*).

Our bioinformatic screening identified no notable enrichment of most long interspersed nuclear elements (LINEs) and long terminal repeats (LTRs) at ectopic CENP-A domains compared to non–CENP-A regions (figs. S1 and S2). However, one LTR element (MLT1C) showed a modest 1.66-fold enrichment but which was unequally represented across individual ectopic CENP-A domains and is also slightly enriched in the rest of the genome. Notably, retroviral elements present within centromeres affect CENP-A levels (*18*), which suggests that this could also be feasible at ectopic locations. Thus, such repetitive elements or their expression may serve as local drivers for ectopic CENP-A loading. Repeat elements present near transcribing promoters of genes present in the CENP-A domains, which could alter the gene expression pattern (*49*), showed reduced enrichment relative to promoters as a whole (fig. S3, A and B). We also performed motif analysis for the inner kinetochore protein CENP-B, which specifically binds the CENP-B box sequence. However, we did not detect CENP-B box–like sequences enriched within ectopic CENP-A domains (table S2). To be certain of this interpretation, we also performed CENP-B immunofluorescence (IF)–DNA–fluorescence in situ hybridization (FISH) specifically at the 8q24 locus. In agreement with our bioinformatic analysis, we were unable to detect CENP-B visually at this locus (fig. S3C). Sequence consensus analysis and *k*-mer analysis identified that all five 8q24-derived lncRNAs are unique in their sequences and sequence compositions distinct from centromeric repeats and putative centromeric transcripts (tables S2 and S3 and figs. S4 and S5) (*50*). Thus, these bioinformatic analyses suggest that other epigenetic factors may be responsible for recruiting or retaining CENP-A at certain ectopic sites.

We next turned our attention to epigenetic features that may underlie the stability of specific noncentromeric CENP-A domains. In several species, it is now well established that the renewal of CENP-A epigenetic memory at centromeres in every cell cycle, in part, relies on transcription of, and noncoding transcripts arising from, repetitive alpha-satellite centromeric DNA (*21*, *51*–*54*). We were curious whether the stable CENP-A signature we detected by chromatin immunoprecipitation sequencing (ChIP-seq) and IF/FISH at the 8q24 locus could also be linked to well-documented hyperactive transcription of 8q24-specific noncoding genes in several types of cancer cells, including colon tumors (*42*). We hypothesized that noncoding transcripts emanating from the 8q24 locus may act as an epigenetic signal for CENP-A recruitment.

### Targeted depletion of lncRNAs from 8q24 depletes ectopic CENP-A and CENP-C

To test this hypothesis, we first confirmed that these lncRNAs are expressed in our SW480 metastatic colon cancer cells, which has five copies of the 8q24 locus (fig. S6). In order of magnitude, *CCAT1* is the most highly expressed, followed by *PCAT2*, *PCAT1*, *PVT1*, and, finally, *CCAT2* (fig. S7A). Next, we performed antisense oligo (ASO)–mediated knockdown versus scrambled control of the five 8q24-derived lncRNAs individually for 72 hours in our colon cancer cell lines (fig. S7B). As reported earlier (*27*, *29*), we detect that, on average, two of the five copies of the 8q24 locus have CENP-A/CENP-C on them. Therefore, we pooled readings from innate and translocated 8q24 loci for the knockdown experiments. Using methanol-fixed or unfixed chromosome metaphase spreads, and either CENP-A or inner kinetochore protein CENP-C, we examined the consequences of the loss of these lncRNAs on ectopic CENP-A (fig. S8A). CENP-C directly associates with CENP-A nucleosomes, as a proxy for CENP-A staining. Several studies, including our own, have shown that CENP-C localizes to ectopic sites along with CENP-A in cancer cells (Fig. 1E) (*29*, *31*, *55*) and also that ectopic CENP-A partners with histone H3.3 to form hybrid nucleosomes, which have unusual properties (*3*, *27*, *56*, *57*).

**Fig. 2.**
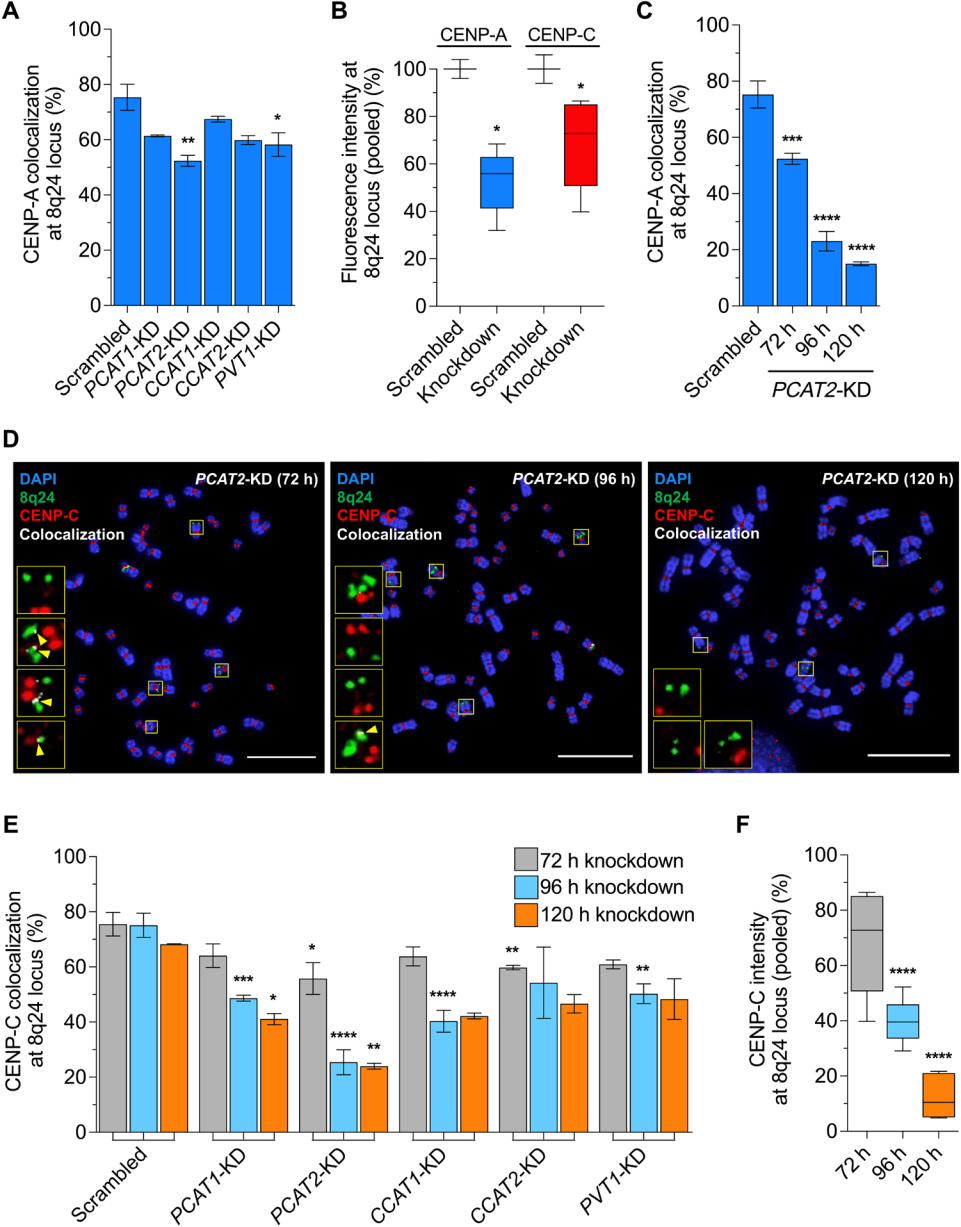
Knockdown of the 8q24-derived lncRNAs depletes mislocalized CENP-A and CENP-C levels. (**A**) Percentage colocalization foci of CENP-A at the 8q24 locus after 72-hour knockdown of the 8q24-derived lncRNAs in SW480 colon cancer cells compared to scrambled control. (**B**) Pooled levels of CENP-A and CENP-C at the 8q24 locus after 72-hour knockdown of the 8q24-derived lncRNAs in SW480 colon cancer cells. (**C**) Percentage colocalization foci of CENP-A at the 8q24 locus after 72- to 120-hour knockdown of lncRNA *PCAT2*, as a representative, which showed significant effect on ectopic CENP-A compared to other 8q24 locus–derived lncRNAs. (**D**) Representative metaphase chromosome IF-DNA-FISH images of SW480 cells treated with ASO against *PCAT2* for 72, 96, and 120 hours. CENP-C (red) colocalization (white) at the 8q24 locus (green) decreased significantly upon *PCAT2* knockdown. Yellow arrows point the colocalization spots. Scale bars, 10 μm. (**E**) Percentage CENP-C colocalization foci at the 8q24 locus after knockdown of the 8q24-derived lncRNAs at 72, 96, and 120 hours. (**F**) Histogram showing the averaged levels of CENP-C signal intensity at the 8q24 locus after knockdown of all five 8q24-derived lncRNAs (averaged intensity readings from individual knockdowns are pooled). The levels of CENP-C were significantly reduced on increasing knockdown treatment time point. All data are shown as means ± SD. *P < 0.05, **P < 0.01, ***P < 0.001, and ****P < 0.0001.

As measured by IF/FISH colocalization, the knockdown of these five lncRNAs, relative to scrambled controls, depleted ectopic CENP-A and CENP-C at the 8q24 locus (Fig. 2, A and B). We next extended the knockdown of 8q24-derived lncRNAs from 96 to 120 hours (fig. S7B). At 120 hours after depletion, CENP-A and CENP-C loss is most pronounced in the *PCAT2* knockdown background, going down to 15 and 23% occupancy at the 8q24 locus relative to scrambled control, respectively (Fig. 2, C to E, and fig. S8, B and C). We also observed a more pronounced reduction of CENP-C at the 8q24 locus over increasing time points in all the lncRNA knockdowns (Fig. 2F). We next performed combinatorial lncRNA double knockdowns for 72 hours. These elicited a similar trend of CENP-C reduction at 8q24, but at a faster rate (fig. S8D). As a control, we also assessed the centromeric and soluble CENP-A, CENP-C, or H3.3 levels by IF and immunoblotting. Levels of these proteins remained unaltered in all the knockdown experiments (figs. S9 and S10). Notably, ASO-mediated lncRNA *PCAT2* knockdown has the greatest impact on CENP-A/CENP-C levels at the 8q24 locus, reducing CENP-C levels down to 38% of wild-type levels (fig. S8C).

### Loss of 8q24-derived lncRNAs affects ectopic CENP-C levels locally, not globally

**Fig. 3.**
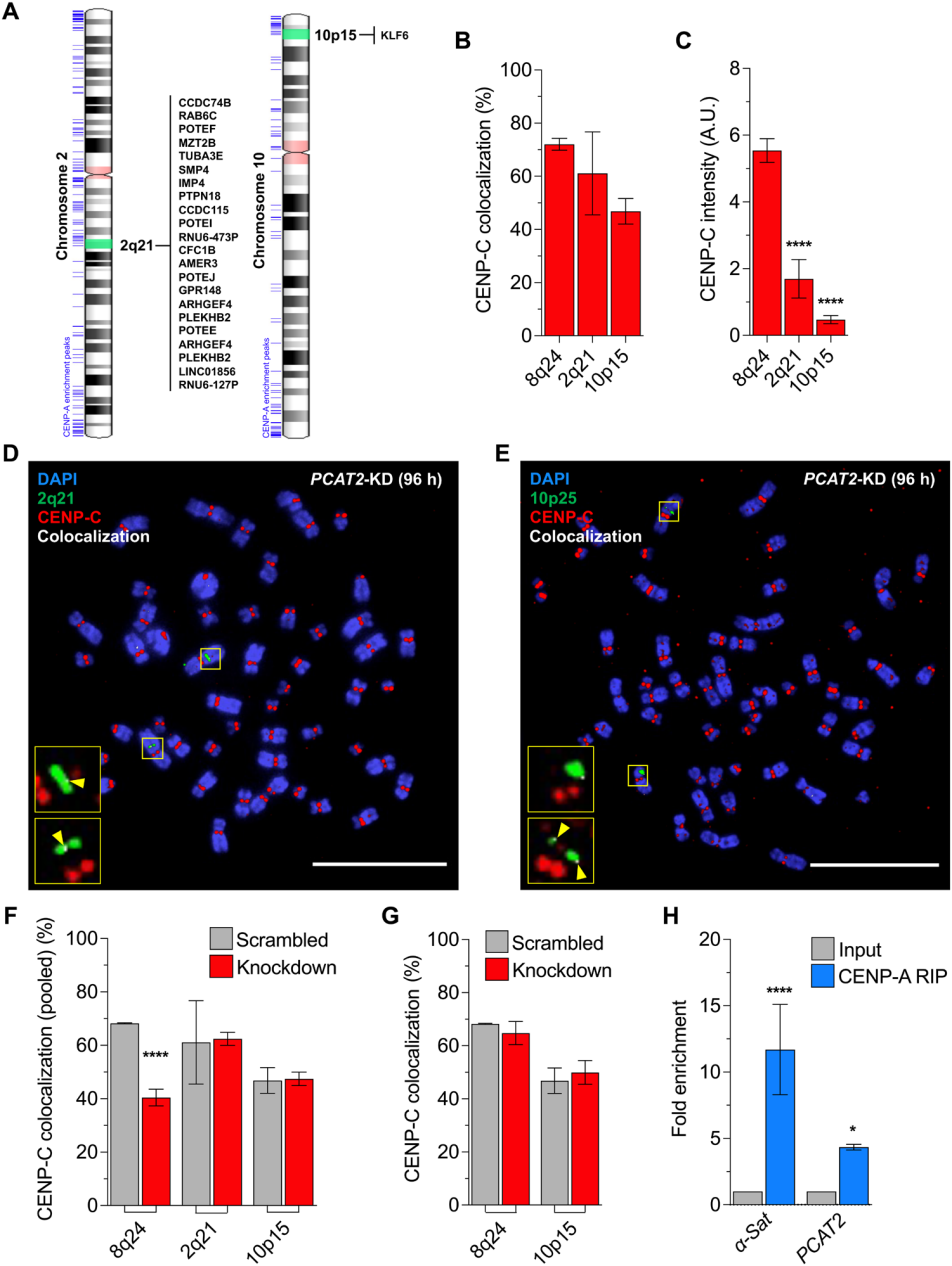
8q24-derived lncRNAs acts in cis and *PCAT2* associates with CENP-A. (**A**) Ideogram of chromosome 2 (left) and chromosome 10 (right) with the list of genes present at the selected DNA-FISH probe (2q21 and 10p15, green shaded) location that served as a control locus. CENP-A ChIP-seq enrichment peaks were shown next to the ideogram (left, blue lines). (**B**) Histogram showing the percentage CENP-C colocalization foci at the 8q24, 2q21, and 10p15 loci in SW480 colon cancer cells. (**C**) Fluorescence intensity of CENP-C scored on metaphase chromosomes at the 8q24, 2q21, and 10p15 loci from fixed SW480 colon cancer cells. The 8q24 locus has higher levels of ectopic CENP-A compared to the other two loci. The signal intensities are normalized against the background. A.U., arbitrary units. (**D** and **E**) Representative metaphase chromosome IF-DNA-FISH images of SW480 cells treated with ASO against *PCAT2*. CENP-C (red) colocalization (white) levels are unaltered at the 2q21 (D) and 10p15 (E) loci (green) after 96-hour knockdown of *PCAT2*. Yellow arrows point the colocalization spots. Scale bars, 10 μm. (**F**) Percentage CENP-C colocalization foci at the 8q24, 2q21, and 10p15 loci after knockdown of 8q24-derived lncRNAs for 96 hours (data pooled from all lncRNA knockdowns). (**G**) Percentage CENP-C colocalization foci at the 8q24 and 10p15 loci in SW480 cells after 72-hour *KLF6* knockdown. (**H**) qPCR result showing that the fold enrichment of alpha-satellite (α-Sat) and *PCAT2* RNAs in CENP-A-RIP samples compared to input (*P* = 0.0001 and 0.0347, respectively) lncRNA *PCAT2* is the only 8q24-derived lncRNA to be detected in the qPCR among the others. All data are shown as means ± SD. *P < 0.05 and ****P < 0.0001.

LncRNAs can act in cis and trans fashion to enact their functions. LncRNAs enriched at their sites of transcriptional origin can participate in the modulation of chromatin structure, chromatin modifications, and transcription control. For example, the *XIST* RNA, which transcribes from the inactive X-chromosome, acts in cis through chromatin binding (*58*). In contrast, *Pnky*, a regulator of cortical development, is a trans-acting lncRNA (*59*). To determine whether 8q24-derived lncRNAs help recruit CENP-A through cis- or trans-acting mechanisms, we compared the 8q24 locus with two other known persistent ectopic CENP-A sites—chromosome 2 q-arm (2q21.1) and chromosome 10 p-arm (10p15.2) (Fig. 3A and fig. S11). The 2q21 locus has several coding genes and only one lncRNA gene, in contrast to the 8q24 locus gene content. On the other hand, 10p15 is a gene desert that resembles the 8q24 locus spanning only one coding gene, *KLF6*, but has no other annotated lncRNA genes. Studying these sites allowed us to test whether transcription of coding genes also favors ectopic CENP-A deposition.

IF-DNA-FISH analysis showed that 76% of the counted 8q24 loci colocalize with CENP-C, but only 63 and 45% colocalize with 2q21 and 10p15 locus, respectively (Fig. 3B). Both 2q21 and 10p15 loci had low levels of CENP-C intensity signal compared to the 8q24 locus (Fig. 3C), i.e., the 8q24 locus has more molecules of CENP-C protein than the other sites. Knockdown of 8q24-derived lncRNAs for 96 hours depleted the CENP-C only at the 8q24 locus but not at the other two sites (Fig. 3, D to F). Alternatively, when we knockdown *KLF6* from the 10p15 locus, we did not observe a significant change in CENP-C levels at either 10p15 or 8q24 locus (Fig. 3G and fig. S12). *KLF6* is a tumor suppressor and a transcription factor, also identified as a prognostic marker, involved in many cellular processes (*60*). Knockdown of the tumor-suppressive coding gene *KLF6* did not affect CENP-C levels, suggesting that mRNAs do not recruit CENP-A. It is plausible that unannotated/cryptic transcripts near coding loci might assist CENP-A mislocalization (*61*).

Overall, our results suggest that specific noncoding transcripts emanating from the 8q24 locus contribute significantly to CENP-A mislocalization. Furthermore, the depletion of CENP-C only at the 8q24 locus in the lncRNA depletions suggests that the oncogenic lncRNAs function in cis to recruit CENP-A to the locus from which they transcribe. This observation made us curious to probe whether CENP-A is physically associated with these particular lncRNAs.

### The 8q24 lncRNA *PCAT2* is physically associated with CENP-A/CENP-C and H3 in vivo but not in vitro

The physical association of centromeric lncRNAs with CENP-A and its centromeric chaperone HJURP appears to be involved in CENP-A deposition at centromeres (*21*). However, whether CENP-A can bind noncentromeric lncRNAs involved in its ectopic localization in cancer cells has not been investigated. To probe this question further, we performed RNA immunoprecipitation (RIP) from our colon cancer cells using an anti–CENP-A antibody. The immunoprecipitated RNA samples were treated with DNase I to ensure that the samples are free from genomic DNA contamination. We performed quantitative polymerase chain reaction (qPCR) and semiquantitative PCR to test for the presence of five 8q24-derived lncRNAs, as well as coding mRNAs *cMYC* and *KLF6*, along with centromeric alpha-satellite RNA as a positive control and *18S rRNA* as a negative control. As detected by a probe against the consensus sequence for alpha-satellite (*21*), we confirm its expected physical association with CENP-A in these colon cancer cells (Fig. 3H). Our CENP-A RIP-qPCR results showed that *PCAT2* was the only 8q24-derived lncRNA that shows a strong positive amplification signal, suggesting direct interaction. However, the other four 8q24-derived lncRNAs could not be detected (Fig. 3H), presumably because they do not interact with CENP-A strongly enough to be captured by the current protocol. We also confirmed the CENP-A RIP *PCAT2* amplification using a semiquantitative PCR analysis (fig. S13, A and B). Neither of the mRNA species *cMYC* nor *KLF6* mRNAs were enriched in CENP-A RIP samples (fig. S13A).

As discussed above, ectopic CENP-A partners with H3 to make hybrid particles and recruits CENP-C to ectopic sites such as 8q24. Therefore, we also tested CENP-C and H3 RIP for *PCAT2* enrichment. We found that *PCAT2* also copurifies with CENP-C and H3 (fig. S13, C and D). In addition, CENP-C also binds to *cMYC* mRNA (fig. S13C). When we performed RIP-qPCR using immunoglobulin G as a mock, we did not detect *PCAT2* amplification, suggesting that *PCAT2* binding to proteins is not nonspecific, but there is some specificity toward chromatin-associated complexes. Furthermore, as we observed above, ectopic CENP-A nucleosomes copurify with H3.3 in cancer cells (*3*, *27*, *56*, *57*). Thus, at the 8q24 locus, *PCAT2* could be bound to both CENP-A and H3 and also to the CENP-A binding protein CENP-C, along the chromatin fiber. An alternative possibility is that *PCAT2* RNA is opportunistic and binds nonspecifically to the most prevalent histone complex in its vicinity. We were curious to test whether in the absence of any chaperones, in vitro transcribed *PCAT2* RNA could interact directly with CENP-A histones (dimers, tetramers, or nucleosomes). These experiments did not yield robust binding (note S1). These data, in addition to the RIP data above, suggested to us that the nucleoprotein association is unlikely to be in the soluble form, but perhaps depends on the chromatin context, or on processes that can bridge the RNA to the histone. This lack of binding in the in vitro assay suggests that *PCAT2* binding to CENP-A and H3 is not only charge or mass action based (note S1 and fig. S21, D to G) but also specific to the context of the chromatin fiber in vivo.

### LncRNA *PCAT2* cooperates with H3.3 chaperones to promote CENP-A mislocalization

We and others have previously reported that excess CENP-A either in human cancer cells (*3*, *29*) or when overexpressed in other species (*62*, *63*) depends on transcriptionally coupled H3.3 chaperones for its deposition at high nucleosome turnover ectopic sites. Therefore, the next logical possibility was that lncRNA *PCAT2* interacts with CENP-A via transcriptionally coupled H3.3 chaperones, namely, HIRA and DAXX (*29*).

**Fig. 4.**
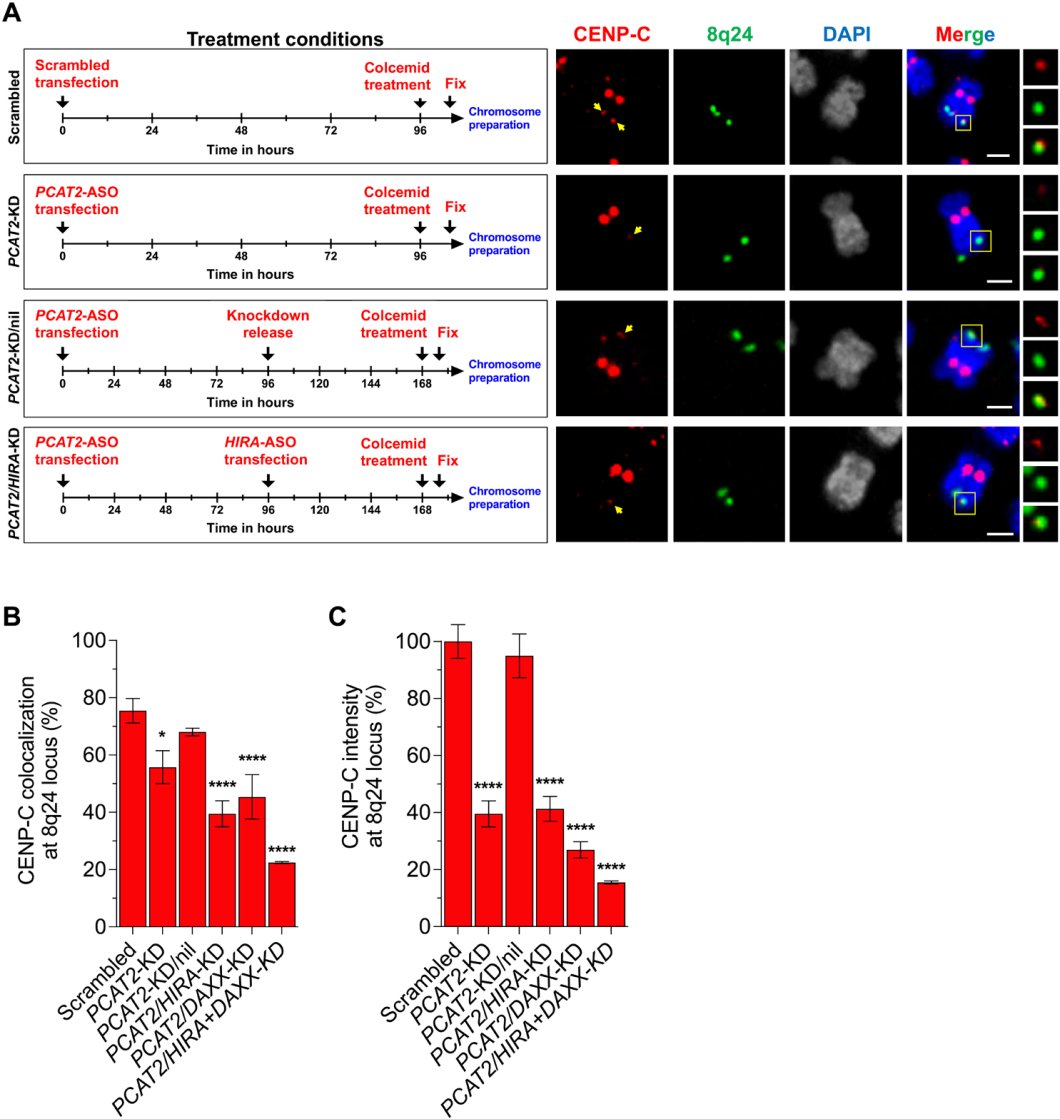
LncRNA *PCAT2* cooperates with H3.3 chaperones for CENP-A ectopic deposition. (**A**) Schematics of treatment conditions, timeline (left), and representative IF-DNA-FISH image (right) of chromosome 8 displaying the change in CENP-C levels (red) at the 8q24 locus (green) when lncRNA *PCAT2* and H3.3 chaperones, HIRA, and DAXX were knocked down sequentially. In these knockdown experiments, *PCAT2* was depleted for 96 hours. The cells were then either harvested, allowed to proliferate in fresh medium, or knocked down by H3.3 chaperones for another 96 hours based on the experimental need as shown in the schematics. The chromosome 8 images in the left, innate copy of chromosome 8 stained for q24 band using DNA-FISH and CENP-C by IF, are representative of the knockdown experiments. Scale bars, 1 μm. (**B**) Percentage CENP-C colocalization foci at the 8q24 locus in the scrambled, *PCAT2*-KD, *PCAT2*-nil, and *PCAT2*/H3.3 chaperone knockdown conditions. (**C**) Fluorescence intensity of CENP-C at the 8q24 locus in the scrambled, *PCAT2*-KD, *PCAT2*-nil, and *PCAT2*/H3.3 chaperone knockdown conditions. LncRNA *PCAT2* knockdown depleted ectopic CENP-A from the 8q24 locus. However, releasing the cells from knockdown allowed the locus to gain ectopic CENP-A to 8q24, comparative to the scrambled control levels. Sequential knockdown of H3.3 chaperones followed by the lncRNA *PCAT2* prevented CENP-A from loading at the 8q24 locus. All data are shown as means ± SD. *P < 0.05 and ****P < 0.0001.

To test this idea, we designed a genetic experiment in vivo, depleting lncRNA *PCAT2* and H3.3 chaperones sequentially, while examining for CENP-C occupancy at 8q24 (Fig. 4A). First, we treated the cells with scrambled and ASO against *PCAT2* individually for 96 hours. Second, we knocked down *PCAT2* for 96 hours and released the knockdown treatment by growing the cells in a fresh medium without ASO for another 72 hours. Last, two sets of cells were treated with ASO against lncRNA *PCAT2* for 96 hours followed by either *HIRA* or *DAXX* ASOs for 72 hours (fig. S14A). Cells were arrested at metaphase in all the conditions and scored for the CENP-C levels at the 8q24 locus using IF-DNA-FISH.

As observed earlier (Fig. 2B), *PCAT2* knockdown depleted CENP-C from the 8q24 locus. However, the cells regained CENP-C at the 8q24 locus after 72 hours when released from the knockdown (Fig. 4B). The sequential knockdown of *HIRA* or *DAXX* and *HIRA-DAXX* followed by *PCAT2* prevented recruitment of CENP-C to the 8q24 locus (Fig. 4, A to C). Notably, the CENP-C level at the 8q24 locus in the latter condition was further reduced than that observed with just the *PCAT2* knockdown (Fig. 4, B and C). As a negative control, we also assessed centromeric CENP-C levels at the native chromosome 8 centromere; these remained unaltered under these experimental conditions (fig. S14B). These data suggest that CENP-C/A at ectopic sites is not detectably titrated away from native centromeres but most likely recruited from the soluble pool. Notably, knockdown treatment of *PCAT2* followed by combinatorial knockdown of *HIRA* and *DAXX* resulted in chromosome deformities, and a significant proportion of cells are nonviable (fig. S14C).

Together, these results demonstrate that the lncRNA *PCAT2* cooperates with H3.3 chaperones for CENP-A mislocalization in cancer cells. Further, even after the release of lncRNA *PCAT2* from the knockdown, CENP-C levels do not increase at the 8q24 locus without HIRA or DAXX. These data support previous work demonstrating that these chaperones are essential for ectopic CENP-A loading (*3*, *29*). We interpret these data to mean that, in the absence of H3.3 chaperones, *PCAT2* RNA alone cannot mechanize CENP-A ectopic deposition. We also tested whether transcription-mediated chromatin structure could be involved in CENP-A deposition, and found modest evidence in support of R-loops being involved in ectopic mislocalization (note S2 and figs. S22 to S24).

### Translocated 8q24 locus recruits CENP-C at a higher level than native chromosome 8

Chromosome rearrangements result in the formation of cryptic promoters, loss of enhancers, and altered cis-regulatory elements that affect gene expression (*64*). Notably, in addition to the two innate copies of the 8q24 locus, the SW480 colon cancer cells have three translocated copies of this locus (figs. S6 and S15A). The innate 8q24 locus exhibits a compact chromatin structure, like that of the 2p21 and 10p15 loci, which are not translocated. In contrast, the translocated 8q24 locus has a diffused DNA-FISH signal implying an open chromatin (fig. S15, A and B). Therefore, we hypothesized that the translocated 8q24 locus could involve higher transcriptional activity due to gain or loss of cis-regulatory elements and favor more CENP-A deposition.

To test this idea, we examined the CENP-C levels between innate and translocated 8q24 locus and observed a significantly higher level of CENP-C at the translocated 8q24 locus (fig. S15, C and D). This observation emphasizes that the translocated 8q24 locus might have lost the spatiotemporal regulation operating at the native site, resulting in unregulated transcriptional activity, thus increasing the chance of CENP-A mislocalization at the translocated locus. Moreover, considering the role of cis-regulation in CENP-A mislocalization, it is possible that introducing genes from the 8q24 locus to a naïve chromosome locus could result in the formation of a novel CENP-A domain.

### Transgene *PCAT2* can mislocalize CENP-C at a naïve locus in a cis-acting manner

**Fig. 5.**
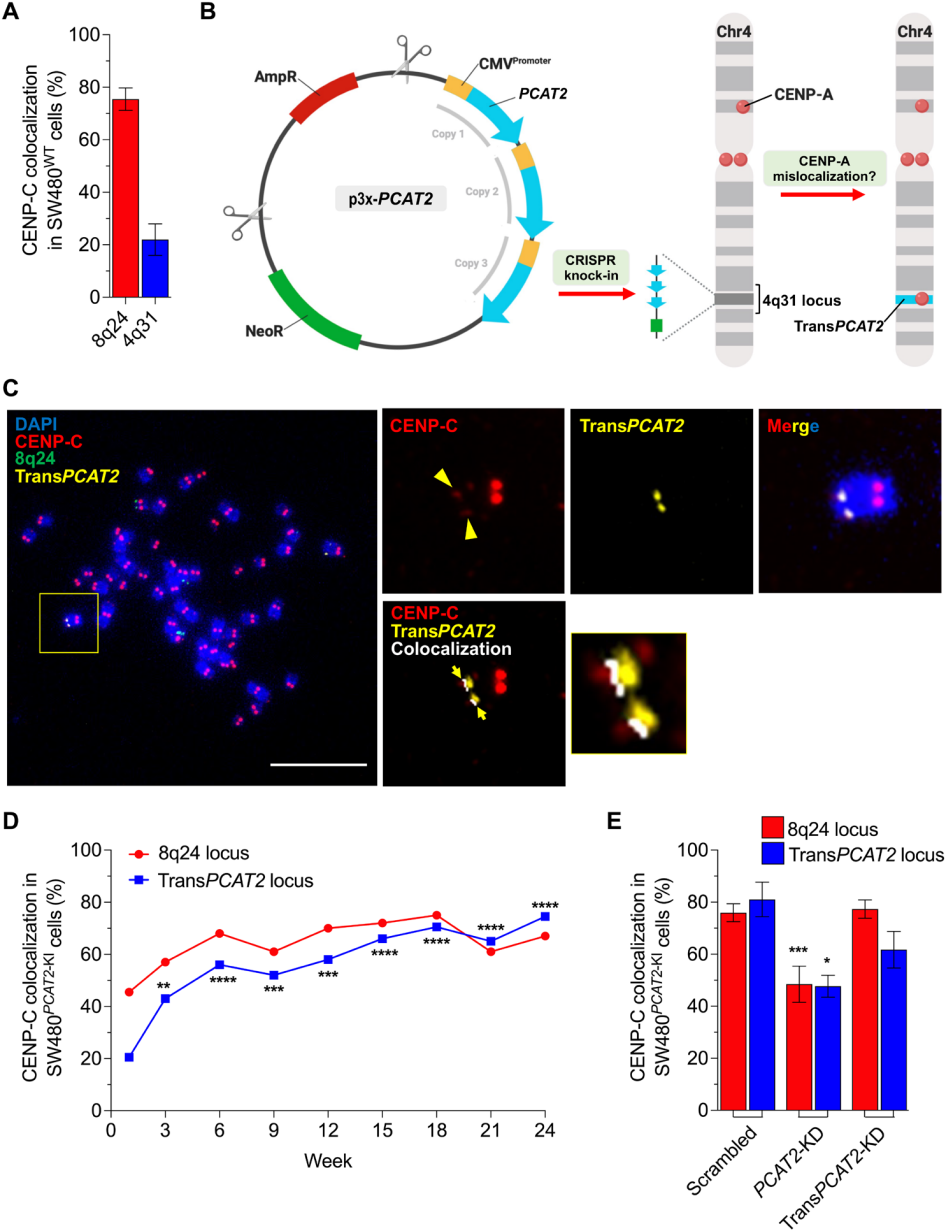
Transgene *PCAT2* altered the epigenetic signature of a naïve chromosome locus. (**A**) Percentage CENP-C colocalization foci at the 8q24 and 4q31 loci of SW480^WT^ cells before knock-in of Trans*PCAT2* gene array. (**B**) Schematic map of p3x-*PCAT2* plasmid and CRISPR knock-in of the transgene *PCAT2* into the chromosome 4q31 locus to study the mislocalization of CENP-A to the new locus (4q31 locus in cyan). (**C**) IF-DNA-FISH image of metaphase chromosomes of SW480^*PCAT2*-KI^ colon cancer cell displaying the 8q24 locus (green) and transgene *PCAT2* locus (Trans*PCAT2*) investigated using the 4q31 locus FISH probe (yellow). The SW480^*PCAT2*-KI^ cells acquired significant level of CENP-C at the Trans*PCAT2* insert site (4q31 locus), and colocalization of CENP-C at the locus is shown in white (inset, right bottom). Scale bars, 10 μm. (**D**) Percentage CENP-C colocalization foci at the 8q24 and Trans*PCAT2* loci of SW480^*PCAT2*-KI^ colon cancer cells at weeks 1, 3, 6, 9, 12, 15, 18, 21, and 24 after CRISPR knock-in and selection. SW480^*PCAT2*-KI^ cells colocalized a significant level of CENP-C on week 24 at the Trans*PCAT2* locus, outcompeting the levels at the native 8q24 locus. (**E**) Percentage CENP-C colocalization foci at the 8q24 and Trans*PCAT2* loci after knocking down innate *PCAT2* and Trans*PCAT2* RNAs (expressing from the Trans*PCAT2* locus) compared to the scrambled controls. Knockdown experiment was performed in SW480^*PCAT2*-KI^ cells on week 16 for 72 hours. All data are shown as means ± SD. *P < 0.05, **P < 0.01, ***P < 0.001, and ****P < 0.0001.

To test this hypothesis, we next introduced the lncRNA *PCAT2* gene to a naïve chromosome locus. We identified the 4q31 locus that lacks lncRNA genes and ectopic CENP-A, with a unique sequence, present on a recognized fragile site (*65*), as an ideal site to introduce the *PCAT2* gene (Fig. 5A and fig. S16). We worked with the National Cancer Institute (NCI) Genome Modification Core facility to introduce the cytomegalovirus (CMV) promoter–driven *PCAT2* transgene array (file S1) to the 4q31 locus of SW480 cells using CRISPR and established a stable cell line (SW480^*PCAT2*-KI^ colon cancer cells) (Fig. 5B and fig. S17). Expression change of lncRNA *PCAT2* and other essential genes involved in this study was assessed using RNA sequencing from the SW480^*PCAT2*-KI^ colon cancer cells (fig. S18A). Expression of *CENP-C* and *cMYC* mRNAs remain unaltered. However, we observed a slight increase in *CENP-A*, which could be stress-induced (fig. S18B).

The transgene *PCAT2* locus in SW480^*PCAT2*-KI^ cells acquired significantly higher levels of CENP-C comparable to the SW480^WT^ cells (Fig. 5, C and D). Initially, the CENP-C levels at the native 8q24 locus were reduced, which could be due to the cellular stress caused by CRISPR and the selection procedure. However, at week 24, the CENP-C level at the Trans*PCAT2* locus has outcompete the native 8q24 locus, which is a fourfold increase compared to the 4q31 locus of SW480^WT^ cells (Fig. 5D and figs. S18, C to G, and S19). These data demonstrate that the expression of a particular oncogenic noncoding RNA gene can recruit and retain CENP-A at a naïve locus.

Last, we were curious to test whether Trans*PCAT2* at the novel chromosome 4q31 site maintains CENP-C at its locus by acting in cis, similar to the original noncoding RNA at its native site on 8q24 (Fig. 3F). To examine this possibility, we first performed a positive control, knocking down both loci expressing *PCAT2* RNAs using an ASO that targets *PCAT2* RNA in the shared sequence region for 72 hours in the SW480^*PCAT2*-KI^ cells (fig. S20). As expected, CENP-C levels of both the native 8q24 and Trans*PCAT2* locus decreased substantially (Fig. 5E). Next, using an ASO targeting the unique 31–base pair (bp) sequence tag we had incorporated into the 5′ end of the Trans*PCAT2* RNA, we selectively down-regulated only the Trans*PCAT2* RNA. In this experiment, CENP-C signals at the innate 8q24 locus remained unaffected, but only the CENP-C levels at the Trans*PCAT2* locus decreased (Fig. 5E and fig. S20). These results suggest that the Trans*PCAT2* RNA acts in cis, not in trans. In addition, it suggests that an identical copy of an oncogene expressed from a different chromosome cannot act in trans to rescue the CENP-C levels at its allelic counterpart locus. Last, we examined R-loop occupancy of the Trans*PCAT2* locus. We observed a significant colocalization of Trans*PCAT2* with R-loops (54%), at levels very similar to the native 8q24 locus (57%) (note S2 and fig. S25, A and B).

Together, these cumulative data suggest that a similar tethering mechanism of action, as we observed for the native 8q24 locus above, can be recapitulated at the Trans*PCAT2* locus for ectopic recruitment of CENP-A/C. These results imply that a specific oncogenic noncoding RNA tethering to the locus from where it is transcribed plays an integral role in the deposition and maintenance of CENP-A/C domains even in ectopic sites in the cancer epigenome.

## DISCUSSION

CENP-A overexpression and its ectopic localization are observed in many cancer types (*27*, *28*, *30*, *34*), yet the mechanism is poorly understood. Moreover, the CENP-A ectopic sites are predominantly present in genomic regions such as high transcription turnover sites and subtelomeric/telomeric regions (*27*). What has not been addressed is why only certain types of transcriptionally active sites retain CENP-A, whereas others do not. In our decades-long pursuit of this question, we observe that, of most of the ectopic CENP-A sites in these colon cancer cells, only a handful are stable, 8q24 being a prime example (*29*). The 8q24 locus is a well-documented genomic region known to be involved in amplification and translocation in several cancers (*37*). This locus has only one coding gene, the proto-oncogene *cMYC*, in an otherwise gene desert that is enriched with several oncogenic noncoding RNA genes (*42*).

Here, we show that the loss of 8q24-derived lncRNAs significantly reduces the ectopic CENP-A at the 8q24 locus, which is a locus-specific event. CENP-A loading to the centromeric DNA is transcription-dependent, and noncoding transcripts arising from centromeric alpha-satellite repeats are essential for the maintenance of the CENP-A levels at centromeres in several species (*21*, *23*, *51*–*53*). Our data imply that moderately expressed oncogenic noncoding transcripts can serve as a recruitment signal for CENP-A at ectopic sites. This interpretation is supported by a physical association of lncRNA *PCAT2* with CENP-A. Further, the knockdown of H3.3 chaperones HIRA and DAXX along with *PCAT2* prevents the ectopic CENP-A deposition at the 8q24 locus. Typically, CENP-A associates with HJURP to get deposited to the centromeres (*16*). However, in cancer cells, loss of HJURP results in higher ectopic CENP-A levels (*29*, *66*). Moreover, in vitro transcribed *PCAT2* RNA did not associate with CENP-A tetramer or nucleosome in reconstitution assays. While this may, in part, result from missing factors, or loss of correct folding of the RNA in vitro, another plausible explanation is that *PCAT2* RNA is affiliated with CENP-A only in specific chromatin contexts in vivo. We find that a naïve locus in which we inserted the *PCAT2* transgene cassette, when expressed, can recruit CENP-A/C in cis.

Recently, it has been shown that satellite-derived noncoding RNAs are localized in spatial proximity to centromeres and pericentromeres for their function (*67*). Similarly, several noncoding RNAs are localized to their site of transcription to form a hub, demarcating a nuclear compartment, for interchromosomal associations and gene regulatory functions. However, perturbing histone Post-translational modifications (PTMs) is shown to affect the gene regulatory function of lncRNAs within their nuclear territory, although it does not affect the lncRNA’s localization at its transcriptional locus (*67*). It is possible that although *PCAT2* binds histone H3, proper folding of the RNA and specific PTMs in the CENP-A protein might be critical for *PCAT2*’s association with CENP-A and co-chaperoning function. Alternatively, stable hybrid nucleosomes containing CENP-A and H3.3 particles have been documented in cancer cells for which structural analysis indicates unusual stability and elasticity (*3*, *27*, *56*, *57*). Our RIP experiments in which *PCAT2* copurifies both with CENP-A and H3 in vivo, but not in vitro, may suggest *PCAT2* association with hybrid chromatin structure at the 8q24 ectopic site. Further, *PCAT2* association with CENP-A and H3 shows that *PCAT2* has some level of specificity toward histone and histone binding proteins. These data do not exclude mass action as a plausible mode by which CENP-A co-opts H3.3 pathways to couple to this lncRNA in the cancer cells (*3*, *29*).

A working model arising from our data is that lncRNA *PCAT2* or other RNAs of this type can mimic the function of centromeric lncRNAs in the context of aberrant formation of a CENP-A:HIRA/DAXX complex, in lieu of the CENP-A:HJURP complex. This CENP-A:HIRA/DAXX complex misleads CENP-A for ectopic deposition at the 8q24 locus (*21*), but using pathways that mimic native CENP-A deposition at centromeres. In addition, HIRA, possibly DAXX, can bind single-stranded DNA (ssDNA) during transcription and repair mechanism for H3.3 deposition (*68*). Therefore, it is possible that the CENP-A:HIRA/DAXX complex can bind the ssDNA during transcription, perhaps at the site of R-loops, to deposit CENP-A ectopically. It is notable that of the five 8q24-derived noncoding transcripts, *PCAT2* is expressed at moderate levels relative to the more ubiquitously expressed *CCAT1*, and yet appears to recruit the most CENP-A/C in these colon cancer cell lines. We interpret these data as consistent with a previous landmark observation that CENP-A/C occupancy is driven by moderate expression levels at a human artificial chromosome with titratable expression levels of alpha-satellite repeats (*69*). Our data also support recent breakthroughs that have implicated R-loops in the acquisition of centromeric CENP-A in plants and yeast (*51*). We think that this novel mimicking mechanism of oncogenic lncRNAs enables the ectopic deposition of CENP-A by exploiting aberrant chaperone pathways. We can recapitulate all our observations on the native 8q24 with a simple *PCAT2* transgene engineered into a naïve chromosome locus that was originally depleted in ectopic CENP-A/C. This noncoding transgene, when transcribed in the background of native CENP-A overexpression, gains and maintains CENP-A/C stably in cis at the engineered locus, which could also accumulate R-loops. The transgene *PCAT2* effectively outcompetes the native 8q24-*PCAT2* over time. These data suggest that specific oncogenic noncoding genes and their transcription/transcripts can recruit CENP-A to any region of the genome, potentially underlying the formation of new centromeres over time.

**Fig. 6.**
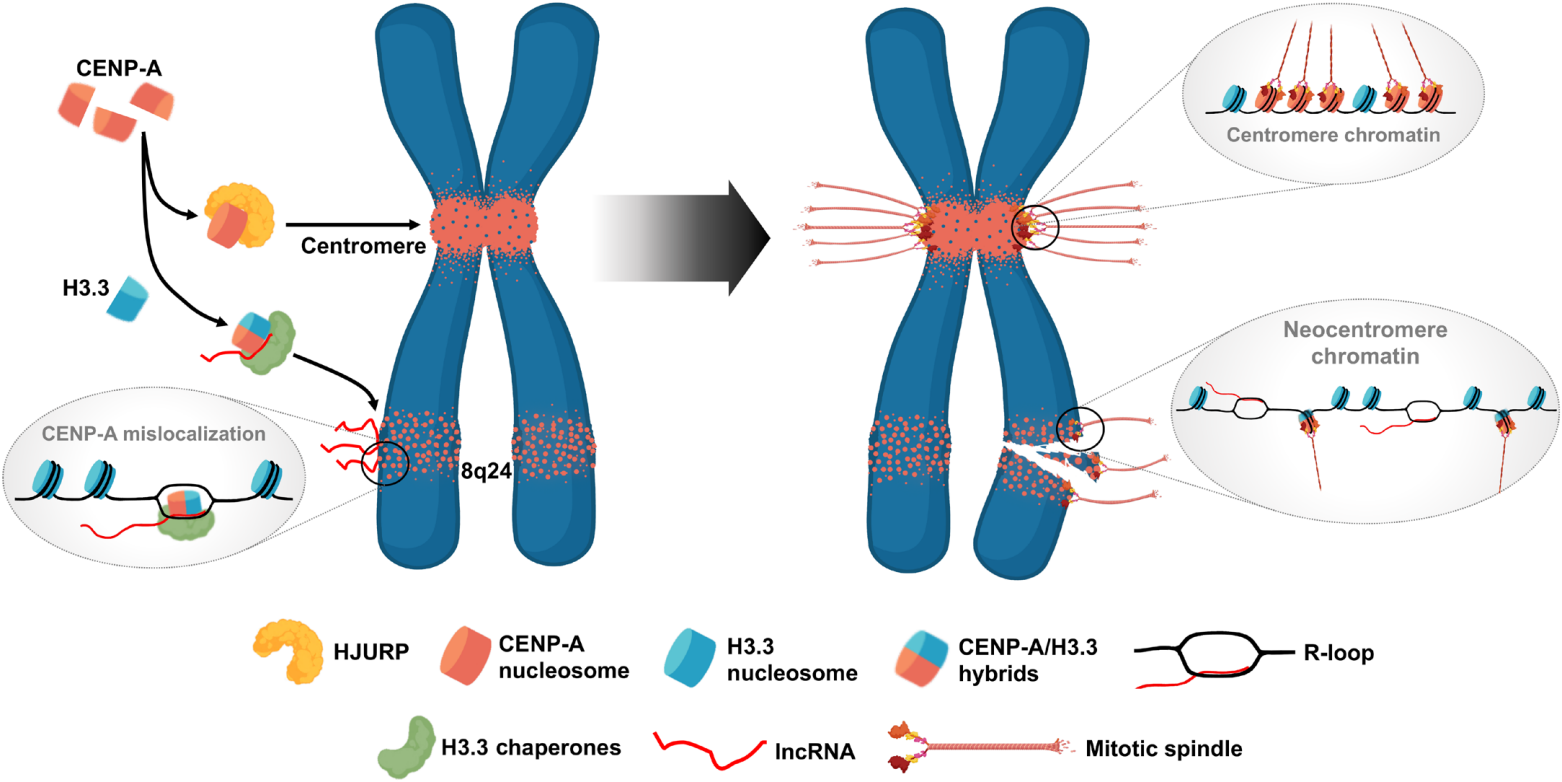
Oncogenic lncRNAs confer chromosome fragility by mislocalizing CENP-A. Usually, CENP-A associates with its chaperone HJURP to deposit at centromeres. Overexpressed CENP-A in cancer cells possibly form hybrid nucleosomes with H3.3 and hijacks the H3.3 chaperone pathways to deposit ectopically, thus invading regions such as the 8q24 locus and altering the local chromatin landscape. The 8q24-derived oncogenic lncRNAs could serve as a recruitment signal for incorrect chaperone-histone variant complexes. The CENP-A at ectopic sites promotes active transcription of the local chromatin as a feedback mechanism, leading to a higher R-loop level. The R-loop tethered lncRNAs, in turn, could help CENP-A ectopic deposition. Further, the unresolved R-loop configuration with the ectopic CENP-A nucleosomes may impair DNA replication efficiency, resulting in an under-replicated DNA with stalled replication forks. When the cells enter mitosis amid chromosomes with decondensed chromatin regions, with R-loops and ectopic CENP-A presence, these regions can build a weak ectopic kinetochore, resulting in chromosome breaks during segregation.

Genome integrity is challenged by numerous endogenous events, including transcription (*2*). R-loops that are formed during transcription in cis occupy 5% of the mammalian genomes (*70*). R-loops play an essential role in centromeric CENP-A maintenance and kinetochore integrity, ultimately accounting for chromosome stability (*51*, *71*–*74*). We speculate that a three-pronged battle between transcription, R-loop occupancy, and CENP-A invasion could challenge the mechanical integrity of the 8q24 locus DNA backbone, placing it under physical strain (*75*, *76*). Furthermore, unresolved R-loops are thought to contribute to stress during replication, leading to the persistence of under-replicated regions that can undergo breaks (*70*, *77*). Thus, this battle on the chromatin template could lead to DNA breaks, thereby compromising chromosome integrity (modeled in Fig. 6).

Our report provides key insights into the unexpected relationship between oncogenic lncRNAs and CENP-A mislocalization that contributes to chromosome fragility in human cancer. Critical next steps will be to focus on small-molecule inhibitors, which can exploit the clinical significance of this unusual interaction as a therapeutic target in human cancer cells.

## MATERIALS AND METHODS

### Cell culture

SW480 human colon cancer cells were grown in a humidified 37°C incubator containing 5% CO_2_, in RPMI 1640 medium containing glutathione and high concentrations of vitamins (catalog no. 11875093, Gibco, USA) supplemented with 10% fetal bovine serum (FBS) (catalog no. S11050H, Atlanta Biologicals, USA) and 1× penicillin-streptomycin solution (catalog no. 15140122, Gibco, USA). We have been continuously culturing this cell line for a decade to study the ectopic CENP-A occupancy and karyotypic abnormalities.

### ASOs and transfection

Locked nucleic acid (LNA) GapmeR ASOs were custom-designed and purchased from Qiagen, USA. List of the sequences of LNA ASOs used in this study was provided in table S4. Transfection of the LNA ASOs was done using Amaxa Cell Line Nucleofector Kit V (catalog no. VVCA-1003, Lonza, USA) following the manufacturer’s protocol. Briefly, the ASOs were added to 100 μl of the Nucleofector solution and incubated at room temperature for 5 min. The ASO Nucleofector solution was then mixed with 2.5 million cells, and transfection was performed using the Aluminum Nucleofector Cuvettes with the recommended transfection program. After transfection, the cells were grown in RPMI 1640 medium for 48, 72, 96, and 120 hours as per the experimental condition design. CRISPR-dCas9-KRAB plasmid with guides were transiently transfected into the cells using Lipofectamine 3000 (catalog no. L3000001, Invitrogen, USA) using the manufacturer’s protocol.

### RNA extraction and quality control

RNAs were extracted by TRIzol reagent (catalog no. 15596026, Ambion, USA) according to the manufacturer’s protocol. One T175 flask of cells was resuspended in 1 ml of TRIzol and incubated at room temperature for 5 min. Cells were aspirated five times using a 2-ml syringe with a 19-ml needle. The cell debris was spun down at 12,000 rpm for 10 min at 4°C, and the supernatant was transferred to a fresh tube. For 1 ml of TRIzol, 200 μl of chloroform was added and incubated for 2 min at room temperature. After centrifugation at 12,000 rpm for 15 min at 4°C, the clear phase was transferred to a fresh tube and mixed with 500 μl of isopropanol (catalog no. 534021, Sigma-Aldrich). The samples were centrifuged again, and the RNA pellet was washed with 1 ml of cold 75% ethanol and resuspended in diethyl pyrocarbonate ultrapure water (KD Medical, USA). The RNA samples were subjected to DNase I treatment to avoid genomic DNA contamination using a DNase I kit [New England Biolabs (NEB), USA] following the manufacturer’s protocol. The integrity of RNA was verified by resolving in 1% agarose gel containing GelStar nucleic acid gel stain (catalog no. 50535, Lonza, USA) in a Mupid-One gel electrophoresis system (TaKaRa, Japan). RNA samples were purified for the second time by the TRIzol method as mentioned above and were stored at −80°C until further analysis.

### Complementary DNA synthesis

After quantifying of RNAs using a DeNovix DS-11 spectrophotometer (DeNovix Inc., USA), 2 μg of RNA was mixed with random hexamer primers and preincubated at 65°C for 5 min to denature the RNA secondary structures and placed on ice. Complementary DNA (cDNA) conversion reactions were done using a SuperScript III Reverse Transcriptase kit (catalog no. 18080085, Invitrogen, USA) following the manufacturer’s protocol. The cDNAs were diluted in nuclease-free water and stored in −20°C until use.

### Quantitative real-time polymerase chain reaction

cDNA samples were prepared using the PowerUP SYBR Green Master Mix (catalog no. A25777, Applied Biosystems, USA) following the manufacturer’s protocol. The quantitative reverse transcription PCR (qRT-PCR) was run on the StepOnePlus Real-time PCR System (Applied Biosystems, USA), and relative quantification was performed using the 2^−ΔΔCT^ method. All the real-time PCRs were performed in 20-μl volume and in triplicates. Control reactions without the template were performed to rule out nonspecific amplification (primer dimers). LncRNA *MALAT1* served as a background control for knockdown experiments. *GAPDH* and *18S rRNA* served as internal controls for coding and noncoding RNAs, respectively. Melting curve analysis was performed for all the primer sets to check the specificity of the primers. Primer sequences used in this study are listed in table S4.

### Immunoblotting and antibodies

The cell was lysed in radioimmunoprecipitation assay (RIPA) buffer supplemented with 1× protease inhibitor cocktail and centrifuged for 10 min at 10,000 rpm at 4°C. The supernatant was collected and mixed with 4× Laemmli sample buffer (catalog no. 1610747, Bio-Rad, USA), then denatured for 7 min at 95°C, and incubated on ice for 2 min. The samples were loaded into 4 to 20% Mini-PROTEAN TGX precast gels (catalog no. 4561093, Bio-Rad, USA), resolved in 1× tris-glycine SDS running buffer (#RGC-3390, KD Medical, USA), and transferred to nitrocellulose membrane using Trans-Blot Turbo Mini Transfer Packs (catalog no. 1704158, Bio-Rad, USA). The membrane was blocked in 1:1 Odyssey blocking buffer (catalog no. 92740000, LI-COR, USA) and 1× phosphate-buffered saline (PBS) at room temperature for 1 hour and incubated with primary antibody diluted in 1:1 blocking buffer and 1× PBS complemented with 0.1% Tween 20 on a rocker for overnight at 4°C. After three washes in 1× PBS, 0.1% Tween 20, the membrane was incubated with the Alexa Fluor conjugated to the secondary antibody diluted in blocking buffer complemented with 0.1% Tween 20% for 1 hour at room temperature. As mentioned previously, the membrane was washed and imaged in an Odyssey CLx scanner (LI-COR Biosciences, USA) and analyzed using Image Studio Lite (LI-COR Biosciences, USA).

All antibodies used for immunoblotting and immunofluroscence in this study are commercially available [CENP-A (catalog no. ab13939; Abcam, USA), CENP-C (catalog no. PD030, MBL, USA), CENP-B (catalog no. SC22788, Santa Cruz Biotechnology, USA), γH2A.X (catalog no. ab81299, Abcam, USA), glyceraldehyde-3-phosphate dehydrogenase (GAPDH) (catalog no. SC20357, Santa Cruz Biotechnology, USA), S9.6 (catalog no. MABE1095, Millipore Sigma, USA), and H3.3 (catalog no. SC8654, Santa Cruz Biotechnology, USA)].

### Screening for DNA repeat elements and CENP-B box–like sequences

Ectopic CENP-A domains were selected from our previous study (table S1) (*27*). Coordinate conversion to hg38 when required was performed with the liftover tool from the UCSC genome browser. Coordinates for LINEs and LTRs were taken from the RepeatMasker track in the UCSC genome browser. All coordinate intersections for feature definition and enrichment analysis were performed with bedtools v2.27.1. Heatmaps were generated with the R package pheatmap (version 1.0.12; author, Raivo Kolde).

To perform enrichment analysis of repeat elements, we segmented the genome in three interval sets from these regions: centromeres and pericentromeres, ectopic CENP-A domains, and the rest of the genome. Using repeat elements as described above, we calculated percent overlap as a summation of intervals identified using the intersectBed tool. To analyze promoter content within domains, we defined regions based on UCSC canonical transcript models, extending 2 kb upstream of transcription starts. These regions were then intersected with LTR and LINE annotations to define LTR/LINE-positive promoters. To analyze the random association of features within domains, the shuffleBed command in bedtools was used to select randomized regions, excluding centromeres, pericentromeres, and ENCODE blacklist regions, and preserving region width.

Putative CENP-B boxes were identified by motif searching with FIMO tool v5.0.4. The 17-bp motif from Masumoto *et al.* (*78*) (YTTCGTTGGAARCGGGA) was used as the basis for this search. To enable searching with this tool, International Union of Pure and Applied Chemistry (IUPAC) ambiguity codes were replaced ([CT]TTCGTTGGAA[AG]CGGGA).

### *k*-mer analysis

To identify sequence motifs present in lncRNAs that may predict ectopic CENP-A localization, we performed the *k*-mer analysis with transcript sequence of *CCAT1*, *CCAT2*, *PVT1*, *PCAT1*, and *PCAT2*. We also included alpha-satellite monomer sequence, the Cen1-like sequence identified by Henikoff *et al.*, and putative centromeric transcripts (alpha-satellite monomer transcript) previously identified by RIP sequencing (*50*, *79*). We counted all possible 6-mers using Jellyfish and performed unsupervised clustering on the results to identify patterns of sequence composition.

### Preparation of metaphase chromosomes

After transfection, colcemid (catalog no. 10295892001, Millipore Sigma, USA) was added 8 hours before the knockdown incubation end time, and the cells were harvested by trypsinization (catalog no. 25200056, Thermo Fisher Scientific, USA). The cells were washed with 10 ml of 1× PBS, suspended in 6 ml of hypotonic solution, and incubated in a water bath at 37°C for 20 min. They were fixed using freshly prepared cold fixative solution (methanol:glacial acetic acid in 3:1 proportion). They were suspended in 4 ml of fixative following centrifugation at 1500 rpm for 5 min at room temperature and incubated for 1 hour at room temperature. They were again centrifuged and resuspended in 400 μl of the fixative solution and stored at 4°C until slide preparation.

About 40 to 80 μl (two to three drops) of the fixed cells were dropped over the cold glass slide prewashed with fixative and were air-dried for 3 min. The slides were viewed under a light microscope to check the density of the metaphase spread, and the same protocol was repeated with cell dilutions using a freshly prepared fixative solution until optimal metaphase spread and density were attained. The slides were immediately processed for IF and DNA in situ hybridization experiments.

In the case of experiments performed in unfixed cells, after the 8-hour incubation with colcemid, the cells were subjected to hypotonic treatment for 20 min at 37°C. About 25,000 cells were diluted in 500 μl of hypotonic solution, and using Cytospin 4 centrifuge (Fisher Scientific, USA), the cells were pounded over glass slides by spinning at 2000 rpm (high acceleration) for 4 min at room temperature.

### Immunofluorescence

#### Methanol-fixed cells

After the cells were pounded onto the glass slide, the slide was incubated in freshly prepared KCM buffer [120 mM KCl, 20 mM NaCl, 10 mM tris-HCl (pH 8.0), and 0.5 mM EDTA] containing 0.1% Triton X-100 and 1× cOmplete Protease Inhibitor Cocktail (catalog no. 11697498001, Millipore Sigma, USA) for 15 min at room temperature. The slides were then treated with a blocking solution [KCM buffer containing 3% bovine serum albumin (BSA) and 1× protease inhibitor cocktail] for 30 min at room temperature. Following blocking, slides were incubated with the primary antibody in the antibody solution (KCM buffer containing 1% BSA and 1× protease inhibitor cocktail) for 1 hour at room temperature. The slides were washed three times with KCM buffer and incubated with an antibody solution containing Alexa Fluor–conjugated secondary antibody (Abcam, USA) in the dark for 1 hour at room temperature. Then, the slides are washed with KCM buffer four times and fixed using 10% formalin for 10 min in the dark at room temperature. After washing the slides in autoclaved distilled water, the slides were dehydrated in 70, 95, and 100% ethanol and air-dried for 2 min. The slides were then mounted with coverslips using the ProLong Gold Antifade Mountant with 4′,6-diamidino-2-phenylindole (DAPI)–containing mounting solution (catalog no. P36935, Thermo Fisher Scientific, USA) or advanced for DNA-FISH protocol as required.

#### Unfixed cells

After the metaphase chromosomes were dropped onto the glass slides, the slides were immediately incubated in the TEEN buffer [1 mM triethanolamine-HCl (pH 8.5), 0.2 mM Na-EDTA, and 25 mM NaCl] three times. The slides were then blocked in the TEEN-containing blocking buffer (0.1% Triton X-100 and 0.1% BSA in the TEEN buffer) for 30 min at room temperature. Following blocking, slides were incubated with primary antibody diluted in the TEEN buffer for 1 hour at room temperature. The slides were then washed three times with KB buffer [10 mM tris-HCl (pH 7.7), 150 mM NaCl, and 0.1% BSA] and incubated with Alexa Fluor–conjugated secondary antibody in KB buffer for 40 min at room temperature in the dark. After washing the slides three times with KB buffer, the slides were fixed using 10% formalin for 10 min in the dark at room temperature. The slides were washed three times in autoclaved distilled water and air-dried before mounting with DAPI-containing mounting solution or processed for DNA-FISH protocol as required.

For experiments that involved ribonuclease H (RNase H) treatment, the metaphase spread was prepared on the glass slide and incubated in the TEEN buffer immediately for 5 min at room temperature. RNase H was prepared as per the manufacturer’s protocol (catalog no. M0523S, NEB, USA). RNase H solution was added to the glass slides and incubated in a 37°C incubator for 15 min. The RNase H activity was stopped by washing the slides with 0.5 M EDTA for 5 min at room temperature. The slides were then subjected to blocking and incubation of primary and secondary antibodies, as mentioned above.

### DNA fluorescence in situ hybridization

#### Probe preparation

For 100 μl of DNA-FISH probe preparation, 2 μg of bacterial artificial chromosome (BAC) DNA (RP11-N13 for the 8q24 locus), 1× final concentration NT buffer [0.5 M tris-HCl (pH 8.0) and 50 mM MgCl_2_], 0.1 M β-mercaptoethanol (catalog no. 60-24-2, Millipore Sigma, USA), 0.5 mM ACG mix [deoxyadenosine triphosphate (dATP), deoxycytidine triphosphate (dCTP), and deoxyguanosine triphosphate (dGTP)] (catalog no. N0446S, NEB, USA), 0.1 mM deoxythymidine triphosphate (dTTP), 0.5 mM digoxigenin-16-dUTP (deoxyuridine triphosphate) (catalog no. 495-34, Dyomics, Germany), and 3 μl of DNase (1:100 dilution; NEB, USA) were added and made up to 99 μl. One microliter of DNA polymerase I (catalog no. 10642711001, Millipore Sigma, USA) was added, mixed gently, and incubated in Eppendorf Thermomixer R (Fisher Scientific, USA) at 15°C for 1.10 hours with 330 rpm. Immediately after the incubation, the probe mix was placed on ice, and 8 μl of probe DNA was run in 1.5% agarose gel to test the size of the probes (optimal size between 300 and 700 bp). Following size confirmation, 1 μl of 0.5 M EDTA (pH 5.2) was added to the probe reaction mix and heat-inactivated at 75°C for 10 min in the dark.

For each slide, 5.5 μl of DNA-FISH probe, 20 μg of human COT-1 DNA (catalog no. 11582011103, Roche Custom Biotech, USA), 20 μg of shredded salmon sperm DNA (catalog no. AM9680, Invitrogen, USA), and 0.3 M sodium acetate (pH 5.5) were mixed and made up to 20 μl using nuclease-free water. About 2.5 volumes of cold 100% ethanol were added to the mix and incubated at −20°C for 20 min. The probe mix was then centrifuged at 12,000 rpm for 30 min at 4°C. The supernatant was discarded, and the DNA-FISH probe pellet was air-dried in the dark for 20 min. The DNA-FISH probe pellet was resuspended in 6 μl of DNA hybridization buffer [50% formamide and 10% dextran sulfate in 2× SSC buffer (saline-sodium citrate)] and denatured at 80°C for 5 min followed by preannealing at 37°C for 1 hour in the dark. In the 2q21 and 10p15 locus cases, DNA-FISH probes conjugated with Carboxytetramethylrhodamine (5-TAMRA) were procured commercially (Empire Genomics, USA).

#### Hybridization

After the slides were prepared as mentioned previously, the slides were equilibrated in 2× SSC for 5 min at room temperature and digested with pepsin (10 mg/ml; catalog no. 10108057001, Millipore Sigma, USA) in 2× SSC solution for 3 min at 37°C. The slides were immediately washed in 2× SSC three times and dehydrated in ethanol series (70, 95, and 100%). Following dehydration, the slides were denatured in 70% formamide in 2× SSC at 80°C for 5 min and dehydrated once again in ethanol series as mentioned in the previous step. The DNA-FISH probe was added to the slides, covered with a coverslip, and sealed with rubber cement (catalog no. 72170, EMS, USA). The slides were incubated in a humidified chamber at 37°C overnight. Later, the coverslip was gently removed, and the slides were incubated at 45°C in 2× SSC containing 50% formamide for three times 5 min each. Last, the slides were washed four times in 0.2× SSC at 65°C for 5 min each and washed once in 2× SSC for 5 min at room temperature. The slides were air-dried and mounted using DAPI-containing mounting solution and allowed to dry for 2 hours at room temperature. All the hybridization protocols were performed in the dark.

### Microscopy and image analysis

DNA-FISH and IF slides were imaged in a DeltaVision Elite RT microscopy imaging system (GE Healthcare, USA) with a charge-coupled device camera (CoolSNAP, USA) mounted on an inverted microscope (IX-70, Olympus, USA). On average, 30 metaphase chromosome spread images for each experiment were captured by using a 60× objective (oil) with 0.1-μm z-sections, deconvolved, and analyzed with ImageJ (version 1.51 with Java 1.8.0_172). Colocalization of proteins and/or protein at DNA-FISH sites on metaphase chromosome was identified using the “Colocalization” plugin in ImageJ software. Colocalization readings from innate and translocated 8q24 loci were pooled and presented for all experimental conditions. For protein load, the signal intensity from the IF image was measured using ImageJ and normalized against the background signal.

### RNA immunoprecipitation

Cells were treated with 1% formaldehyde in 1× PBS containing 0.1% Tween for 10 min to cross-link the proteins bound to RNA. A final concentration of 125 mM glycine was added to the cells and washed twice with 1× PBS containing 0.1% Tween and 10 mM Ribonucleoside Vanadyl Complex (RVC) (#S1402S, NEB, USA). The pellet was washed with cold TM2 buffer containing NP-40 (catalog no. FNN0021, Invitrogen, USA) and RVC and resuspended in 0.1 M Tris-Ethylenediaminetetraacetic acid (TE buffer). The cell pellet was then treated with Micrococcal nuclease (MNase) (2 U/ml) for 6 min, and chromatin was extracted overnight using a low-salt buffer with 1× cOmplete Protease Inhibitor Cocktail (catalog no. 11697498001, Millipore Sigma, USA) at 4°C. The supernatant collected was incubated with a CENP-A antibody overnight at 4°C. The CENP-A antibody–bound nucleic acids were pulled down using Dynabeads Protein G–tagged magnetic beads (catalog no. 10003D, Invitrogen, USA) in the low-salt buffer. The nucleic acid–bound beads were then subjected to RNA extraction using TRIzol reagent. The RNA extracted was then treated with DNase I and reextracted using TRIzol reagent. The isolated RNA was subjected to *rRNA* depletion using the NEBNext rRNA Depletion Kit (catalog no. E6350, NEB, USA) following the suppliers’ protocol and used for cDNA synthesis for semiquantitative and real-time qRT-PCR experiments.

### Plasmid construction and cloning

Gene blocks (gBlock) of lncRNA *PCAT2*^CDS^ and CMV^promoter^, each ranging from 150 to 500 bp, were synthesized commercially (IDT Inc., USA). The length of the gBlocks was based on the GC% and repeat lengths. Each gBlock ends were designed with a palindrome sequence of BSA I restriction enzyme (RE) and restricted overnight using BsaI-HFv2 (catalog no. R3733, NEB, USA). The restricted gBlocks were purified using the Zymo DNA Clean and Concentrator Kit (catalog no. D4033, Zymo Research, USA). The purified gBlocks were cloned into pUC19 plasmid using Golden Gate assembly protocol as described in NEB protocols online. Three separate plasmids carrying CMV^promoter^-*PCAT2*^CDS^ with different restriction ends at 3′ and 5′ ends were made. Last, the cloned full-length fragment of CMV^promoter^-*PCAT2*^CDS^ from three plasmids was released with appropriate RE digestion, resolved in gel, and gel-eluted using the Zymoclean Large Fragment DNA Recovery Kit (catalog no. D4045, Zymo Research, USA). The eluted three full-length CMV^promoter^-*PCAT2*^CDS^ fragments carrying different REs (fragment 1, 3′-Esp3 I-Frag1-BamH I-5′; fragment 2, 3′-BamH I-Frag2-Hind III-5′, and fragment 3, 3′-Hind III-Frag3-Xba I-5′) were cloned into the pTet-tTS vector (catalog no. 631011, TaKaRa Bio Inc., USA) backbone digested with Esp3 I and Xba I. The final cloned p3x_*PCAT2* (with the pTet backbone) plasmid carried three copies of CMV^promoter^-*PCAT2*^CDS^ as an array. Neomycin-resistant gene with RSV^Promoter^ was synthesized and cloned into the plasmid p3x-*PCAT2* containing three copies of CMV^promoter^-*PCAT2*^CDS^ using Kas I and BstE II sites. Plasmid p3x-*PCAT2* was subjected to RE digestion and resolved to confirm the cloning experiment at every step. Specific primer sets were used to PCR amplify and sequence the plasmid to confirm the cloned sequences and order of the fragments, including the selection marker.

### CRISPR knock-in and generation of stable cell line

The plasmid p3x-*PCAT2* carrying 3× CMV^promoter^-*PCAT2*^CDS^ with NeoR was linearized using BsrG I and BstP I restriction sites. A fragile site, FRA4Ctel, present in chromosome 4 between chr4:139,499,746 and 140,592,371 (GRCh38/hg38), which has a 150-bp DNA segment unique to chromosome 4, was selected as the target site for CRISPR knock-in. Guide RNA specific to this sequence was designed and cloned in the CRISPR plasmid containing the puromycin selection marker. The CRISPR plasmid, along with the p3x-*PCAT2* plasmid containing neomycin selection marker, was transfected in SW480 colon cancer cells using Lipofectamine 2000 (catalog no. 11668019, Invitrogen, USA) following the manufacturer’s protocol. Transfected cells were grown for 1 day in 20% FBS–containing culture medium, followed by 10% FBS–containing culture medium for 1 day before adding the desired concentration of selection drugs. The cells were selected for 10 days using dual drugs, and on the 10th day, six single cells were collected to grow individually (separate wells) to form a homogeneous colony. The colonies (SW480^*PCAT2*-KI^ colon cancer cells) were propagated in large culture flasks and used for downstream experiments.

### Whole-genome sequencing

DNA was isolated from SW480^*PCAT2*-KI^ colon cancer cells using phenol:chloroform:isoamyl alcohol method. The quality of the DNA was tested using 2100 Bioanalyzer (Agilent, USA). DNA was sequenced on NovaSeq 6000 Standard SP run using TruSeq Nano DNA Library Prep (Illumina, USA) and paired-end sequencing mode. The sample was mapped, and variants were called using DRAGEN (Illumina, USA). The insert sequence was identified from the whole-genome sequencing reads using a blast search algorithm against a custom reference with the insert plasmid sequence.

### RNA sequencing

For RNA expression profiling, total RNA was isolated from SW480^*PCAT2*-KI^ colon cancer cells. The quality of the RNA was determined by the 2100 Bioanalyzer (Agilent, USA). Sequencing libraries were constructed from total RNA samples with the Illumina TruSeq Stranded Total RNA Library Prep Kit (RS-122-2201). Libraries were multiplexed and sequenced on one lane of an Illumina HiSeq2500 instrument using TruSeq V4.0 chemistry and sequenced for 126 cycles in paired-end mode. Raw reads were preprocessed to remove low-quality bases and adapter sequences using Trimmomatic. Trimmed reads were mapped to the human genome (hg19) with Tophat2 v2.0.8. The following parameters were used: mate inner distance, 10 bp; SD, 200 bp; library type, first strand.

To calculate differential expression, read counts within genes were generated with HTSeq, against the Ensembl release 75 annotations. Differential expression was calculated with DESeq and is reported at a significance threshold of false discovery rate–adjusted *P* value of <0.01. Transcript abundance estimates were reported in units of RPKM (reads per kilobase of transcript per million reads mapped).

### ChIP sequencing

Five flasks of SW480^*PCAT2*-KI^ colon cancer cells were grown in the T175 flasks and harvested using trypsin. The cells were washed with PBS, followed by one wash with cold PBST (0.1% Tween). The pellet was washed with cold TM2 buffer containing NP-40 (catalog no. FNN0021, Invitrogen, USA) and resuspended in 0.1 M TE. The cell pellet was then treated with MNase (4 U/ml) for 15 min in a 37°C water bath, and chromatin was extracted overnight using a low-salt buffer with 1× cOmplete Protease Inhibitor Cocktail (catalog no. 11697498001, Millipore Sigma, USA) at 4°C. The supernatant collected was incubated with our custom CENP-A antibody (rabbit polyclonal, Epitope: C-TPGPSRRGPSLGA) overnight at 4°C. The CENP-A antibody–bound nucleic acids were pulled down using Dynabeads Protein G–tagged magnetic beads (catalog no. 10003D, Invitrogen, USA) in the low-salt buffer. The nucleic acid–bound beads were then subjected to RNase I treatment (NEB, USA) and DNA extraction using the phenol-chloroform protocol. The quality of the DNA was tested using 2100 Bioanalyzer (Agilent, USA). The DNA was sequenced in NextSeq (Illumina, USA) using 75-bp paired-end reads. Data were processed, normalized, and analyzed as mentioned in the previous work (*27*).

### Statistical analysis

All the numerical data are presented with SD in graphs. The number of loci was counted for colocalization, and the intensity of the IF signals was presented with *P* values in table S5. The differences between means from the colocalization signal intensities were analyzed by two-tailed Student’s *t* test (Mann-Whitney) and two-way analysis of variance (ANOVA) for groups. Fisher’s exact test was used to analyze the difference between groups in the case of colocalizing foci counts. All statistical differences were calculated using GraphPad Prism software (v7.7e, GraphPad Software Inc., USA). A *P* value of <0.05 was considered as a statistically significant difference. Illustrative diagrams were created using BioRender (BioRender, USA) and Photoshop CC 2019 (Adobe Inc., USA).

## Supplementary Materials

### This PDF file includes:

Figs. S1 to S20 Tables S1, S4 and S5 Notes S1 and S2 File S1 References

## Notes

View/request a protocol for this paper from *Bio-protocol*.
