# Supplementary material for: Oncogenic lncRNAs alter epigenetic memory at a fragile chromosomal site in human cancer cells
Source: Sci Adv. 2022 Mar 2;8(9):eabl5621. doi: 10.1126/sciadv.abl5621 (PMC8890707; doi:10.1126/sciadv.abl5621)
Supplement: Supplementary file 1 — Figs. S1 to S20 Tables S1, S4 and S5 Notes S1 and S2 File S1 References [file sciadv.abl5621_sm.pdf]

Science Advances
AAAS

Supplementary Materials for

Oncogenic lncRNAs alter epigenetic memory at a fragile chromosomal site in human cancer cells

Ganesan Arunkumar, Songjoon Baek, David Sturgill, Minh Bui, Yamini Dalal*

*Corresponding author. Email: dalaly@mail.nih.gov

Published 2 March 2022, *Sci. Adv.* **8**, eabl5621 (2022)
DOI: 10.1126/sciadv.abl5621

The PDF file includes:

Figs. S1 to S20
Tables S1, S4 and S5
Legends for tables S2 and S3
Notes S1 and S2
File S1
References

Other Supplementary Material for this manuscript includes the following:

Tables S2 and S3

Fig. S1.

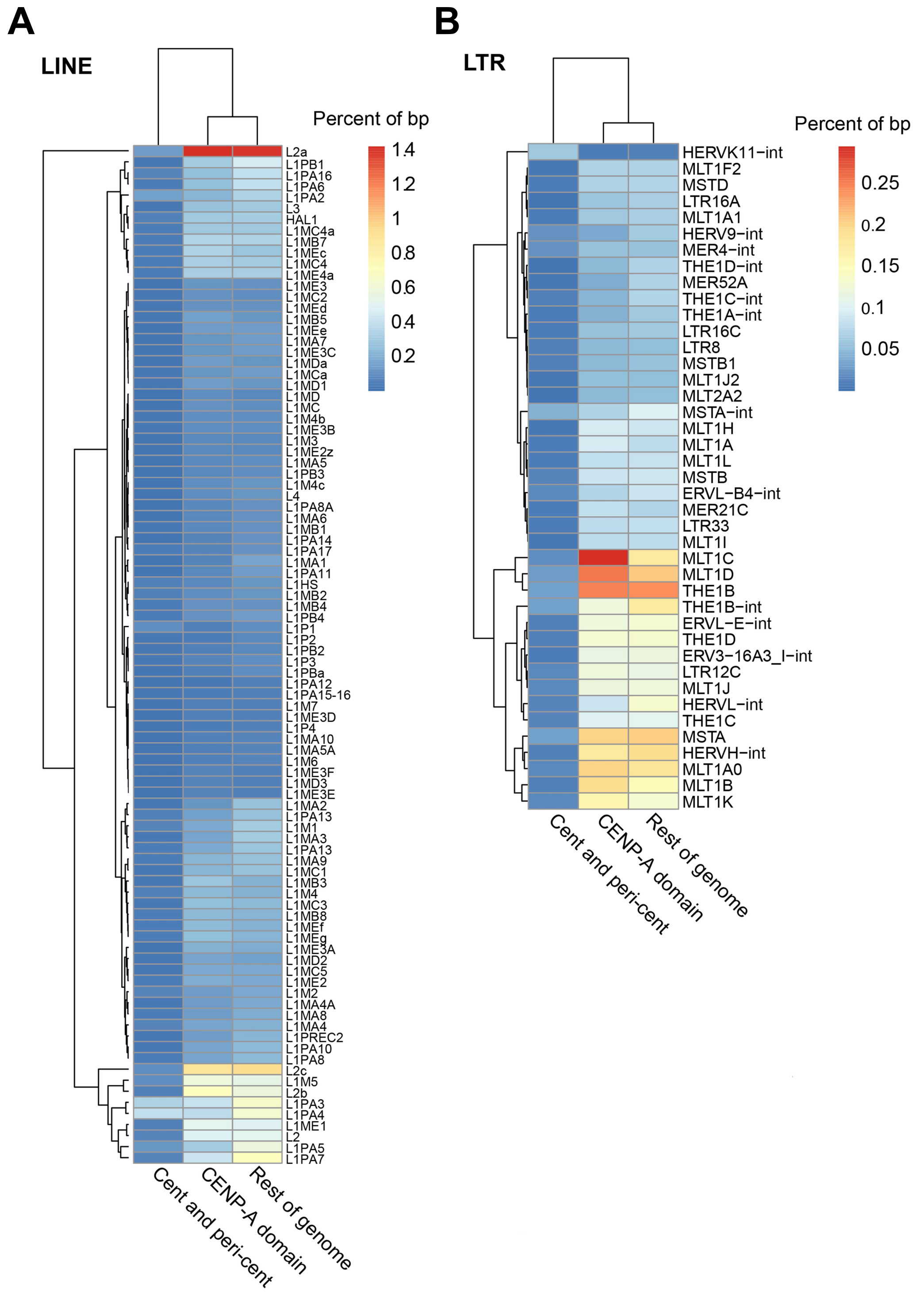

Fig. S1. Heatmaps of repeat element density: Three large genomic segmentations are analyzed to identify the repeat enrichment, centromeres and pericentromeres, ectopic CENP-A domains, and the rest of the genome. Data shown are the total percent of each of these three segmentations that overlap annotated repeat elements of from two broad repeat families. **A.** Hierarchical clustering and heat map of LINE element percentages. LINE elements where the maximum representation within a segmentation is at least 0.05% are shown. LINE element L2a is enriched in CENP-A domain and non-CENP-A regions of the genome. **B.** Hierarchical clustering and heat map of LTR element percentages. LTR elements where the maximum representation within a segmentation is at least 0.05% are shown. LTR element MLT1C showed a modest (1.66-fold) enrichment and was heterogeneously present within individual CENP-A domains.

Fig. S2.

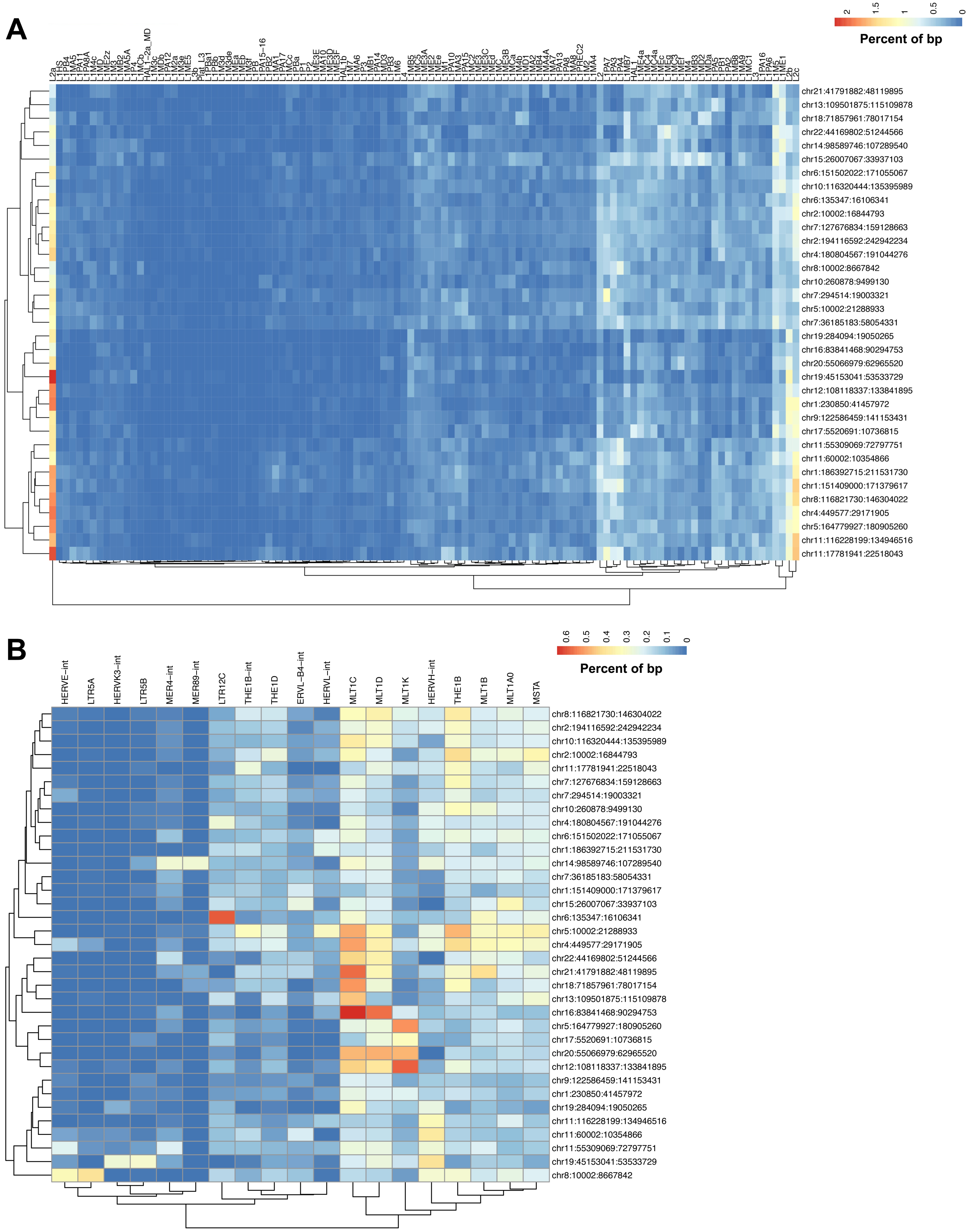

Fig. S2. Heatmaps of repeat element enrichment at CENP-A domains: A. Hierarchal clustering and heat map of LINE elements enrichment at individual ectopic CENP-A domains. **B.** Hierarchal clustering and heat map of LTR elements enrichment at individual ectopic CENP-A domains. MLT1C element showed a moderate elevation in representation within CENP-A domains.

Fig. S3.

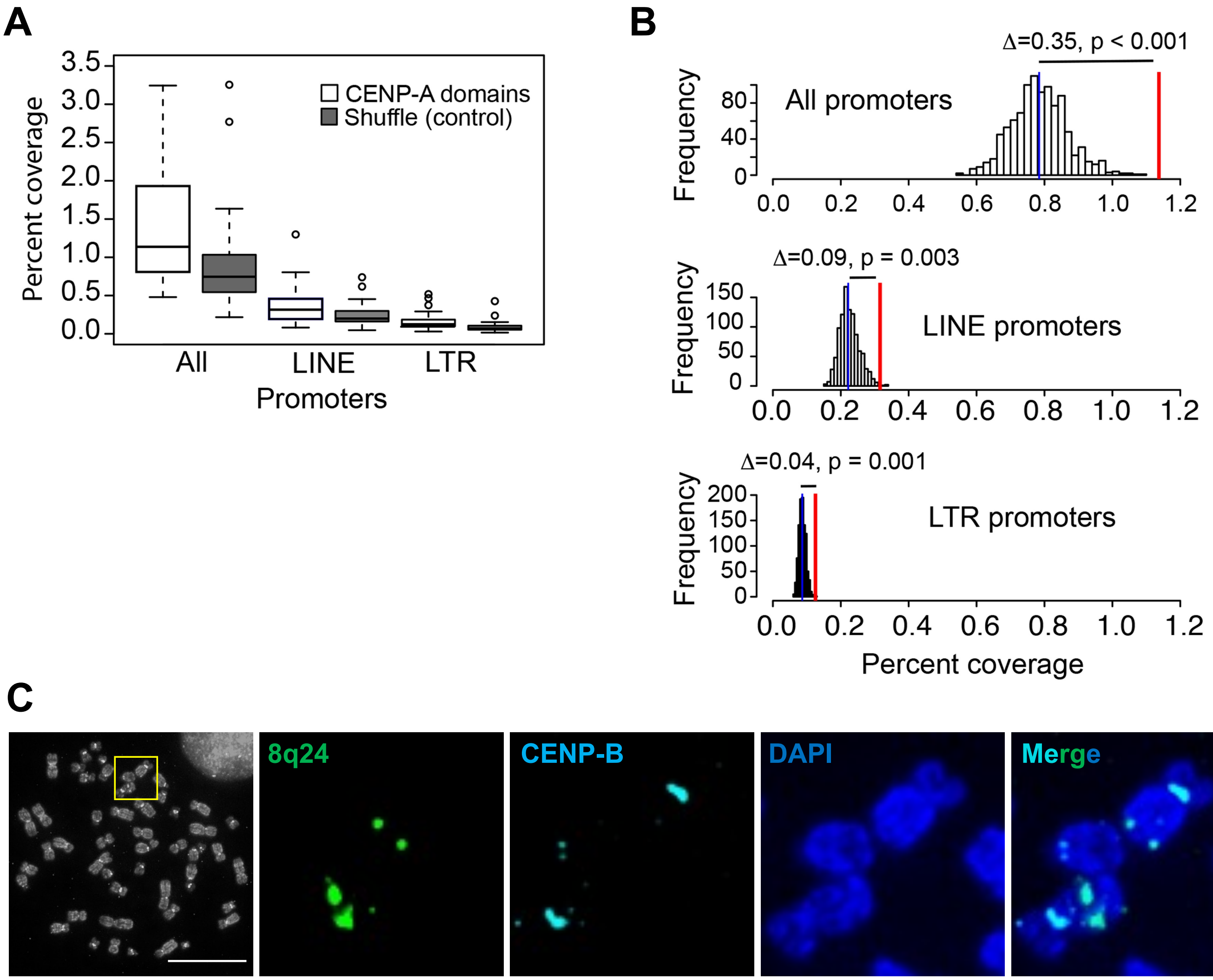

Fig. S3. Genomic analysis of CENP-A domains: A. Boxplots of the percent coverage of all promoters, and promoters with LINE or LTR, in ectopic CENP-A domains compared to shuffled control. **B.** Random expectation analysis by performing 1000 shuffles of CENP-A domains, and comparing median promoter density. The bar plot represents a histogram of median coverages obtained from these 1000 trials, with percent coverage on the X-axis. P-values represent the empirical probability of obtaining an equal or greater median value in random regions (blue line) as in non-shuffled domains (red line). Delta values are the difference between the observed median, and the median of all random trials. **C.** IF-DNA-FISH image of chromosome 8 showing CENP-B signal (Cyan) only at chromosome 8 centromere but not at the 8q24 locus (Green). Scale bar represents 10 μm.

Fig. S4.

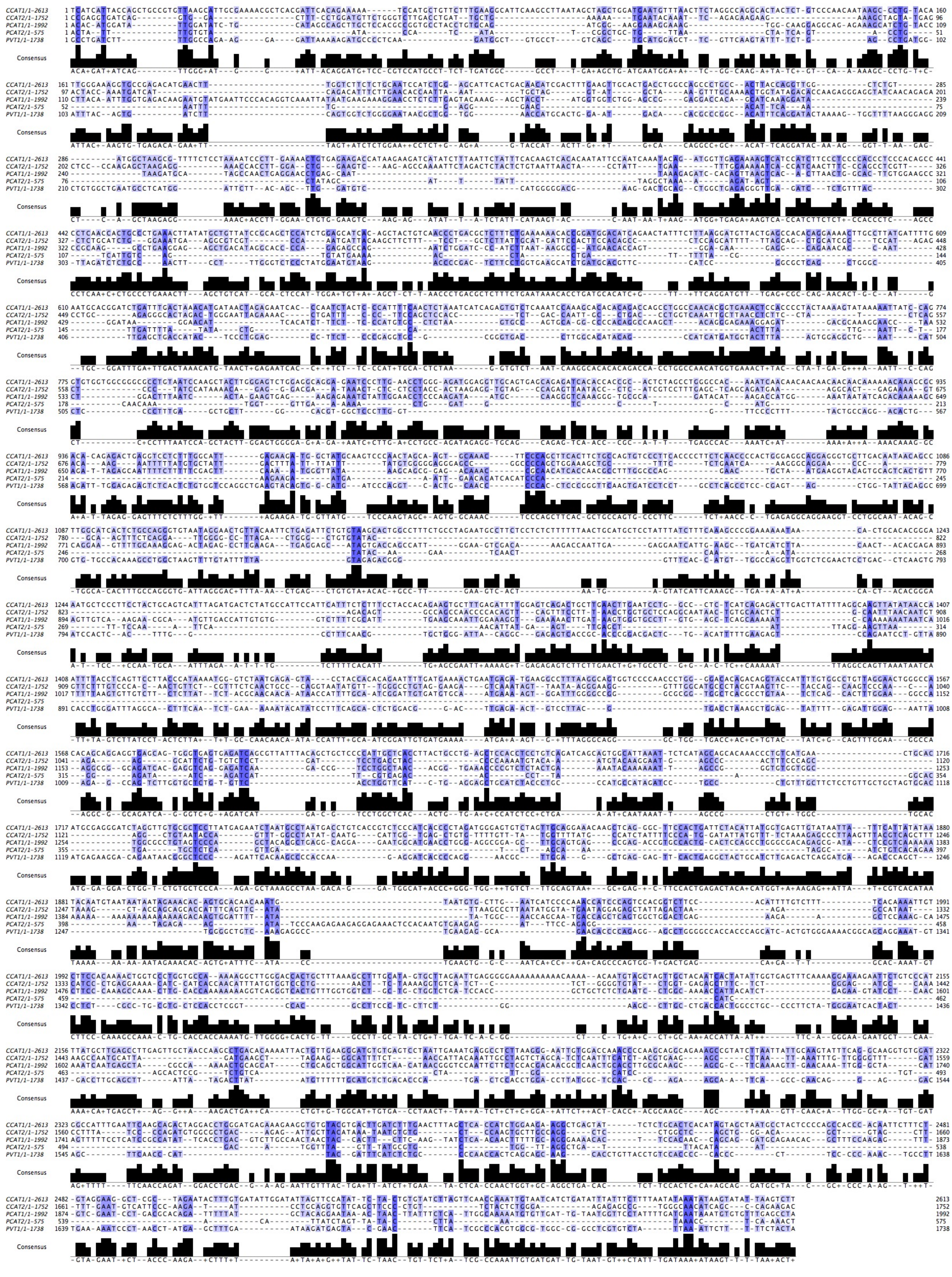

Fig. S4. Multiple sequence alignment of chromosome 8q24-derived lncRNAs: Multiple sequence alignment of the 8q24-derived lncRNAs *CCAT1, CCAT2, PCAT1, PCAT2,* and *PVT1* did not show any significant conserved sequence motifs.

Fig. S5.

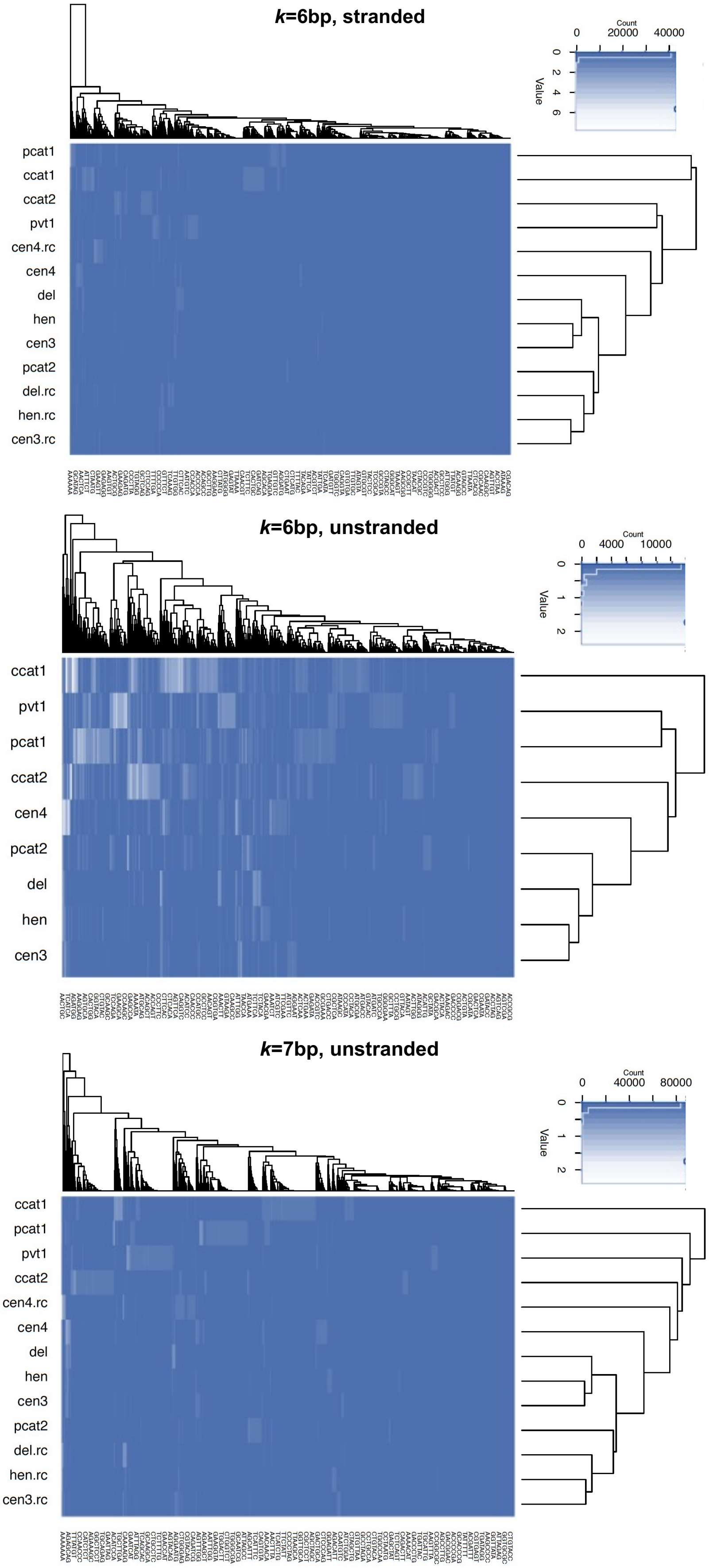

Fig. S5. *k*-mer analysis of the 8q24-derived lncRNAs and centromeric ncRNAs: Hierarchical clustering of the 8q24-derived lncRNAs with other centromere expressed lncRNAs using 6bp stranded and 6 & 7bp unstranded sequence. Sequence descriptions and *k*-mers are presented in Table S3.

Fig. S6.

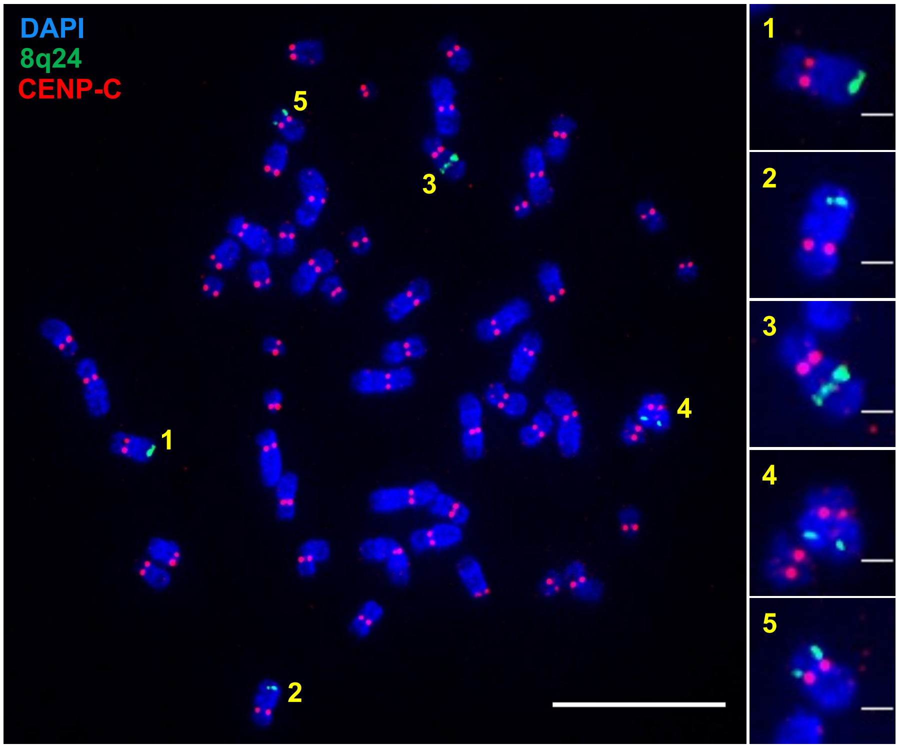

Fig. S6. Metaphase chromosome spread of SW480 colon cancer cell: A representative metaphase chromosome spread image of metastatic SW480 colon cancer cells displaying hyper diploid karyotype with 5 copies of 8q24/*cMYC* locus (Green). Two of the 8q24 locus are present in the innate chromosome (inset 1 and 2) at its sub-telomeric region and three others are translocated (inset 3 – 5). Scale bar represents 10 μm in the main image and 1 μm in insets.

Fig. S7.

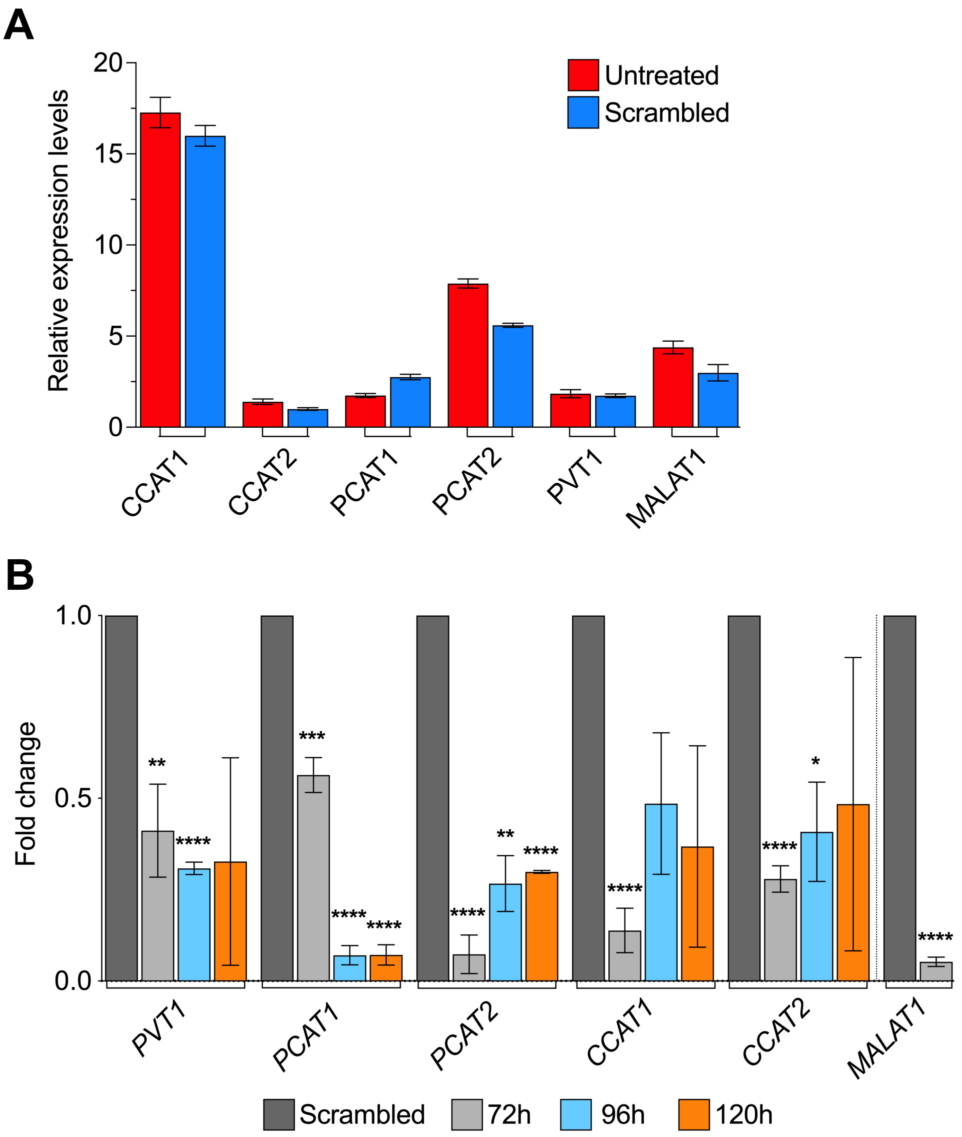

Fig. S7. Quantification of lncRNAs using RT-qPCR: A. Relative expression levels of the 8q24-derived lncRNAs (*PVT1, PCAT1, PCAT2, CCAT1,* and *CCAT2*) in untreated SW480 colon cancer cells vs Scrambled ASO transfected cells using real-time qPCR. **B.** Expression fold change of 8q24 locus derived lncRNAs after ASO-mediated knockdown compared to the scrambled control from SW480 colon cancer cells. LncRNA *MALAT1* served as a positive control to confirm the knockdown and real-time qPCR experiments.

Fig. S8.

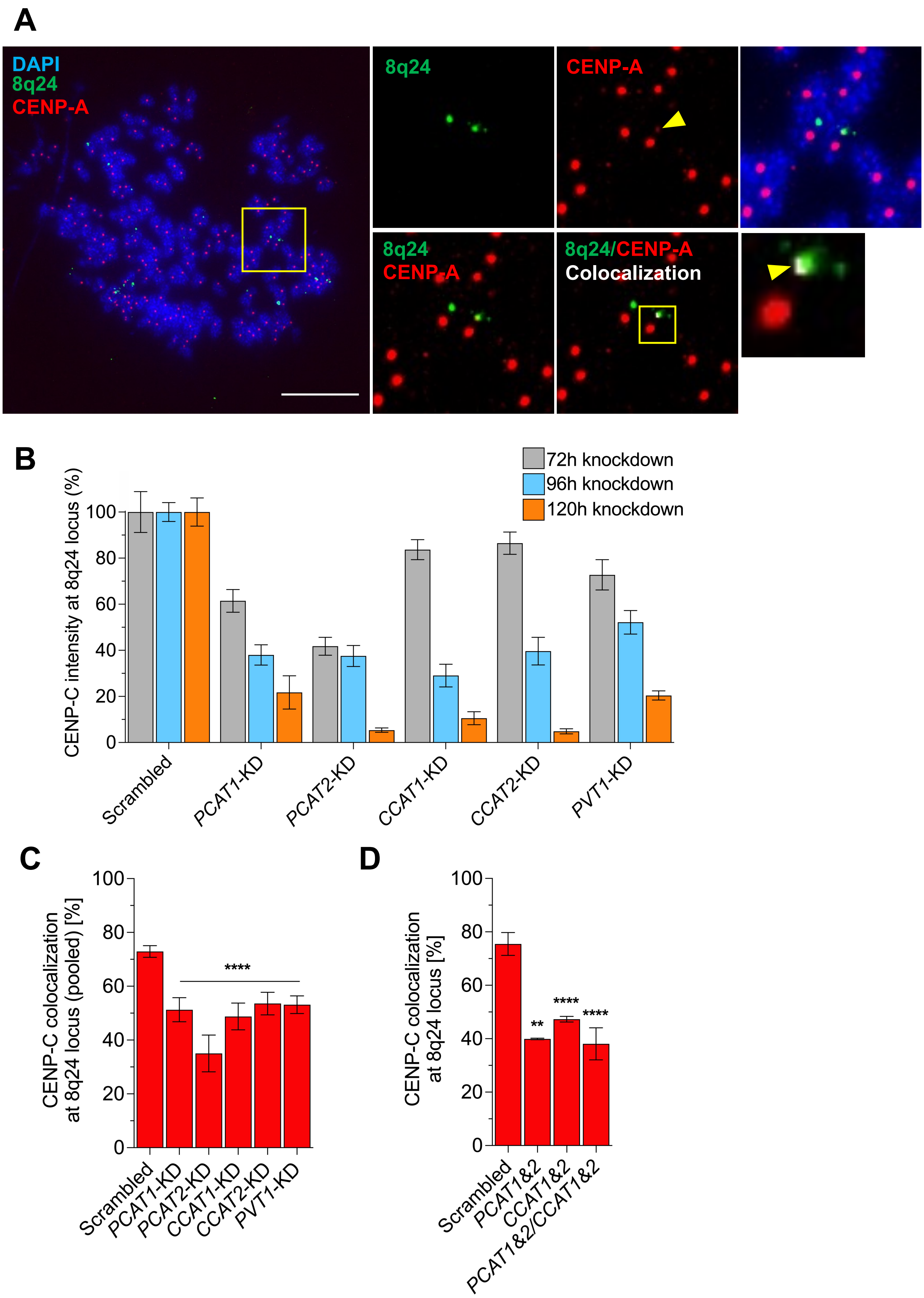
A
DAPI
8q24
CENP-A
8q24
CENP-A
8q24
CENP-A
8q24/CENP-A
Colocalization
B
72h knockdown
96h knockdown
120h knockdown
CENP-C intensity at 8q24 locus (%)
100
80
60
40
20
0
Scrambled
PCAT1-KD
PCAT2-KD
CCAT1-KD
CCAT2-KD
PVT1-KD
C
CENP-C colocalization at 8q24 locus (pooled) [%]
100
80
60
40
20
0
****
Scrambled
PCAT1-KD
PCAT2-KD
CCAT1-KD
CCAT2-KD
PVT1-KD
D
CENP-C colocalization at 8q24 locus [%]
100
80
60
40
20
0
**
****
****
Scrambled
PCAT1&2
CCAT1&2
PCAT1&2/CCAT1&2

Fig. S8. Colocalization of CENP-A/CENP-C at 8q24 locus of SW480 colon cancer cells: A. A representative unfixed metaphase chromosome spread image of metastatic SW480 colon cancer cells stained for CENP-A (Red) and 8q24/*cMYC* locus (Green). The colocalization of CENP-A at 8q24 locus (White) was detected by colocalization finder plug-in in ImageJ. The insets showing the colocalization of CENP-A at 8q24 locus marked by yellow arrow. Scale bar represents 10 µm. **B.** Intensity of CENP-C signal at the 8q24 locus after 72 h, 96 h, and 120 h knockdown of the 8q24-derived lncRNAs in SW480 colon cancer cells. **C.** Averaged CENP-C colocalization foci at 8q24 locus after knockdown of 8q24 derived lncRNAs in SW480 colon cancer cells (pooled irrespective of time point for effect comparison). **D.** Percentage CENP-C colocalization foci at the 8q24 locus of SW480 colon cancer cells after knockdown of the 8q24 locus derived lncRNAs for 72 h in combinations compared to the scrambled control. The final concentration of ASOs in combination knockdowns is maintained at the manufacturer's recommended level.

Fig. S9.

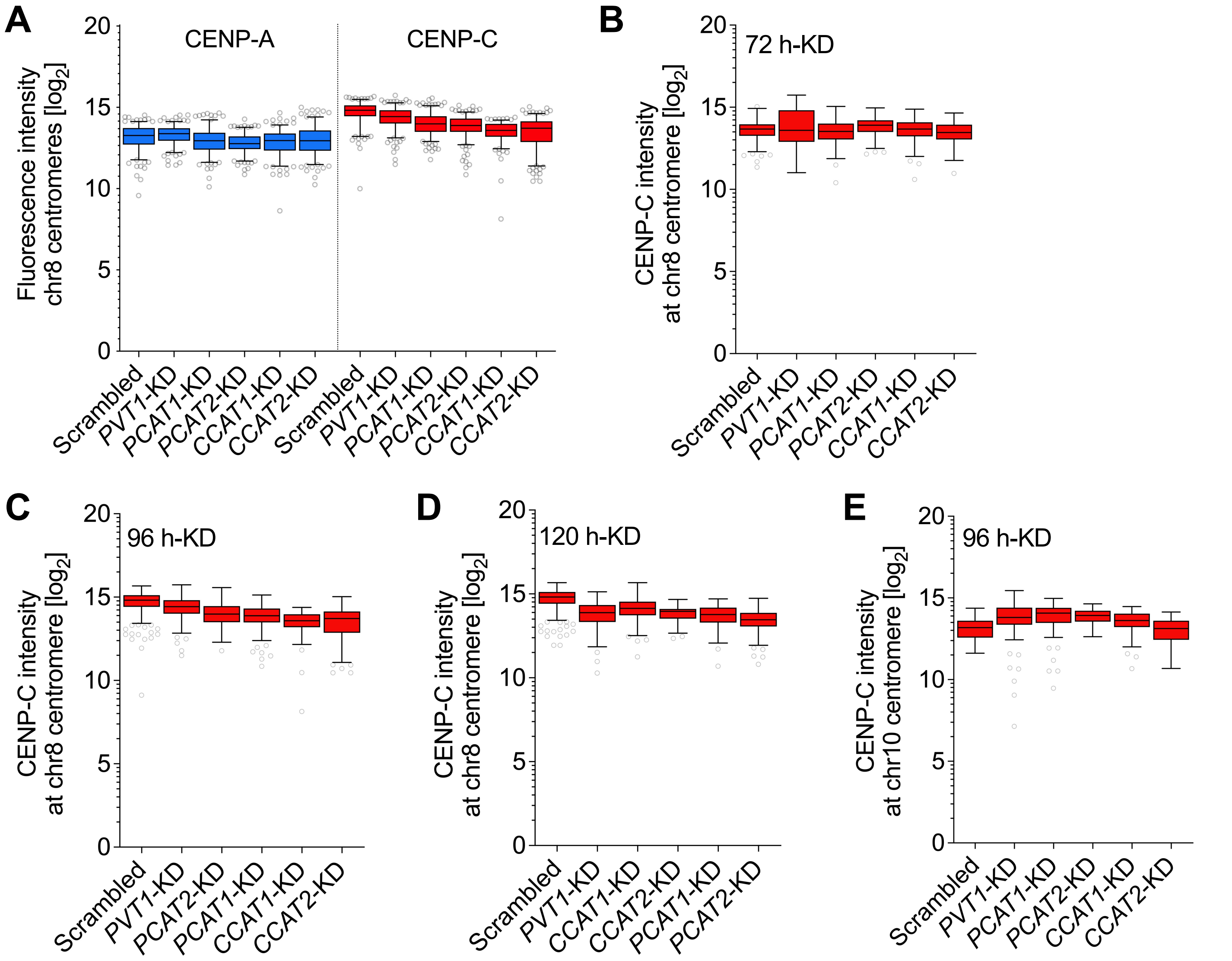

Fig. S9. Centromeric CENP-A/CENP-C levels in the lncRNA knockdown experiments: A. Fluorescence intensity of CENP-A and CENP-C at the chromosome 8 centromere in unfixed SW480 colon cancer cells after 96 h knockdown of the 8q24-derived lncRNAs compared to the scrambled control. **B to D.** Fluorescence intensity of CENP-C at the chromosome 8 centromere after 72 h (**B**), 96 h (**C**), and 120 h (**D**) knockdown of the 8q24-derived lncRNAs in SW480 fixed cells compared to scrambled control. **E.** Fluorescence intensity of CENP-C at the chromosome 10 centromere after 96 h knockdown of 8q24-derived lncRNAs.

Fig. S10.

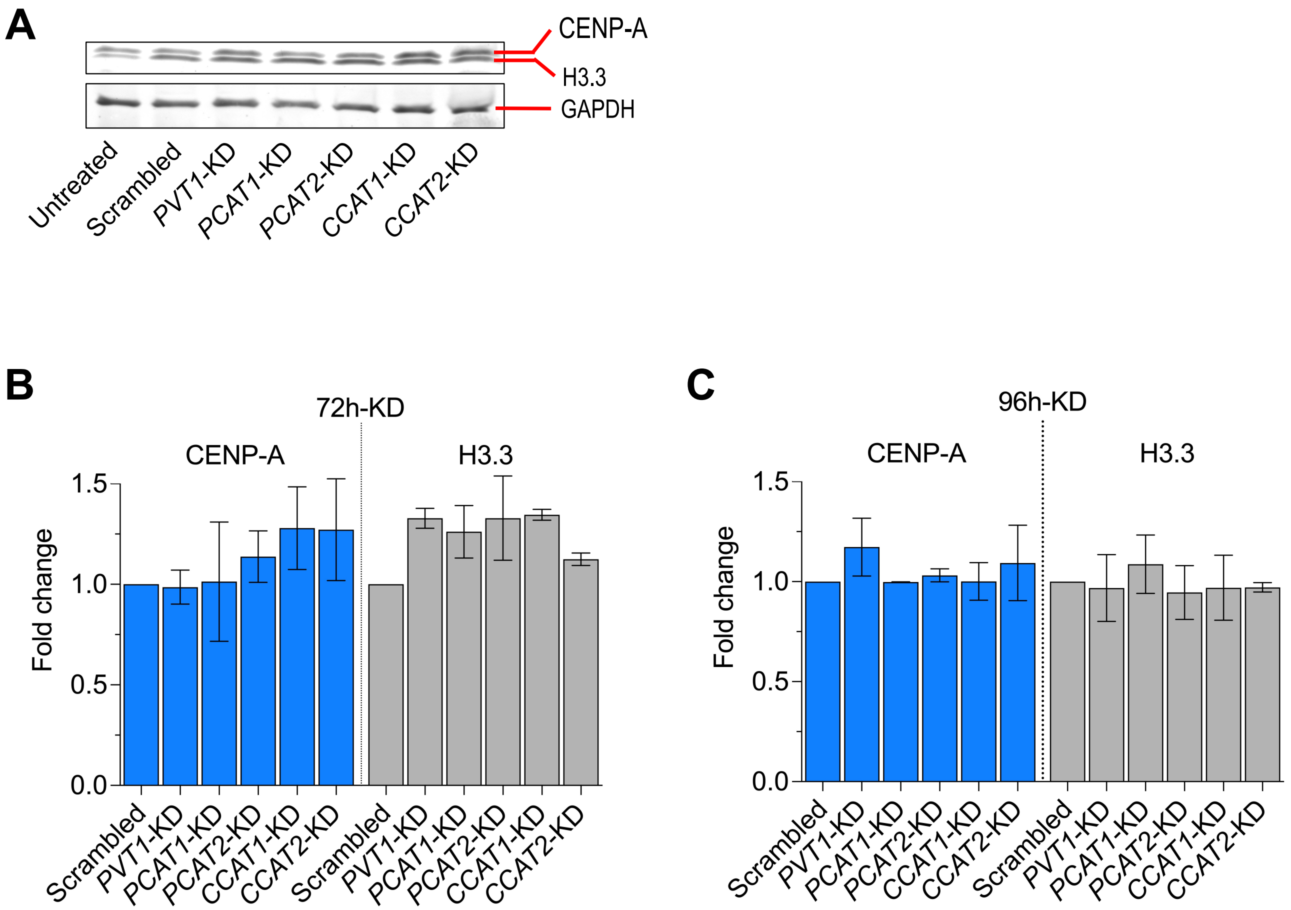

Fig. S10. Protein levels of CENP-A and H3.3 in the lncRNA knockdown experiments: A. Representative immunoblot of protein isolated from SW480 colon cancer cells probed for CENP-A, H3.3, and GAPDH proteins after 72 h knockdown of the 8q24-derived lncRNAs. **B & C.** Quantification of CENP-A and H3.3 protein levels in lncRNA knockdown samples at 72 h (**B**) and 96 h (**C**) from SW480 colon cancer cells. Both CENP-A and CENP-A are not significantly altered in the knockdown experiments.

Fig. S11.

A

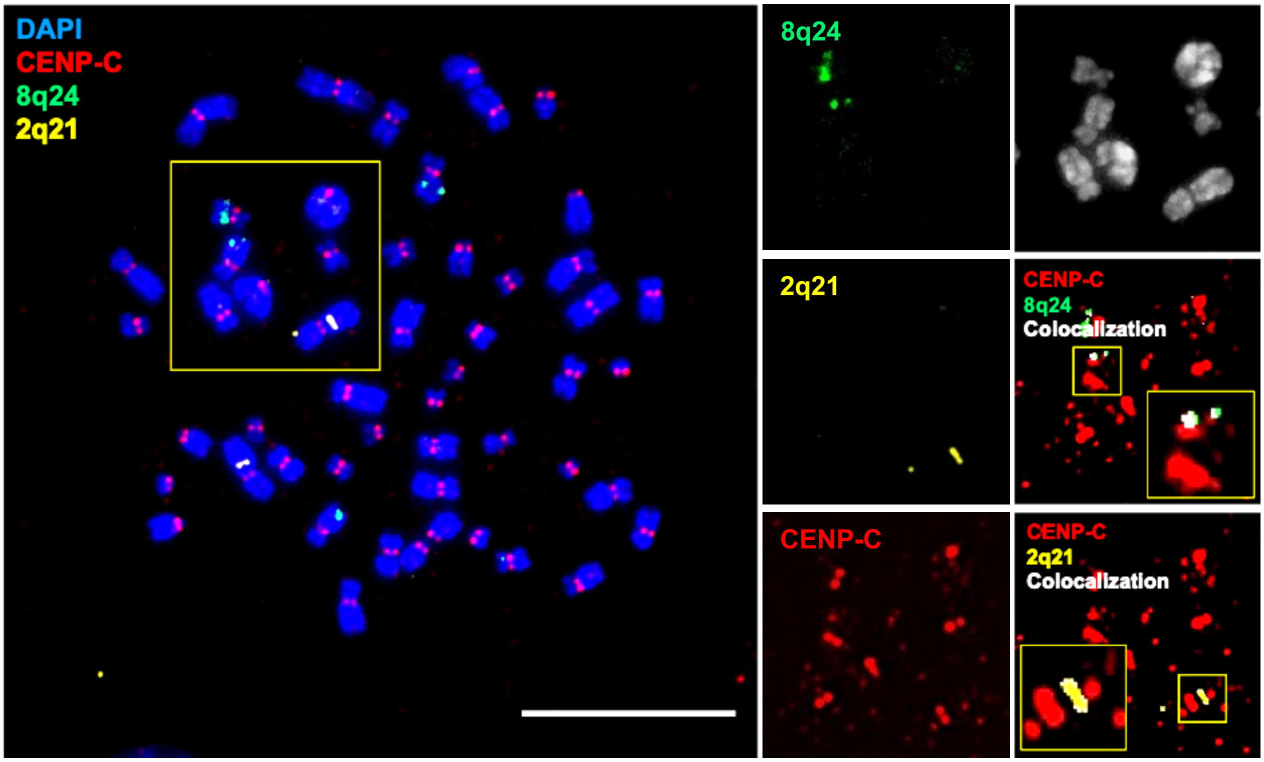

B

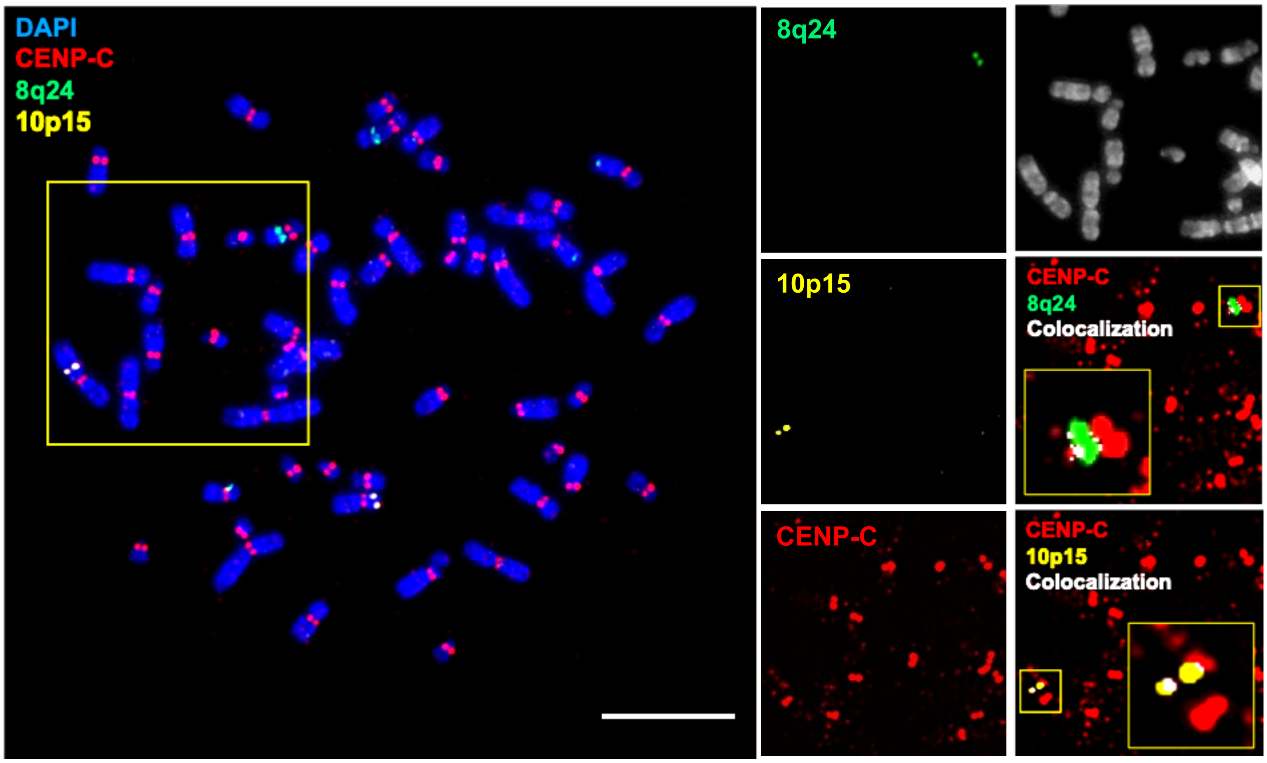

Fig. S11. IF-double DNA-FISH image of SW480 metaphase chromosomes: A. SW480 colon cancer cells probed for CENP-C (Red) by immunofluorescence, and the 8q24 (Green) and the 2q21 (Yellow) locus by DNA-FISH. **B.** SW480 cells probed for CENP-C (Red) by immunofluorescence, and the 8q24 (Green) and the 10p15 (Yellow) locus by DNA-FISH. Colocalization of the CENP-C at the probed locus is marked in White color. The CENP-C channel (Red) in the inset figures are enhanced to visualize the signal from ectopic sites. Scale bar represents 10 μm.

Fig. S12.

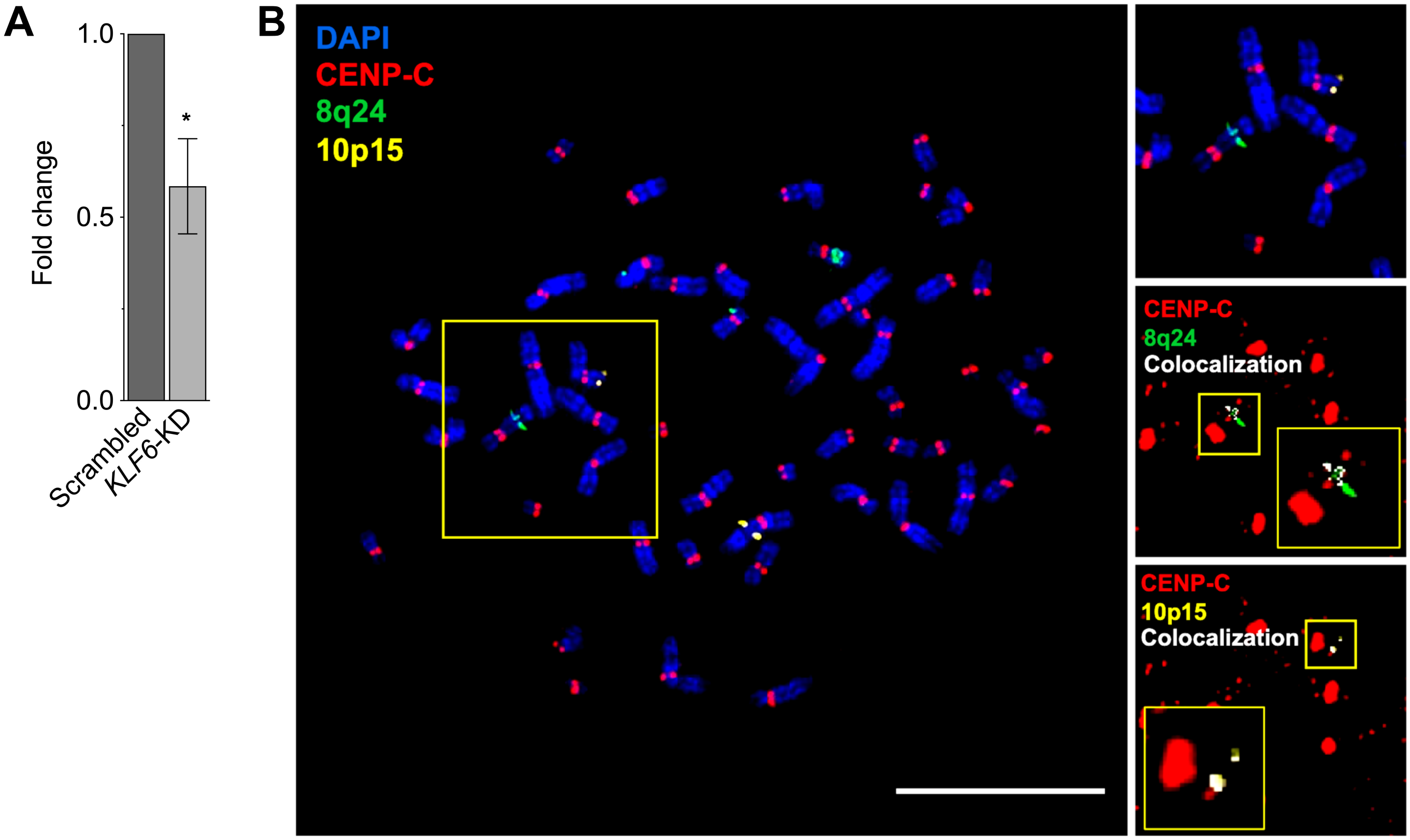

Fig. S12. Knockdown of 10p15 locus coding gene *KLF6*: A. Expression fold change of the 10p15 locus derived *KLF6* mRNA, coding gene, in SW480 colon cancer cells after 72 h knockdown of *KLF6* compared to the scrambled control (p=0.0328). **B.** SW480 colon cells probed for CENP-C (Red) by immunofluorescence, and the 10p15 (Yellow) and the 8q24 (Green) locus by DNA-FISH after knockdown of *KLF6* for 72 h. The colocalization of CENP-C at the 8q24 and the 10p15 locus are shown in inset images (White, right insets). The CENP-C channel (Red) in the inset figures are enhanced to visualize the signal from ectopic sites. Scale bar represents 10 μm.

Fig. S13.

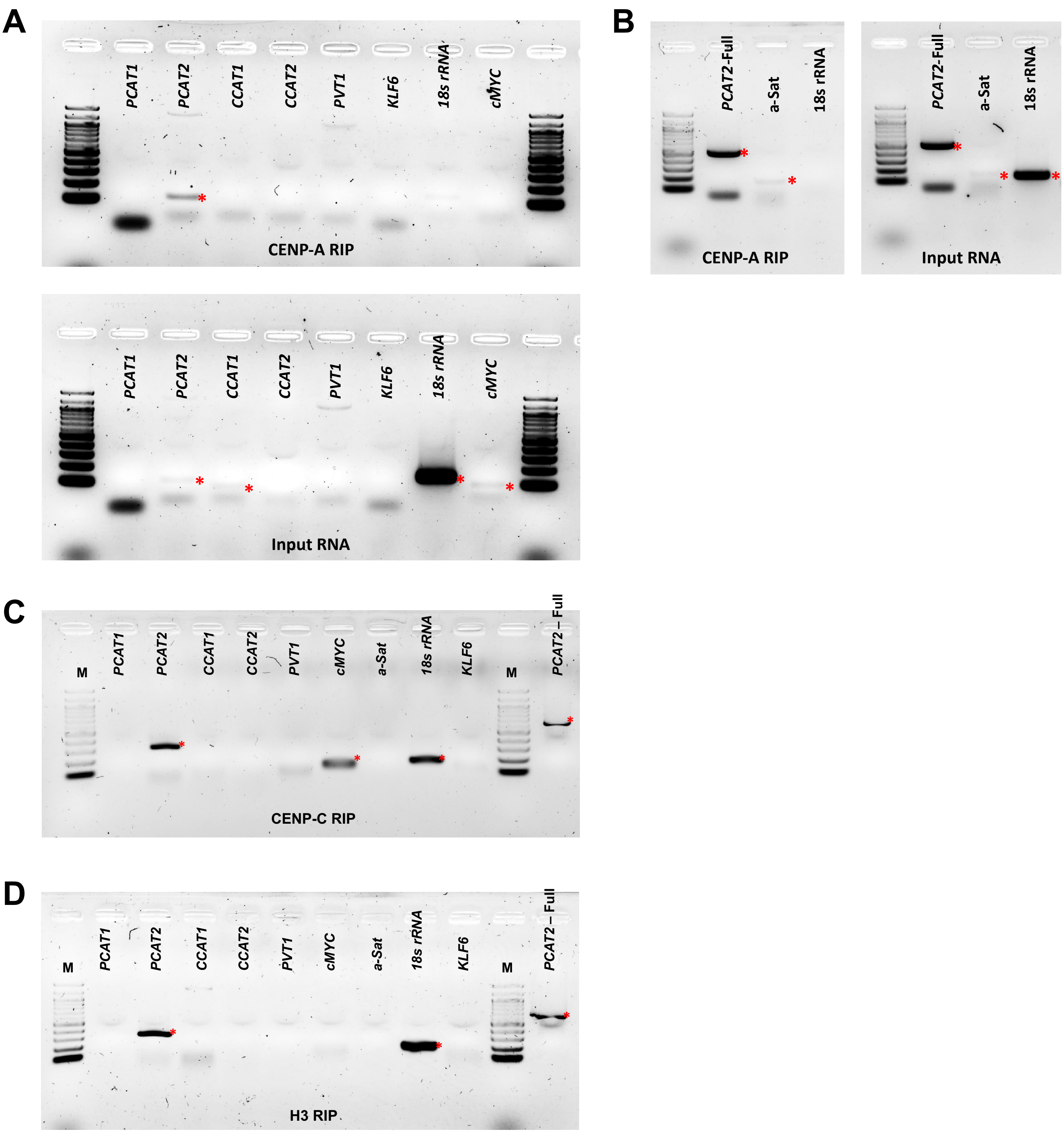

Fig. S13. RIP semi-quantitative PCR gel of SW480 colon cancer cells: A. Semi-quantitative PCR of the 8q24-derived lncRNAs, *18s rRNA*, *KLF6*, and *cMYC* in anti-CENP-A RIP (top – *rRNA* depleted) and input RNA samples (bottom). PCRs wee performed at Tm = 60°C and 30 cycles. **B.** Semi-quantitative PCR of *PCAT2* (full-length primer set, 554bp), alpha-satellite (a-Sat), and *18s rRNA* in anti-CENP-A RIP (right) and input RNA samples (left). *PCAT1, CCAT2, PVT1,* and *KLF6* bands in the input are below visual detection limit in semi-quantitative PCR gel. RIP experiments were done in replicates. **C.** and **D.** *PCAT2* is also co-purified with CENP-C and H3 in the SW480 cells. CENP-C, in addition to *PCAT2*, also binds to *cMYC* mRNA. PCRs wee performed at Tm = 60°C and 35 cycles. Red-star mark PCR positive lanes.

Fig. S14.

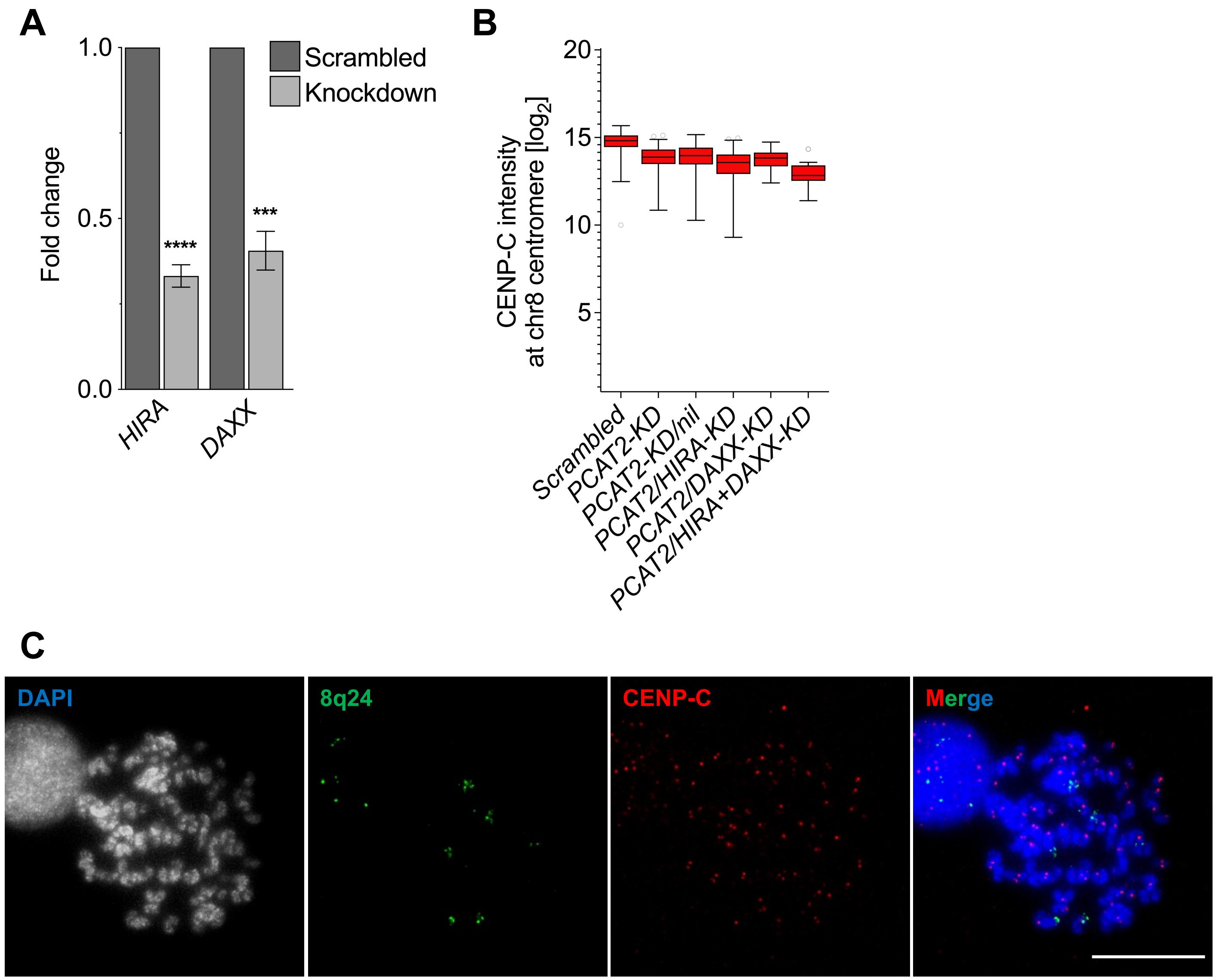

Fig. S14. Knockdown of H3.3 chaperones in SW480 colon cancer cells: A. Expression fold change of *HIRA and DAXX* mRNAs after their 72 h knockdown using ASO in SW480 colon cancer cells (p=0.0001 and 0.0005, respectively). **B.** Fluorescence intensity of CENP-C at the chromosome 8 centromeres in the sequential knockdown of *PCAT2* and H3.3 chaperones. **C.** Metaphase chromosome spread of *PCAT2/HIRA+DAXX*-KD cells showing a congested chromosome spread and deformed chromosome structure, and a significant percentage of cells (81%) were non-viable or non-proliferating. Scale bar represents 10 µm.

Fig. S15.

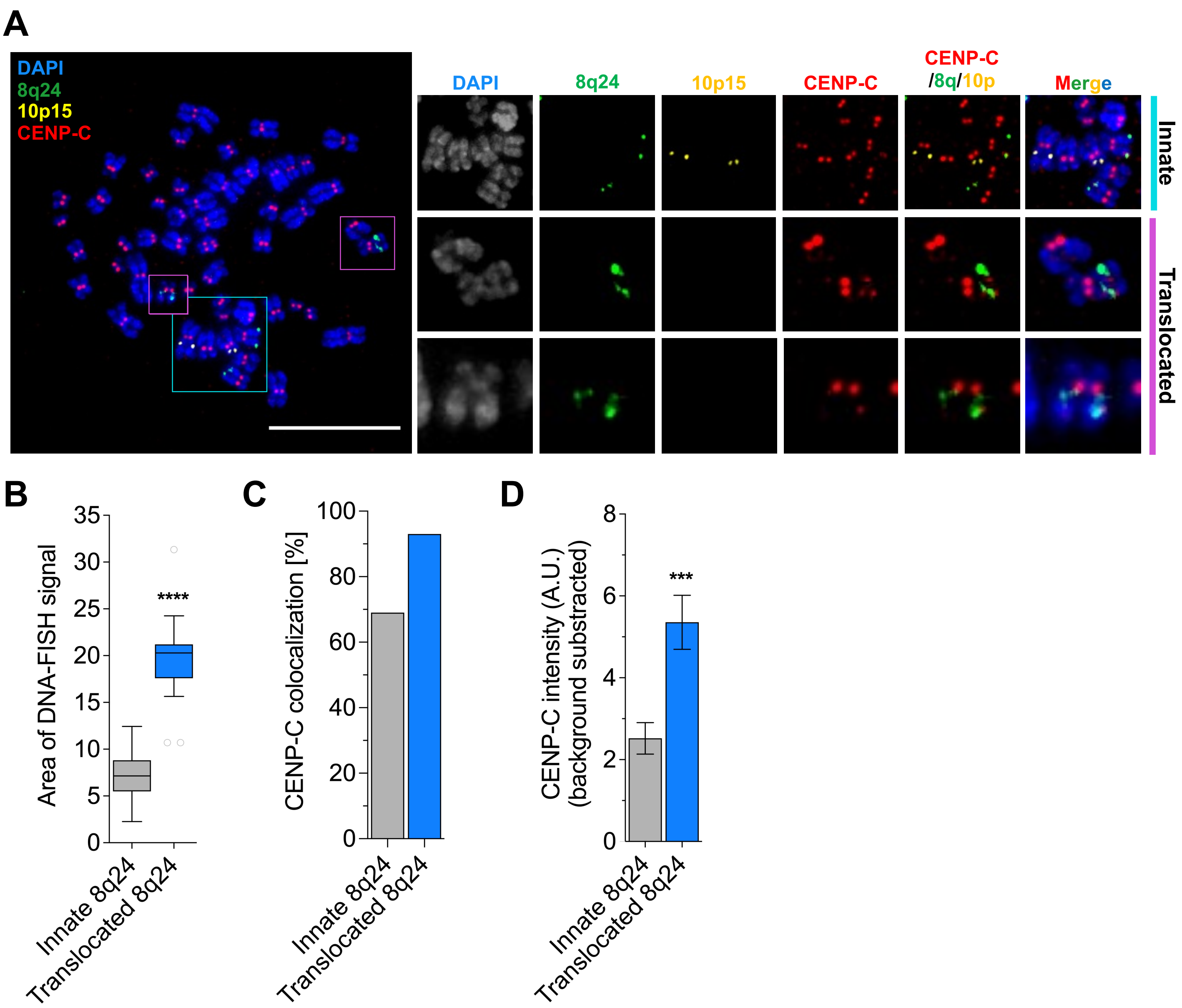

Fig. S15. CENP-C levels at the innate and translocated 8q24 locus in SW480 colon cancer cells. A. IF-double FISH of metaphase chromosomes probe for 8q24 and 10p15 loci showing the visual difference between the innate and translocated chromosome loci of the 8q24. The chromatin of the innate locus of the 8q24 and the 10p15 appears sharper and compact (Cyan box) compared to the less compact translocated 8q24 locus (Purple box). Scale bars represent 10µm. **B.** Fluorescence (DNA-FISH) signal area of the 8q24-probe bound chromatin shows a significantly higher surface area of the translocated 8q24 locus compared to the innate 8q24 locus (p<0.0001). The 8q24 locus signal area is normalized to the total area of chromosome 8 using DAPI. **C.** Percentage CENP-C colocalization foci at the innate and the translocated 8q24 locus of SW480 colon cancer cells. **D.** Histogram showing the difference in the level of CENP-C at the innate and the translocated 8q24 locus. Translocated 8q24 locus has a significantly higher level of CENP-C.

Fig. S16.

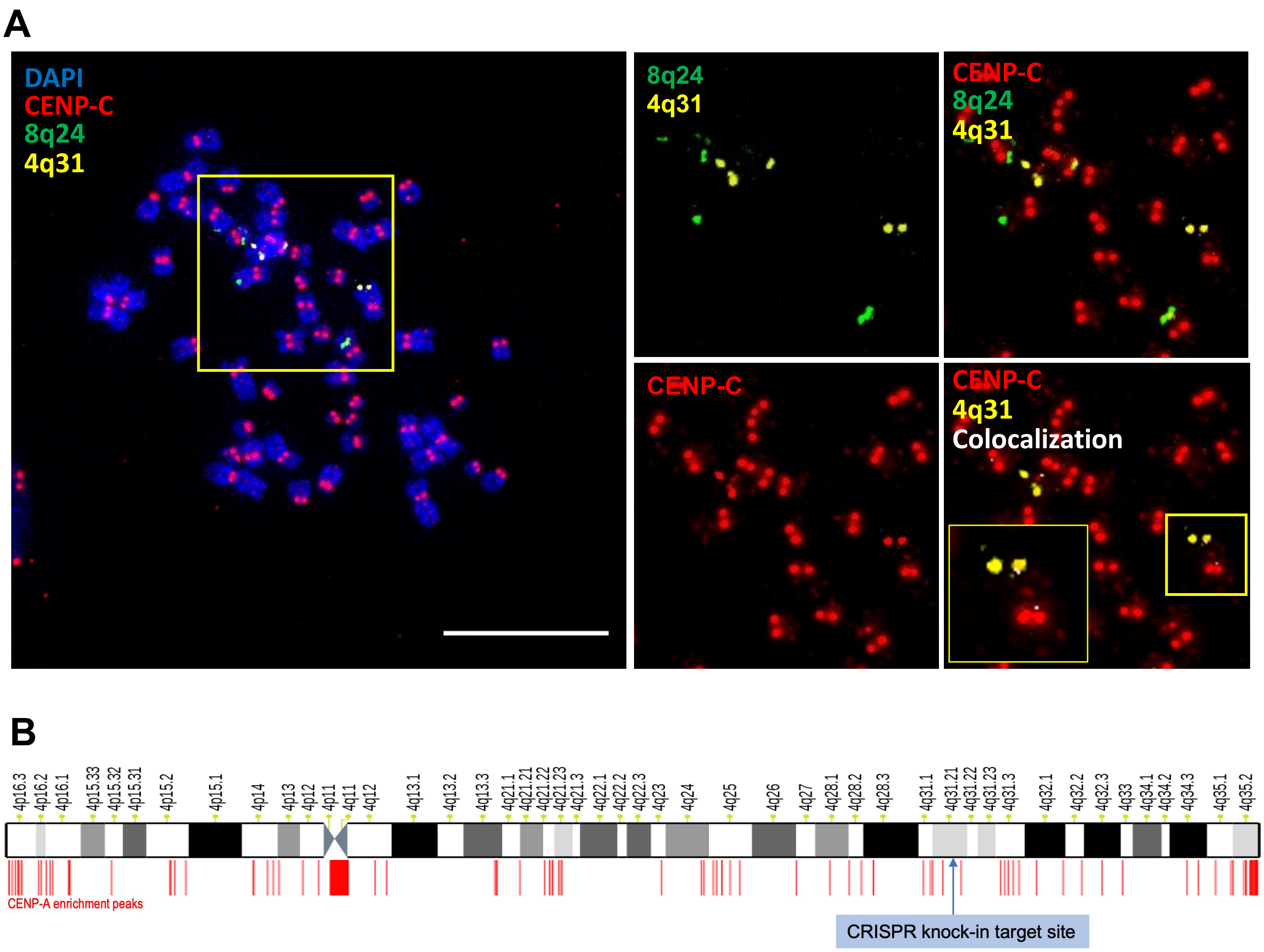

Fig. S16. The 4q31 locus probed for CENP-C in SW480 colon cancer cells. A. SW480 colon cancer cells (wild) probed for CENP-C (Red) by immunofluorescence, and the 8q24 (Green) and the 4q31 (Yellow) locus by DNA-FISH (left). The colocalization analysis of CENP-C at the 4q31 locus is shown in inset image (White, bottom right inset). The CENP-C channel (Red) in the inset figure is enhanced to visualize the signals from ectopic sites. Scale bar represents 10 µm. **B.** CENP-A enrichment peaks on chromosome 4 of $SW480^{wild}$ cells. The blue arrow indicates the CRISPR knock-in target site at the 4q31 region.

Fig. S17.

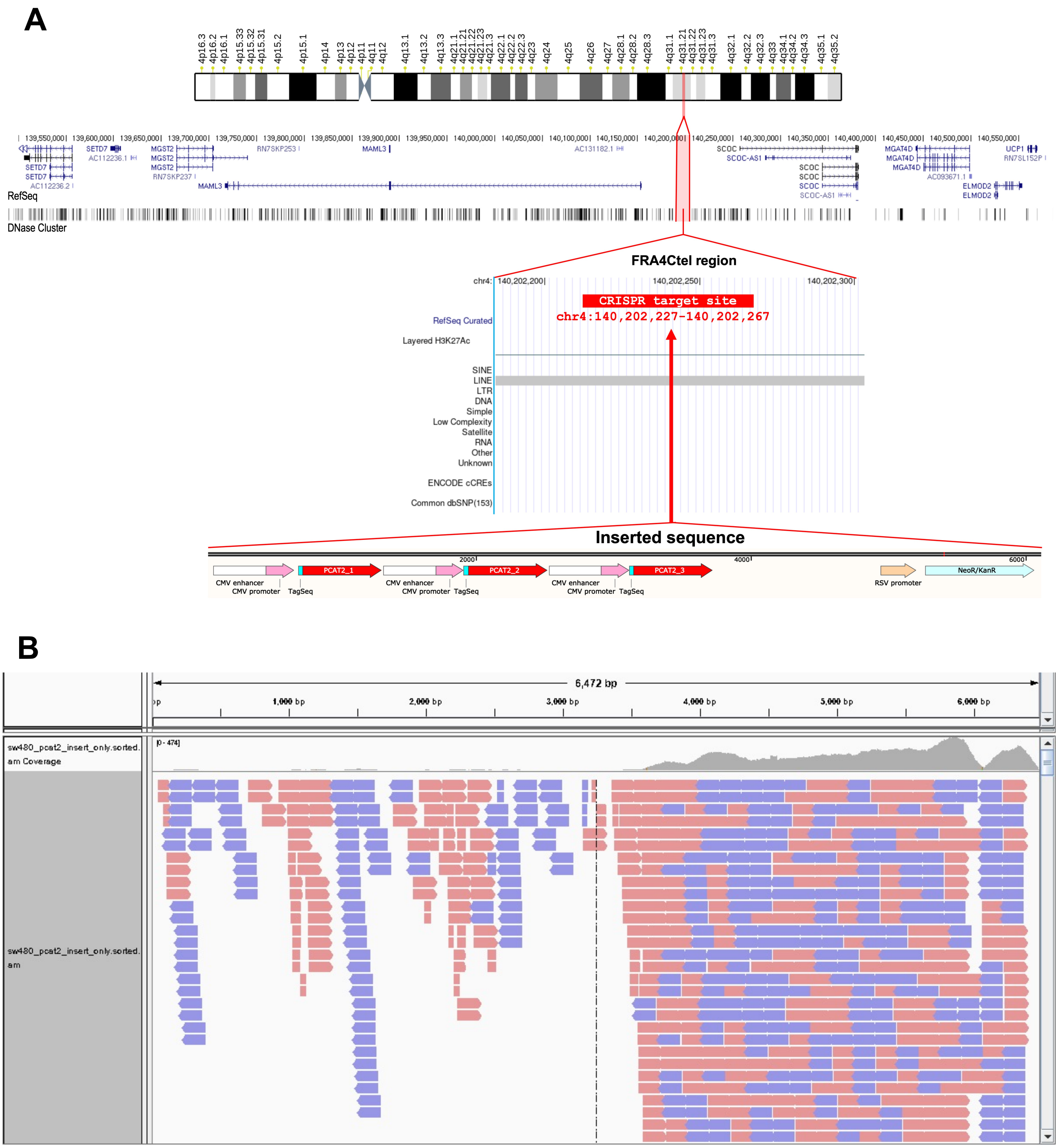

Fig. S17. CRISPR knock-in of Trans*PCAT2* array to the chromosome 4q31 locus. A. Genome browser view (schematics) of CRISPR knock-in of the transgene *PCAT2* (array) into the chromosome 4q31 locus of SW480 colon cancer cells. The 4q31 locus is a fragile subtelomeric site that has no non-coding gene or enrichment of repeat elements. This region is also not identified as an ectopic site for CENP-A in the SW480 colon cancer cells. **B.** Browser view of the Trans*PCAT2* array insert sequence from the whole genome sequencing alignment. A custom reference genome sequence of the chromosome 4 was developed to identify the CRISPR insert into the 4q31.21 region of the SW480 colon cancer cells.

Fig. S18.

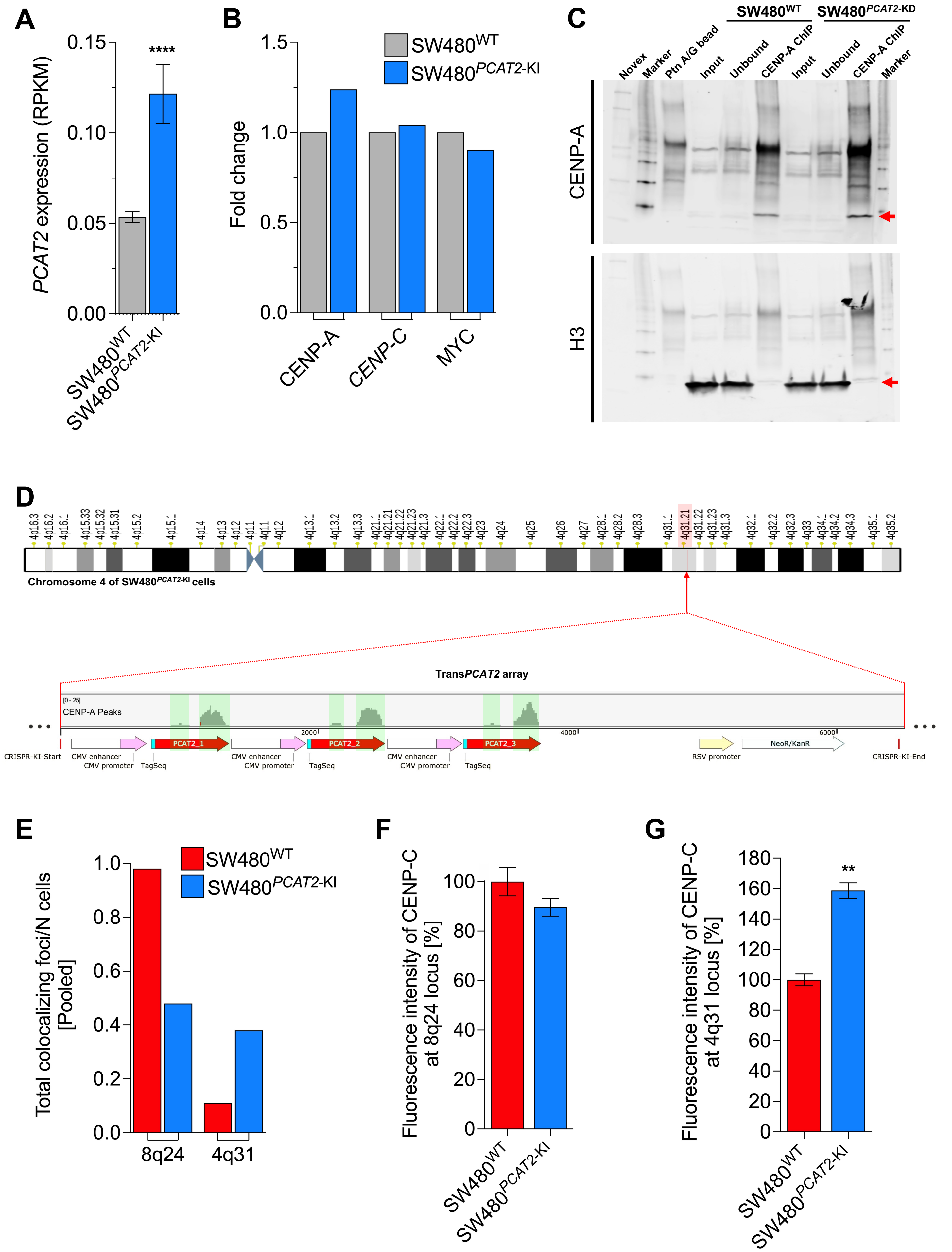
A
PCAT2 expression (RPKM)
****
SW480WT
SW480PCAT2-KI
B
Fold change
SW480WT
SW480PCAT2-KI
CENP-A
CENP-C
MYC
C
SW480WT
SW480PCAT2-KD
Novex
Marker
Ptn A/G bead
Input
Unbound
CENP-A ChIP
Marker
CENP-A
H3
D
Chromosome 4 of SW480PCAT2-KI cells
TransPCAT2 array
CENP-A Peaks
PCAT2_1
PCAT2_2
PCAT2_3
CRISPR-KI-Start
CMV enhancer
CMV promoter
TagSeq
RSV promoter
NeoR/KanR
CRISPR-KI-End
E
Total colocalizing foci/N cells [Pooled]
8q24
4q31
F
Fluorescence intensity of CENP-C at 8q24 locus [%]
G
Fluorescence intensity of CENP-C at 4q31 locus [%]
**

Fig. S18. Effect of Trans*PCAT2* RNA at the target locus in the SW480$^{PCAT2\text{-}KI}$ cells. A. *PCAT2* expression change between SW480^{WT} and SW480$^{PCAT2\text{-}KI}$ colon cancer cells represented in mean RPKM. **B.** Comparative expression levels of *CENP-A*, *CENP-C,* and *cMYC* from RNA-Seq between SW480^{WT} and SW480$^{PCAT2\text{-}KI}$ colon cancer cells in fold change. **C.** Efficiency of CENP-A ChIP was accessed by immunoblotting of SW480^{WT} and SW480$^{PCAT2\text{-}KI}$ samples probed for CENP-A and H3 proteins along with appropriate protein ladders and Protein A/G magnetic bead. **D.** CENP-A ChIP-Seq peaks in the 4q31 region (Trans*PCAT2* insert locus) of SW480$^{PCAT2\text{-}KI}$ cells. **E.** Histogram showing average (pooled average of all weeks) CENP-C colocalizing foci per cell between SW480^{WT} and SW480$^{PCAT2\text{-}KI}$ colon cancer cells (independent blinded analysis done by M.B.). The 8q24 locus lost significant amount of CENP-C while the Trans*PCAT2* locus gained a four-fold higher CENP-C in the SW480$^{PCAT2\text{-}KI}$ colon cancer cells. **F.** Histogram showing the intensity of CENP-C at 8q24 locus of SW480^{WT} and SW480$^{PCAT2\text{-}KI}$ colon cancer cells. The measurement were taken at week 3 post-stable transfection of the transgene cassette. **G.** Histogram showing the intensity of CENP-C at 4q31 locus of SW480^{WT} and SW480$^{PCAT2\text{-}KI}$ colon cancer cells (carrying TransPCAT2 cassette at its 4q31 locus). The SW480$^{PCAT2\text{-}KI}$ colon cancer cells acquired significant higher levels ($p=0.0019$) of ectopic CENP-C signal at 4q31 locus compared to the wild type cells.

Fig. S19.

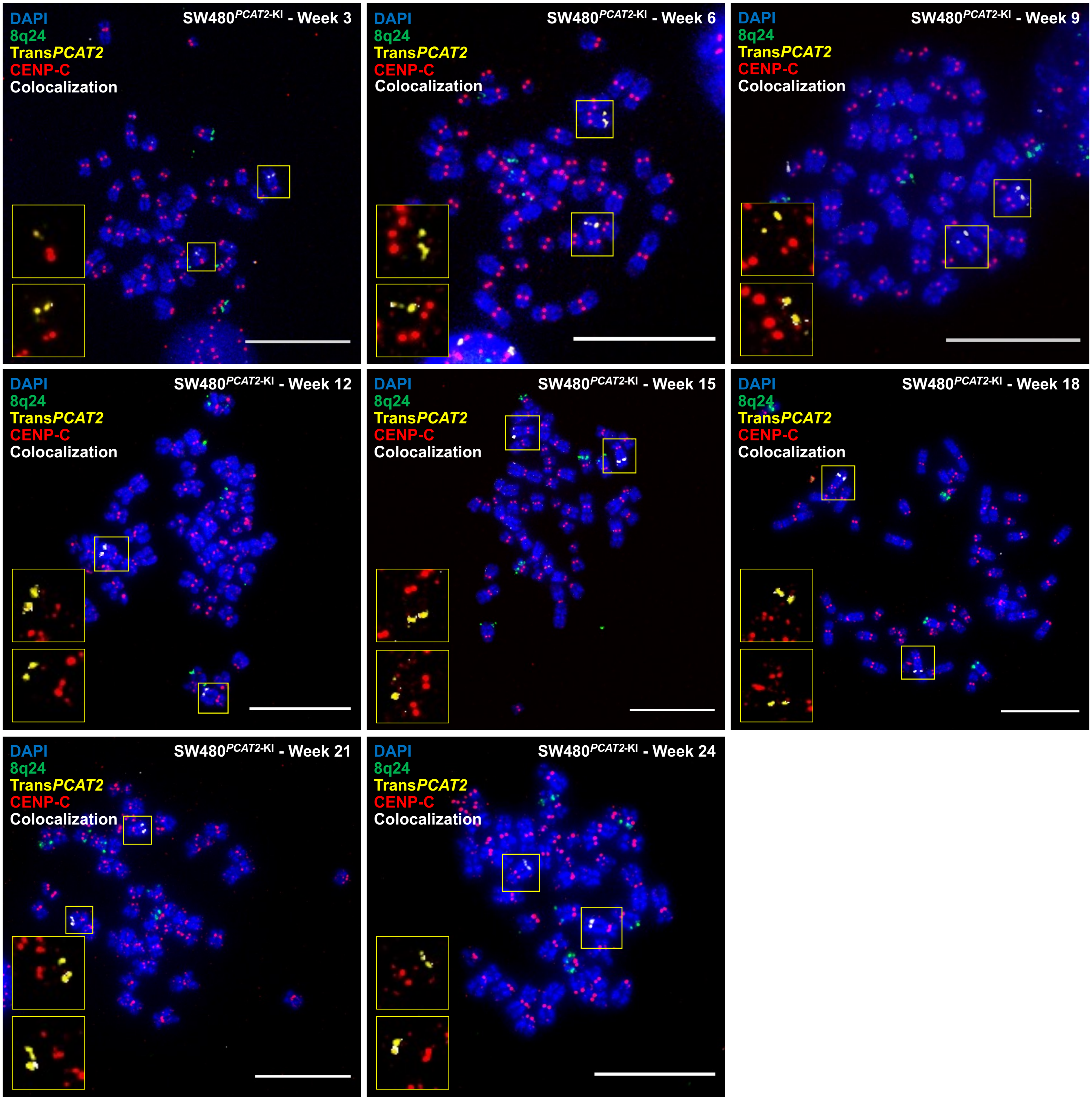

Fig. S19. Evolution of Trans*PCAT2* locus in the SW480*PCAT2*-KI cells. Representative metaphase images of SW480*PCAT2*-KI colon cancer cells (linked to Fig. 6D) probed for CENP-C by IF (Red) and 8q24/Trans*PCAT2* loci by DNA-FISH (Green/Yellow). The SW480*PCAT2*-KI cells were collected on weeks 3, 6, 9, 12, 15, 18, 21, and 24 to study the evolution of the CENP-A/CENP-C at the Trans*PCAT2* locus. The colocalization of CENP-C at the Trans*PCAT2* locus is marked in White color by the colocalization finder plugin in ImageJ. Scale bar represents 10 μm.

Fig. S20.

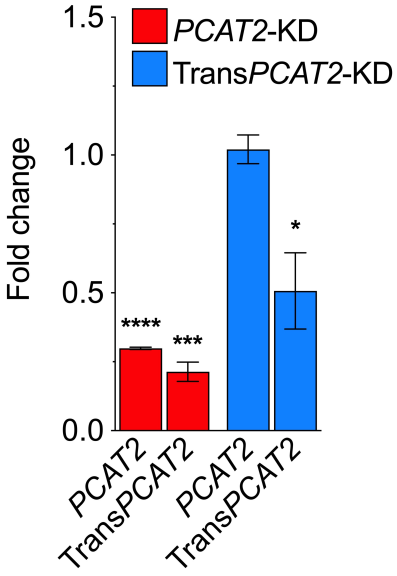

Fig. S20. *Cis*-function of Trans*PCAT2* locus in SW480$^{PCAT2\text{-}KI}$ cells. Expression fold change of *PCAT2* and Trans*PCAT2* RNA in targeted knockdown. ASO targeting shared sequence of *PCAT2* RNA reduced both *PCAT2* (p<0.0001) and Trans*PCAT2* RNA (p=0.002) significantly after 48 h. Targeting the Trans*PCAT2* RNA using ASO against the tag sequence reduced Trans*PCAT2* RNA (p=0.0372) significantly but not the native *PCAT2* RNA.

Table S1

CENP-A domains (hg19 coordinates)

S. No.	chromosome	start	end	ID
1	chr1	230849	41457972	chr1:230850:41457972
2	chr1	151408999	171379617	chr1:151409000:171379617
3	chr1	186392714	211531730	chr1:186392715:211531730
4	chr2	10001	16844793	chr2:10002:16844793
5	chr2	194116591	242942234	chr2:194116592:242942234
6	chr4	449576	29171905	chr4:449577:29171905
7	chr4	180804566	191044276	chr4:180804567:191044276
8	chr5	10001	21288933	chr5:10002:21288933
9	chr5	164779926	180905260	chr5:164779927:180905260
10	chr6	135346	16106341	chr6:135347:16106341
11	chr6	151502021	171055067	chr6:151502022:171055067
12	chr7	294513	19003321	chr7:294514:19003321
13	chr7	36185182	58054331	chr7:36185183:58054331
14	chr7	127676833	159128663	chr7:127676834:159128663
15	chr8	10001	8667842	chr8:10002:8667842
16	chr8	116821729	146304022	chr8:116821730:146304022
17	chr9	122586458	141153431	chr9:122586459:141153431
18	chr10	260877	9499130	chr10:260878:9499130
19	chr10	116320443	135395989	chr10:116320444:135395989
20	chr11	60001	10354866	chr11:60002:10354866
21	chr11	17781940	22518043	chr11:17781941:22518043
22	chr11	55309068	72797751	chr11:55309069:72797751
23	chr11	116228198	134946516	chr11:116228199:134946516
24	chr12	108118336	133841895	chr12:108118337:133841895
25	chr13	109501874	115109878	chr13:109501875:115109878
26	chr14	98589745	107289540	chr14:98589746:107289540
27	chr15	26007066	33937103	chr15:26007067:33937103
28	chr16	83841467	90294753	chr16:83841468:90294753
29	chr17	5520690	10736815	chr17:5520691:10736815
30	chr18	71857960	78017154	chr18:71857961:78017154
31	chr19	284093	19050265	chr19:284094:19050265
32	chr19	45153040	53533729	chr19:45153041:53533729
33	chr20	55066978	62965520	chr20:55066979:62965520
34	chr21	41791881	48119895	chr21:41791882:48119895
35	chr22	44169801	51244566	chr22:44169802:51244566

Table S2

CENP-B motifs and k-mer analysis of 8q24-derived lncRNAs. (Provided separately as xlsx file)

Table S3

k-mer analysis of 8q-derived lncRNA and putative centromeric transcripts. (Provided separately as xlsx file)

Table S4

LNA anti-sense oligo sequences:

Gene	ID	Sequence*	Type
MALAT1	NR_144568.1	CGTTAACTAGGCTTTA	Antisense GapmeR
PVT1	NR_003367	ATCGTAATGGGTTGAA	Antisense GapmeR
PCAT1	NR_045262	GTTAGTTGGCAAGACG	Antisense GapmeR
PCAT2	NR_119373	TCAATAAAGTTGCAGT	Antisense GapmeR
CCAT1	NR_108049	GAGGTATGCGTAGGTG	Antisense GapmeR
CCAT2	NR_109834	GGCAGTTGAGAAACGA	Antisense GapmeR
KLF6	NM_001300.6	GCAGCGGGACTTCGG	Antisense GapmeR
DAXX	NM_001141969	AGTTGGATCTCTCAG	Antisense GapmeR
HIRA	NM_003325.4	GGGAGATGACAAACTG	Antisense GapmeR
HJURP	NM_018410.5	GTGTGTAGCTAGGTTA	Antisense GapmeR
Universal Scrambled ctrl	N/A	GACTACTACGCTCGTCA	Antisense GapmeR
Trans*PCAT2*	N/A	AATAGTCGAGCGTAG	Antisense GapmeR

*all nucleotides are LNA-modified.

List of primer sequences:

Gene	Refseq	Forward	Reverse
PVT1	NR_003367	CCGACTCTTCCTGGTGAAGC	GTATGGTCAGCTCAAGCCCA
PCAT1	NR_045262	AGCTACCTATGGTGGTCTGGA	TGCTCAGGTTCCTCAGTTGG
PCAT2	NR_119373	CCCTTAAGGCACTGATGCTC	GTGCTGATGCCTCTGGAAAT
Trans*PCAT2*	N/A	TCGACTACTACGCTCGTCAGACTAC	GTGTTCCCTCCAAAATTCAGGTACT
CCAT1	NR_108049	CTGTCAACCCTGACGCTCTT	GGCAAGTTTTCCTGTGTGGC
CCAT2	NR_108049	GCTCCAGGCAATAACTGTGC	TGGGGGTAGGTCAGGAATCAGG
MALAT1	NR_144568.1	GACGGAGGTTGAGATGAAGC	TGACTCTTCCTACAGGCCCA
KLF6	NM_001300.6	CGGACGCACACAGGAGAAAA	CGGTGTGCTTTCGGAAGTG
MYC	NM_002467.6	AATGAAAAGGCCCCCAAGGTAGTTATCC	GTCGTTTCCGCAACAAGTCCTCTTC
a-Satellite	N/A	CATCACAAAGAAGTTTCTGAGAATGCTTC	TGCATTCAACTCACAGAGTTGAACCTTCC
18s rRNA	NR_146119.1	GTAACCCGTTGAACCCCATT	CCATCCAATCGGTAGTAGCG
GAPDH	NM_002046	GCGGTTCCGCACATCCCGGTAT	CCCCACGTCGCAGCTTGCCTA
DAXX	NM_001141969.2	TGCAGACACCCCCGAAGCCT	TGCCATTCCACTAGGGCCCTCA
HJURP	NM_018410.5	TCACAGCCCAACTCAGAAGA	AACAGAGAGCAAGTGGGAAGA
HIRA	NM_003325.4	GCTTCTGGGGGAGATGACAA	TTACCACTGGAGCCGAACAC

Table S5

p-values corresponding to figures in main text. The below tables contain the total number of foci counted (N) and number positive colocalization signals observed (n) for each experimental condition. The numerical data (n or % columns) presented in the below tables are presented as histograms or box plots in the corresponding main figures.

N= Total number of foci counted, n= Number of foci colocalizing CENP-A/C.

Fig. 2

A	Condition	N	n	%	p-value
Percentage CENP-A colocalization foci at 8q24 locus	Scrambled	453	340	75.1	
	PCAT1-KD	194	119	61.3	0.1549
	PCAT2-KD	235	122	51.9	0.0063
	CCAT1-KD	248	167	67.3	0.3906
	CCAT2-KD	294	177	60.2	0.0668
	PVT1-KD	276	160	58.0	0.0449

B	Condition		N	p-value
Fluorescence intensity at 8q24 [pooled]	CENP-A	Scrambled	206	
		Knockdown	843	0.0357
	CENP-A	Scrambled	212	
		Knockdown	910	0.0357

C	Condition	Time	N	n	%	p-value
Percentage CENP-A colocalization foci at 8q24 locus	*PCAT2*-KD	72h	222	113	52.3	0.0059
		96h	100	23	23.1	<0.0001
		120h	105	16	15.0	<0.0001

E	Condition	Time	N	n	%	p-value
Percentage CENP-C colocalization foci at 8q24 locus	Scrambled	72h	259	197	76.1	0.5804
		96h	420	307	73.1	0.7628
		120h	44	30	68.2	0.7053
	PCAT1-KD	72h	253	163	64.4	0.2421
		96h	347	169	48.7	0.0007
		120h	239	98	41.0	0.0708
	PCAT2-KD	72h	231	126	54.5	0.0252
		96h	315	80	25.4	0.0001
		120h	84	20	23.8	0.0023
	CCAT1-KD	72h	219	140	63.9	0.2517
		96h	288	115	39.9	0.0001
		120h	181	76	42.0	0.0896
	CCAT2-KD	72h	287	172	59.9	0.0801
		96h	330	172	52.1	0.0052
		120h	232	110	47.4	0.1765
	PVT1-KD	72h	286	174	60.8	0.1063
		96h	321	160	49.8	0.0017
		120h	246	115	46.7	0.1757

F	Time	N	p-value
Fluorescence intensity of CENP-C at 8q24 locus [pooled]	72h	910	<0.0001
	96h	1188	
	120h	924	

Fig. 3

B	Locus	N	n	%
Percentage CENP-C colocalizing foci	8q24	231	126	54.5
	2q21	41	26	63.4
	10p15	131	61	46.6

C	Locus	N	p-value
Fluorescence intensity of CENP-C	8q24	338	
	2q21	78	0.0001
	10p15	144	0.0001

F	Locus		N	n	%	p-value
Percentage CENP-C colocalizing foci [pooled]	8q24	Scrambled	420	307	73.1	0.0001
		Knockdown	1601	696	43.5	
	2p21	Scrambled	41	26	63.4	0.8977
		Knockdown	748	458	61.2	
	10p15	Scrambled	131	61	46.6	0.8661
		Knockdown	649	314	48.4	

G	Locus	Condition	N	n	%
Percentage CENP-C colocalization foci	8q24	Scrambled	420	307	73.1
		KLF6-KD	349	229	65.6
	10p15	Scrambled	131	61	46.6
		KLF6-KD	139	72	51.8

Fig. 4

B	Condition	N	n	%	p-value
Percentage CENP-C colocalization foci at 8q24 locus	Scrambled	259	197	76.1	
	PCAT2-KD	231	126	54.5	0.0252
	PCAT2-KD/nil	256	173	67.6	0.4134
	PCAT2/HIRA-KD	212	84	39.6	0.0001
	PCAT2/DAXX-KD	212	97	45.8	0.0001
	PCAT2/HIRA+DAXX-KD	119	27	22.7	0.0001

C	Condition	N	p-value
Fluorescence intensity of CENP-C at 8q24 locus	Scrambled	111	
	PCAT2-KD	114	0.0002
	PCAT2-KD/nil	54	0.4207
	PCAT2/HIRA-KD	89	0.0002
	PCAT2/DAXX-KD	94	0.0001
	PCAT2/HIRA+DAXX-KD	34	0.0001

Fig. 5

D	Cell line	Locus	Time	N	n	%	p-value	Locus	Time	N	n	%	p-value
Percentage CENP-C colocalizing foci	SW480-Wild	8q24	0	87	62	71.3		Trans*PCAT2* (4q31 locus)	0	50	5	10.0	
	SW480-*PCAT2*-KI		Week 1	382	177	46.3	0.0250		Week 1	176	37	21.0	0.1501
			Week 3	244	140	57.4	0.2756		Week 3	112	48	42.9	0.0018
			Week 6	73	50	68.5	0.7744		Week 6	25	14	56.0	0.0001
			Week 9	100	61	61.0	0.5615		Week 9	50	26	52.0	0.0008
			Week 12	130	91	70.0	1.0000		Week 12	38	22	57.9	0.0007
			Week 15	129	93	72.1	1.0000		Week 15	59	39	66.1	0.0001
			Week 18	256	192	75.0	0.8484		Week 18	102	72	70.6	0.0001
			Week 21	197	120	61.0	0.4764		Week 21	64	42	65.6	0.0001
			Week 24	146	103	70.5	1.00000		Week 24	59	44	74.6	0.0001

E	Locus	Condition	N	n	%
% CENP-C colocalizing foci	8q24	Scrambled	253	194	76.7
		PCAT2-KD	215	102	47.4
		Tag*PCAT2*-KD	222	172	77.5
	Trans*PCAT2*	Scrambled	108	89	82.4
		PCAT2-KD	98	47	48.0
		Tag*PCAT2*-KD	101	62	61.4

p-values corresponding to figures in supplement materials. The below tables contain the total number of foci counted (N) and number positive colocalization signals observed (n) for each experimental condition. The numerical data (n or % columns) presented in the below tables are presented as histograms or box plots in the corresponding supplement figures.

N= Total number of foci counted, n= Number of foci colocalizing CENP-A/C.

fig. S7

**B**	Condition	p-value		
		72h	96h	120h
RNA expression (Fold change)	*PCAT1*-KD	0.0008	0.0001	0.0001
	PCAT2-KD	0.0001	0.001	0.0001
	CCAT1-KD	0.0001	0.0377	0.0834
	CCAT2-KD	0.0001	0.01	0.1831
	PVT1-KD	0.0098	0.0001	0.0501
	MALAT1-KD	0.0001		

fig. S8

**B**	Condition	Time	N	p-value
Fluorescence intensity of CENP-C at 8q24 locus	Scrambled	72h	106	
		96h	111	
		120h	111	
	PCAT1-KD	72h	100	0.0002
		96h	127	0.0001
		120h	112	0.0001
	PCAT2-KD	72h	105	0.0001
		96h	114	0.0001
		120h	43	0.0001
	CCAT1-KD	72h	84	0.5113
		96h	120	0.0001
		120h	85	0.0001
	CCAT2-KD	72h	89	0.5139
		96h	117	0.0001
		120h	106	0.0001
	PVT1-KD	72h	77	0.0047
		96h	116	0.0001
		120h	116	0.0001

**C**	Condition	N	n	%	p-value
Percentage CENP-C colocalization foci at 8q24 locus [pooled]	Scrambled	723	534	73.9	
	PCAT1-KD	839	430	51.3	0.0001
	PCAT2-KD	630	226	35.9	0.0001
	CCAT1-KD	688	331	48.1	0.0001
	CCAT2-KD	849	454	53.5	0.0001
	PVT1-KD	853	449	52.6	0.0001

D	Condition	N	n	%	p-value
Percentage CENP-C colocalization foci at 8q24 locus	Scrambled	453	340	75.1	
	PCAT1&2-KD	171	81	47.4	0.0025
	CCAT1&2-KD	281	112	39.9	0.0001
	PCAT1&2/CCAT1&2-KD	308	113	36.7	0.0001

fig. S10

B	Condition	p-value	
		CENP-A	H3.3
Protein expression (Fold change)	*PCAT1*-KD	0.7	0.1
	PCAT2-KD	0.7	0.1
	CCAT1-KD	0.1	0.1
	CCAT2-KD	0.7	0.1
	PVT1-KD	0.7	0.1

C	Condition	p-value	
		CENP-A	H3.3
Protein expression (Fold change)	*PCAT1*-KD	0.4	0.4
	PCAT2-KD	0.4	0.4
	CCAT1-KD	0.4	0.4
	CCAT2-KD	0.4	0.4
	PVT1-KD	0.1	0.4

fig. S15

C	Locus	N	n	%	p-value
Percentage CENP-C colocalizing foci	Innate 8q24	98	68	69.4	0.1412
	Translocated 8q24	144	134	93.1	

D	Locus	N	p-value
Fluorescence intensity of CENP-C at 8q24 locus	Innate 8q24	43	0.001
	Translocated 8q24	67	

fig. S18

B	Locus	Cells	Fold change
RNA expression level	*CENP-A*	SW480^{Wild}	0.23
		SW480$^{PCAT2\text{-}KI}$	
	CENP-C	SW480^{Wild}	0.04
		SW480$^{PCAT2\text{-}KI}$	
	MYC	SW480^{Wild}	-0.09
		SW480$^{PCAT2\text{-}KI}$	

Supplement note 1.

8q24 lncRNA *PCAT2* does not directly interact with CENP-A *in vitro*.

RNA-histone interactions could either be a direct interaction, free in solution, or dependent on the chromatin fiber context, or chromatin associated processes. Therefore, we next asked whether this interaction is direct or mediated by other complexes. To assess this possibility, we turned to a classic *in vitro* biochemical experiment, the Electrophoretic Mobility Shift Assay (EMSA). Using this nucleoprotein band shift assay, we sought to test CENP-A histone variant dimers, tetramers, and full nucleosomes for their ability to complex with in vitro transcribed *PCAT2* RNA. *PCAT2* RNA was transcribed *in vitro* using the PCR amplicon of the *PCAT2* CDS flanking T7 promoter sequence on the 5`-end (fig. S21A). The quality and quantity of the resultant RNA assessed by using DEPC treated agarose and PAGE gel (fig. S21, B and C) and spectrophotometer, respectively. The quantity of the histones used in our standard salt-dialysis reconstitution assays was measured by immunoblotting, using a known quantity of BSA as a standard, and equal amounts were loaded in all the wells during EMSA. NAP1 was used to stabilize the tetramers. Reconstituted nucleosomes were assessed for quality by PAGE gel and AFM analysis (fig. S21D). About 3 µg of histones and the 400 ng of *PCAT2* RNA were mixed in 0.15 M NaCl buffer containing RNase inhibitor and incubated at room temperature for 20 min before loading into the gel. We observed no band shift in any of the wells containing CENP-A *PCAT2* RNA, suggesting a lack of direct interaction *in vitro* (fig. S21, E to G). We also performed a mild cross-linking, with 1% PFA, of CENP-A containing tetramers and nucleosomes with *PCAT2* RNA as a control for possibly labile interactions. Thus, although robust association of *PCAT2* RNA can be seen with CENP-A when immunoprecipitated from *in vivo* conditions, we did not observe a straightforward 1:1 association in vitro when reassembled with purified components.

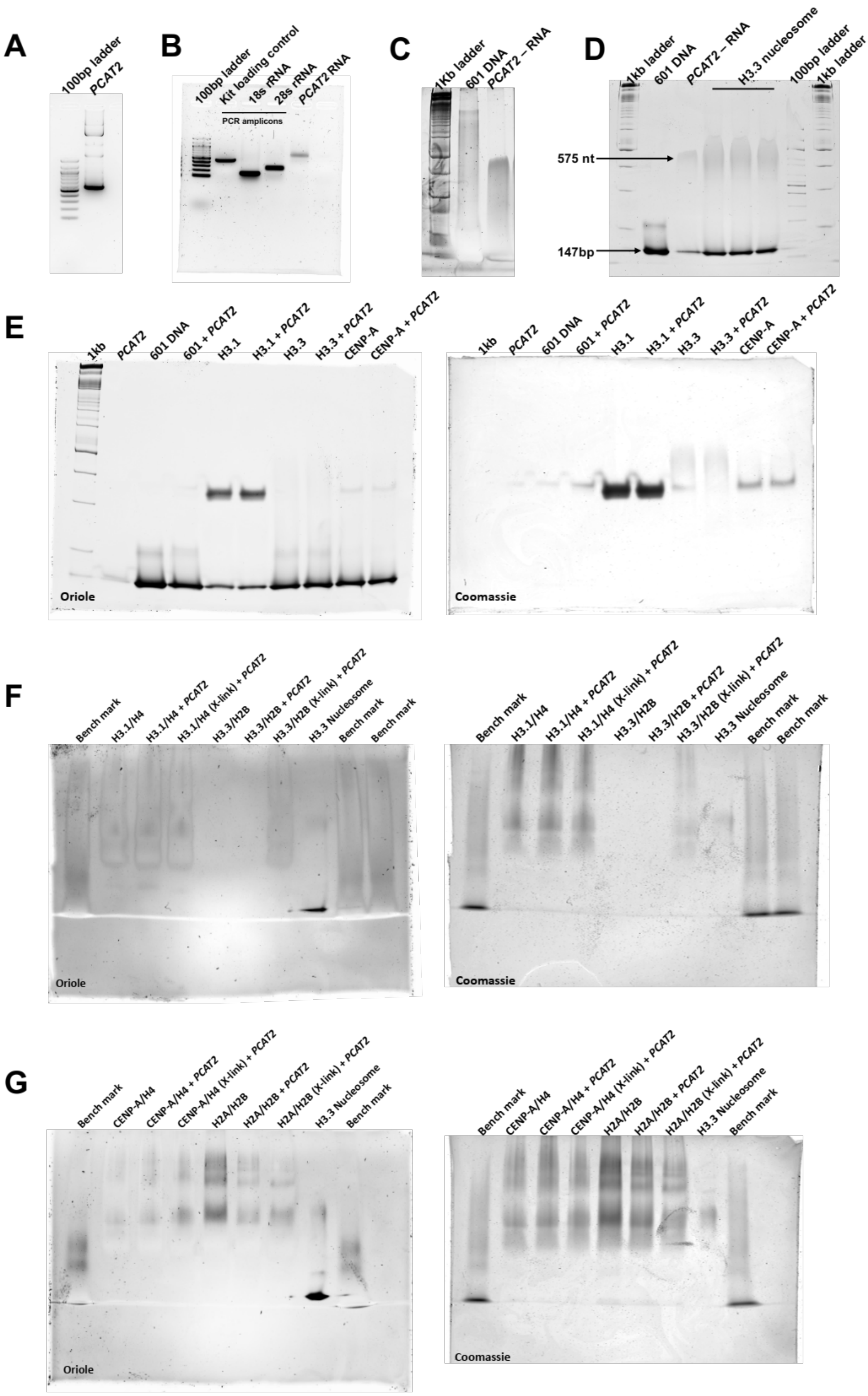
A
100bp ladder
PCAT2
B
100bp ladder
Kit loading control
18s rRNA
28s rRNA
PCAT2 RNA
PCR amplicons
C
1Kb ladder
601 DNA
PCAT2 – RNA
D
1kb ladder
601 DNA
PCAT2 – RNA
H3.3 nucleosome
100bp ladder
1kb ladder
575 nt
147bp
E
1kb
PCAT2
601 DNA
601 + PCAT2
H3.1
H3.1 + PCAT2
H3.3
H3.3 + PCAT2
CENP-A
CENP-A + PCAT2
Oriole
Coomassie
F
Bench mark
H3.1/H4
H3.1/H4 + PCAT2
H3.1/H4 (X-link) + PCAT2
H3.3/H2B
H3.3/H2B + PCAT2
H3.3/H2B (X-link) + PCAT2
H3.3 Nucleosome
Bench mark
Bench mark
Oriole
Coomassie
G
Bench mark
CENP-A/H4
CENP-A/H4 + PCAT2
CENP-A/H4 (X-link) + PCAT2
H2A/H2B
H2A/H2B + PCAT2
H2A/H2B (X-link) + PCAT2
H3.3 Nucleosome
Bench mark
Oriole
Coomassie

Fig. S21. Electrophoresis mobility shift assay of nucleosomes and histones with *PCAT2* RNA. A. PCR amplicon of *PCAT2* CDS tagged with T7-promoter sequence for *in vitro* transcription reaction. The *PCAT2* gene cloned in a pUC13 plasmid was used as a template. **B.** The *in vitro* transcribed *PCAT2* RNA resolved in DEPC treated 1% agarose gel along with the kit loading control, 18s, and 28s rRNA PCR amplicons. **C.** The *in vitro* transcribed *PCAT2* RNA was resolved in a DEPC-treated PAGE gel along with 601 DNA (147 bp) and 1Kb ladder to check the quality of the RNA for EMSA. **D.** Representative native PAGE gel loaded with 601 DNA and *PCAT2* RNA (575 bases) along with the reconstituted H3.3 nucleosome to confirm the nucleosome formation. The nucleosome formation is accessed by the band shift of the 147bp 601 DNA, present in the H3.3 nucleosome, running above the 500 bp range (marked by *PCAT2* RNA band). DNA was stained using Gel-Star DNA fluorescent intercalating dye. **E.** H3.1, H3.3, and CENP-A nucleosomes incubated with *in vitro* transcribed *PCAT2* RNA resolved in native PAGE gel stained with Oriole (left) and Coomassie (right). *PCAT2* RNA and 601 DNA severed as controls. **F.** H3.1/H4 and H3.3/H4 dimers were incubated with *in vitro* transcribed *PCAT2* RNA followed by cross-linking using 1% PFA resolved in native PAGE gel stained with Oriole (left) and Coomassie (right). H3.3 nucleosome served as control. **G.** CENP-A/H4 and H2A/H2B dimers were incubated with in vitro transcribed *PCAT2* RNA followed by cross-linking using 1% PFA resolved in native PAGE gel stained with Oriole (left) and Coomassie (right). H3.3 nucleosome served as control.

Method

Nucleosome reconstitution

Histone proteins procured from a commercial source (Epicypher, USA) were diluted in ultrapure water, loaded into 7K MWCO Slide-A-Lyzer™ Dialysis Cassettes (Cat# 66370; Thermo Scientific, USA), and dialyzed in pre-chilled 2M NaCl buffer for 2 h on a magnetic stirrer. The dialyzed samples were collected and resolved in a 6% SDS-PAGE gel (Cat# 4561023, BioRad, USA) after denaturing at 95°C. The sample band intensities were measured, and the concentration of samples was measured with respect to a known concentration of BSA as a loading control using LI-COR software (LI-COR Biosciences, USA). Histone dimers were prepared by mixing a 1:1 ratio of the histone proteins, i.e., H3 or CENP-A with H4 and H2A with H2B, and incubate overnight at

4°C. The formation of dimers was confirmed by running non-denaturing TGX Stain-Free PAGE Gels (Cat# 4568023; BioRad, USA) using 0.5X TBE buffer at 30 V in 4°C. The histone dimers H3/H4 or CENP-A/H4 were mixed with H2A/H2B dimers with 601 biotinylated DNA (Cat# 18-0005; Epicypher, USA) at a ratio of 0.9 µg histones per 1 µg DNA in 2 M NaCl, 10 mM Tris-Cl pH 8.0, 1 mM EDTA buffer. The samples were loaded into a dialysis cassette and subjected to dialysis in 1 M and 0.8 M NaCl (pre-chilled) for 2 h at room temperature followed by 0.6 M NaCl overnight at 4°C. Final dialysis was performed in 0.15 M NaCl (pre-chilled) for 2 h at room temperature. The reconstituted nucleosomes were confirmed by non-denaturing PAGE, stored at 4°C, and used within three days of preparation.

***In vitro* transcription**

In vitro transcription of lncRNA *PCAT2* was performed using SP6/T7 Transcription Kit using manufacturers' protocol (Cat# 10999644001; Roche, USA). Briefly, the T7 promoter sequence (5`-TAATACGACTCACTATAGGG-3`) was added to the forward primer and PCR amplified. The *PCAT2* gene cloned in the pUC13 plasmid served as a temple for the PCR reaction (fig. S21A). The PCR fragment was purified and used as the template for *in vitro* transcription. The *in vitro* transcribed RNA was subjected to run in DEPC treated 1% agarose gel and 6% PAGE gel to access the successful transcription reaction and stability of the RNA during electrophoresis. The synthesized RNA was stored in -20°C for downstream experiments.

Electrophoretic Mobility Shift Assay

The samples for dimer experiments were mixed with a 1:20 ratio of NAP1 for dimer formation and stabilization and subjected to mild crosslinking using EDC (Cat# 77149; Thermo Scientific, USA) or 0.1% formaldehyde in 1x PBS and incubated for 20 min at room temperature (quenched with 1 M glycine) before incubating with *PCAT2* RNA. The histone dimers and reconstituted nucleosome samples were mixed with 400 ng of *in vitro* transcribed *PCAT2* RNA in 0.15 M NaCl containing RNase inhibitor and incubated at room temperature for 20 min. The samples were gently mixed with an equal volume of non-denaturing loading dye (0.25% (w/v) xylene cyanol in 80% glycerol) and ran on TGX Stain-Free PAGE gel using 0.5X TBE buffer at 30 V in 4°C for 6 h. The gels were detached from the cassette and incubated in Oriole Fluorescent gel

stain (Cat# 1610496; Bio-Rad, USA) for 2 h or SimplyBlue™ SafeStain Coomassie dye (Cat# LC6060; Invitrogen, USA) for 1 h followed by overnight distaining before imaging in Odyssey® imager CLx (LI-COR Biosciences, USA). All the reagents and buffers used are either RNase-free or DEPC treated before the experiments.

Supplement note 2.

Transcription associated chromatin changes may impact CENP-A mislocalization

During transcription, the nascent RNA binds the template DNA strand to form RNA:DNA hybrid structures known as R-loops (*70*). Indeed, exciting new work in plants and yeast has demonstrated that in several species, centromeric transcript-associated R-loop structures drive CENP-A deposition and maintenance at native centromeres (*51,14*). As demonstrated above, the 8q24 locus is a hotspot for non-coding transcription. Therefore, we thought it plausible that transcription of this locus may possess a higher frequency of R-loops which might influence CENP-A deposition at the 8q24 locus.

To investigate this possibility, we first tested whether blocking transcription of *PCAT2* would also deplete CENP-C occupancy at 8q24 locus. To this end, we designed a targeted two guides, or six guides CRISPR-dCas9-KRAB-mediated silencing approach directed at *PCAT2* promoter (Fig. S22, A and B). This approach targets the transcriptional repressor KRAB specifically to the *PCAT2* promoter, thereby blocking the formation of a transcription bubble, which should also prevent formation of any R-loops (*77*). After 48 h of targeting, using RT-qPCR, we confirmed that *PCAT2* transcription is significantly reduced by nearly 30% and 65% for two guides and six guides, respectively (Fig. S22C). We next tested CENP-C occupancy at 8q24 under these conditions using IF-DNA-FISH. We observed that under the targeted inhibition of *PCAT2* transcription bubble, CENP-C was depleted faster relative to the ASO-mediated knockdown of the transcripts performed above (Fig. S22D).

These results suggest that 8q24-derived lncRNA transcription and its transcripts together are significant contributing factors to ectopic CENP-A localization at the 8q24 locus. These results let us to probe directly for R-loops at the 8q24 locus using the current available recombinant S9.6 antibody by IF-DNA-FISH. We observed that 57% of the 8q24 locus colocalized with S9.6 signal, of which 32% also colocalized with the CENP-C signal (Fig. S23, A to C). The specificity of the S9.6 antibody was confirmed with RNase H treatment which by destroying RNA, significantly reduced the R-loop signal (Fig. S23, B and C). Interestingly, only 45% and 23% of the R-loops were present at 10p15 and 2q21 loci, respectively (Fig. S23, D and E). Further, at the 2q21 locus merely 4% of

the R-loops colocalized with CENP-C, suggesting that the non-coding RNA gene regions produce R-loops at a higher rate.

Next, we were curious to test for the R-loop occupancy at CENP-A hotspots on a genome-wide scale, for which we used DRIP-seq data of human NTERA2 and K652 cells from a publicly available dataset (*70*). We observed a significant proportion of ectopic CENP-A site overlapping R-loops sites at gene promoter regions (41.5%) (Fig. S24A). Earlier we and others have shown that CENP-A is enriched at gene promoters (*27, 34*). Similarly, R-loops are also enriched at gene promoter and terminator regions (*70*). Moreover, the 8q24 locus has a remarkably robust R-loop signal (Fig. S24B), irrespective of the nature of the cells used for the analysis. We also observed an enrichment of γH2A.X foci at the 8q24 locus (Fig. S24C), which accumulate at R-loop induced DNA breaks (*77*). Finally, we tested the occupancy of R-loops at the Trans*PCAT2* locus and observed a significant colocalization of Trans*PCAT2* locus with R-loops (54%) (Fig. S25). Together these results suggest a possibility of an interdependent mechanism of active transcription and R-loop formation in ectopic CENP-A deposition and, thus, increase the susceptibility to DNA breaks (*51, 71*). Given limitations of current methods to probe R-loops *in vivo* rigorously (*80*), we cautiously interpret these results. Further work with new technologies will be needed to assess the precise relationship between R-loops and CENP-A deposition *in vivo.*

Results

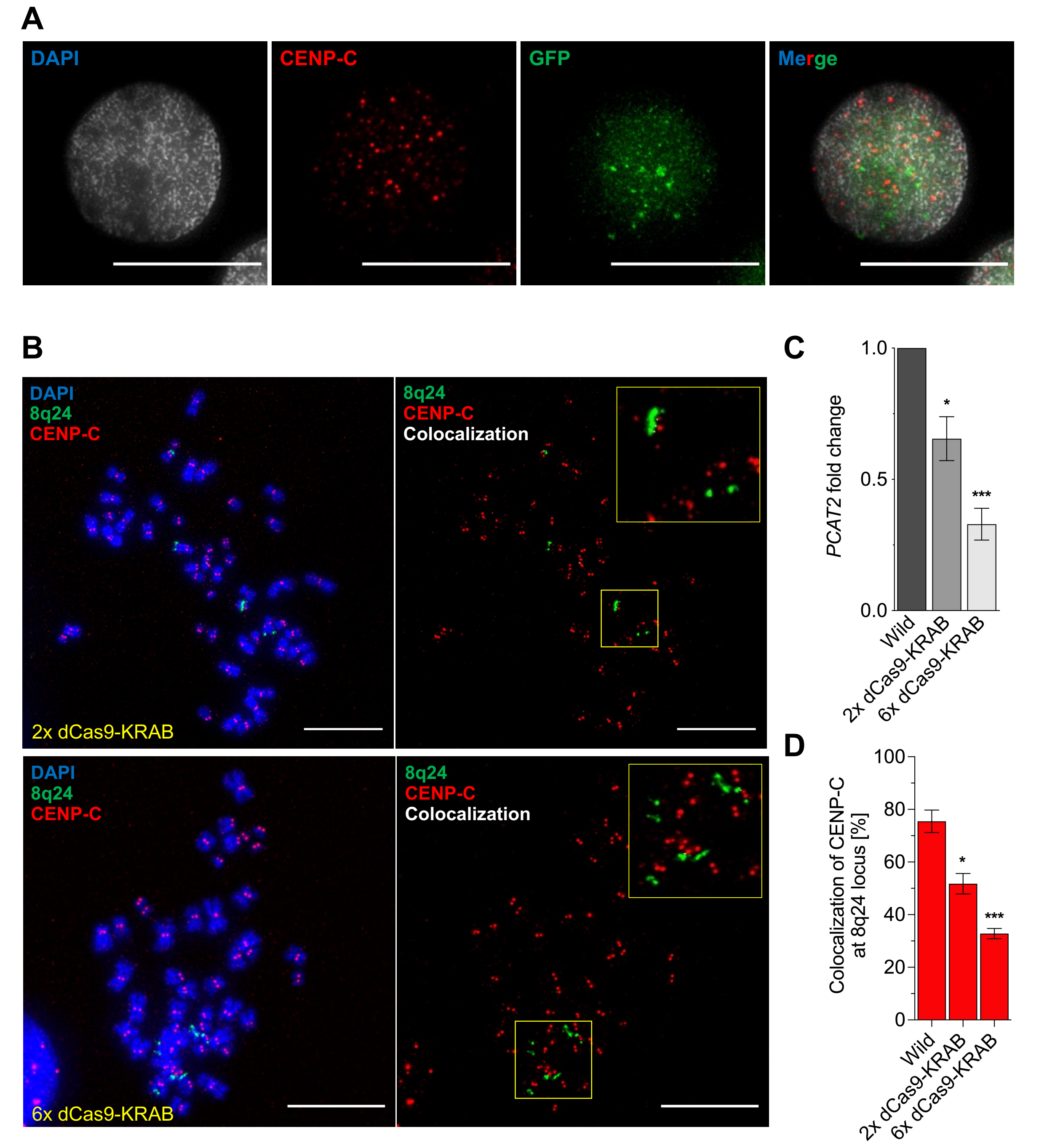

Fig. S22. CRISPR-dCas9-KRAB mediated silencing of *PCAT2* gene in SW480 cells: A. Representative image of SW480 interphase cells transfected with dCas9-KRAB plasmids carrying GFP showing the presence of GFP signal, displaying the transfection efficiency, inside the cells. Scale bar represents 10 µm. **B.** SW480 colon cancer cells treated with two (top) and six (bottom) dCas9-KRAB guides targeting *PCAT2* gene promoter, respectively. The metaphase chromosomes are probed for CENP-C (Red) by IF and the 8q24 (Green) locus by DNA-FISH. The colocalization, if any, is marked in White color. Scale bar represents 10 µm. **C.** Expression fold change of *PCAT2*

lncRNA in SW480 cells transfected with two (p=0.0146) and six dCas9-KRAB plasmids (p=0.0004) targeting different regions of the *PCAT2* gene promoter compared to wild type SW480 cells at 48 h. **D.** Percentage CENP-C colocalization foci at the 8q24 locus in SW480 cells transfected with dCas9-KRAB plasmids compared to wild type cells after 48 h.

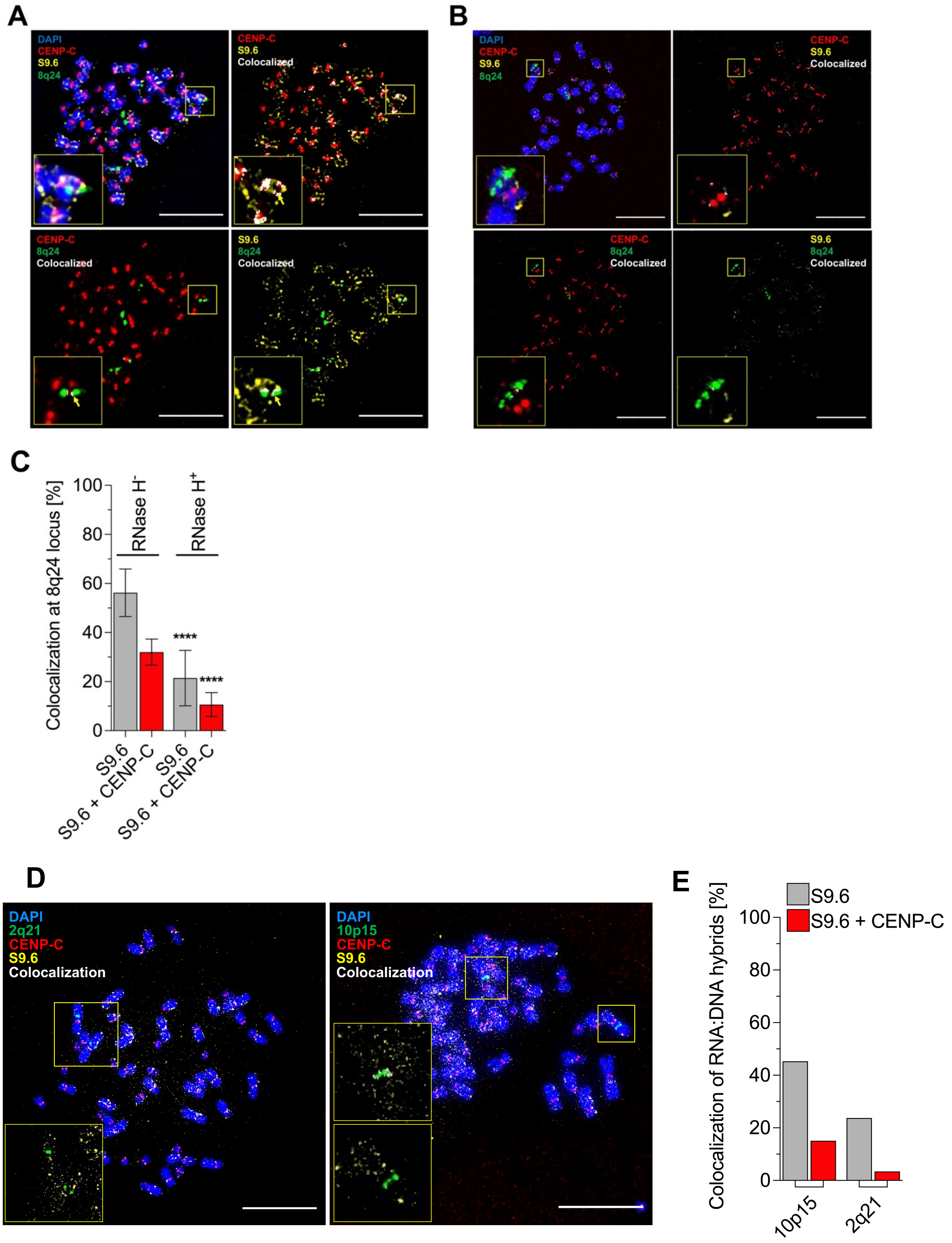

Fig. S23. R-loops occupancy correlate with CENP-C at ectopic sites. A. IF-DNA-FISH image of SW480 colon cancer cell metaphase chromosomes probed with CENP-C antibody (Red) and S9.6 antibody for RNA:DNA hybrids (Yellow). **B.** IF-DNA-FISH image of SW480 colon cancer cell

metaphase probed with CENP-C antibody (Red) and S9.6 antibody for RNA-DNA hybrids (Yellow) after RNase H treatment for 15 min. The bottom panels in **A.** and **B.** show the colocalization signals of CENP-C with S9.6 (White) at the 8q24 locus. Yellow arrows point the colocalization spots. Scale bars represent 10 µm. **C.** Percentage foci colocalizing S9.6 signal (RNA:DNA hybrids) with and without CENP-C at the 8q24 locus in untreated vs RNase H treated metaphase spread. **D.** IF-DNA-FISH image of SW480 colon cancer cell metaphase chromosomes probed with CENP-C antibody (Red) and S9.6 antibody for RNA-DNA hybrids (Yellow) at 2q21 locus (left) and 10p15 locus (right). The colocalization signals at the probed locus are shown in White color. Scale bars represent 10µm. **E.** Percentage colocalizing foci of S9.6 signal with and without CENP-C at the 2q21 and the 10p15 locus in metaphase chromosome spreads of SW480 colon cancer cells.

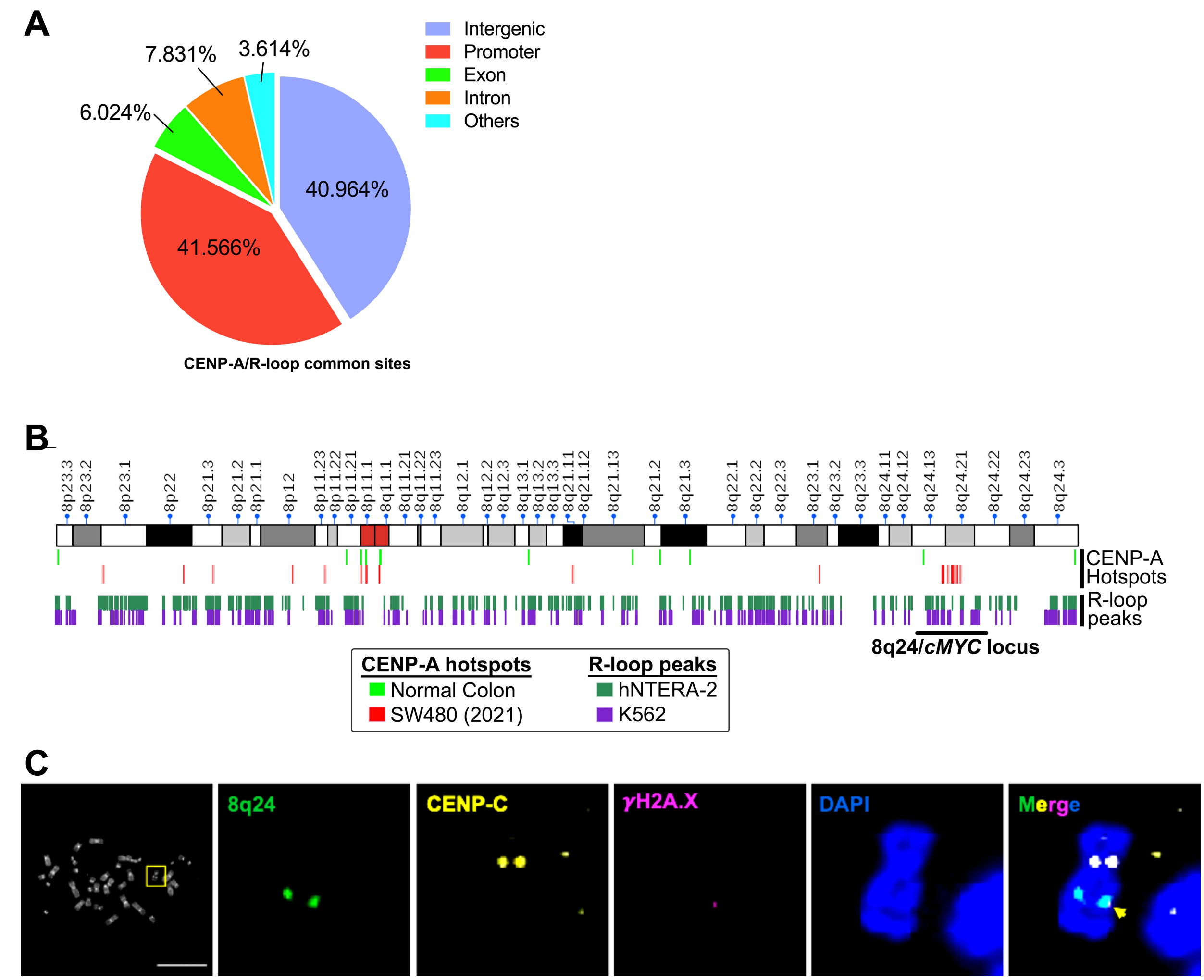

Fig. S24. R-loops co-occupancy with CENP-A and DNA damage signals at the 8q24 locus. A. Venn diagram showing number of CENP-A seq peaks coexist at R-loop sites on a genome-scale. CENP-A and R-loops colocalize at gene promoter to a remarkable level (41%). **B.** Representative karyoplot showing the CENP-A hotspots of normal colon and SW480 colon cancer cells and R-loop peaks of human NTERA-2 and K652 cells present in chromosome 8. The 8q24/cMYC region has a notable R-loop enrichment at the CENP-A sites. **C.** The IF-DNA-FISH image of SW480 cells showing the ***γ***H2A.X foci (Purple spot, Yellow arrow) at the 8q24 locus (green). CENP-C marks the centromere (Yellow). Scale bar represents 10 µm.

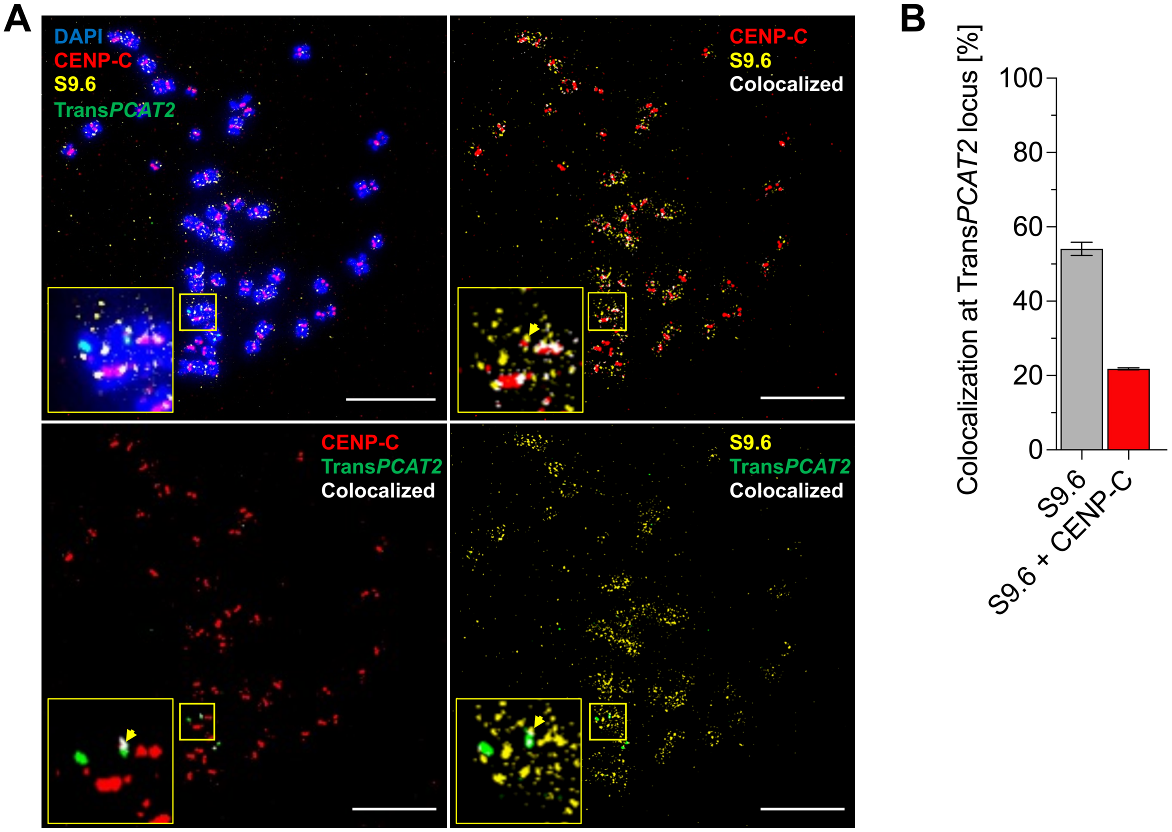

Fig S25. R-loops co-occupancy at Trans*PCAT2* locus. A. IF-DNA-FISH image of SW480$^{PCAT2\text{-KI}}$ colon cancer cell metaphase probed with CENP-C antibody (Red), and S9.6 antibody for RNA:DNA hybrids (Yellow) in the top left merged image. Experiment was performed on week 6 sample. The colocalizations of corresponding antibody signals mentioned in each image sections are shown in White color. The Yellow arrows in the inset images point to the colocalization signal of CENP-C with S9.6 at the Trans*PCAT2* locus. Scale bar represents 10 µm. **B.** Percentage foci colocalizing S9.6 signal alone and with CENP-C at Trans*PCAT2* locus in SW480$^{PCAT2\text{-KI}}$ colon cancer cells.

Methods

Protocols for metaphase preparation, IF/DNA-FISH in methanol-fixed cells and RNase H treatment are mentioned in the main text methods section. Statistical significances are derived as mentioned in the main text methods and the data for above images (Fig. S22, S23, and S25) are given below.

Tables

p-values corresponding to figures in supplement note 2. The below tables contain the total number of foci counted (N) and number positive colocalization signals observed (n) for each experimental condition. The numerical data (n or % columns) presented in the below tables are presented as histograms or box plots in Supplement note 2.

N= Total number of foci counted, n= Number of foci colocalizing CENP-C or S9.6 signal.

Fig. S22

D	Locus	Condition	N	n	%	p-value
Percentage CENP-C colocalizing foci	8q24	Wild	259	197	76.1	
		2x dCas9-KRAB	296	152	51	0.0051
		6x dCas9-KRAB	339	111	32	0.0001

Fig. S23

C	Signal	Condition	N	n	%	p-value	Signal	Condition	p-value
Percentage colocalizing foci at 8q24 locus	S9.6	Rnase H-	177	100	56.5	0.0052	S9.6	Rnase H-	0.0001
	S9.6+CENP-C	Rnase H-		57	32.2			Rnase H+	
	S9.6	Rnase H+	127	26	20.5	0.0592	S9.6+CENP-C	Rnase H-	0.0003
	S9.6+CENP-C	Rnase H+		13	10.2			Rnase H+	

E	Locus	Signal	N	n	%
Percentage colocalization	10p15	S9.6	96	29	30
		S9.6 + CENP-C		11	11
	2q21	S9.6	73	15	20
		S9.6 + CENP-C		3	4

Fig. S25

C	Locus	Signal	N	n	%
Percentage colocalizing foci	Trans*PCAT2*	S9.6	133	72	54
		S9.6 + CENP-C		29	21

Supplement file 1

***PCAT2* array:** DNA sequence of p3x-*PCAT2* plasmid array (8363bp) which contains 3 copies of lncRNA *PCAT2* gene tagged with a unique DNA sequence at its 5` end under CMV promoter. This plasmid contains Ampicillin/Neomycin/Kanamycin drug selection marker genes.

Sequence: p3-PCAT2.dna (Circular / 8363 bp)
Features: 17 total

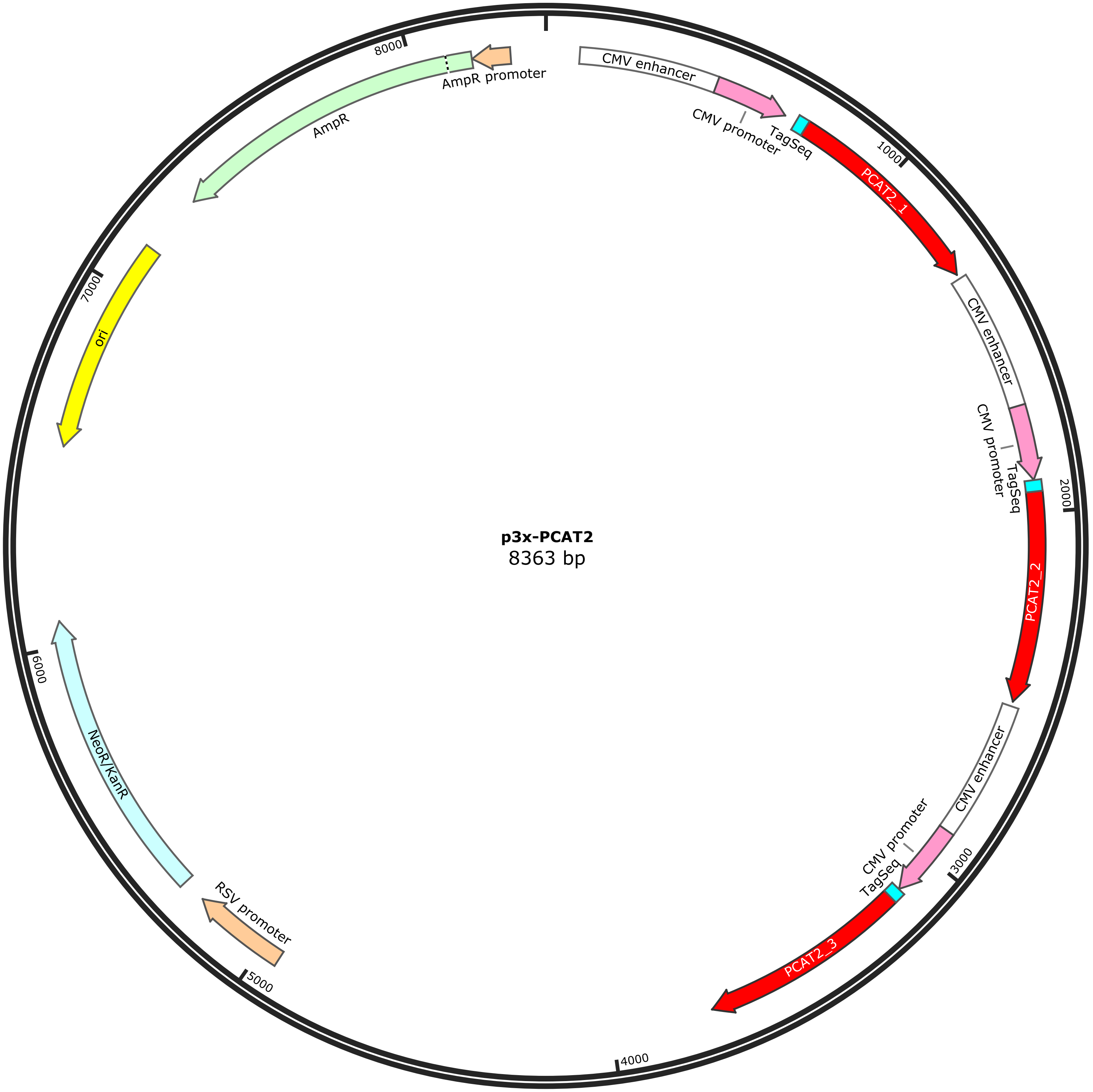

```
5′  ttcactcgaggagcttggcccattgcatacgttgtatccatatcataatatgtacatttatattg   65
3′  aagtgagctcctcgaaccgggtaacgtatgcaacataggtatagtattatacatgtaaatataac

    gctcatgtccaacattaccgccatgttgacattgattattgactagttattaatagtaatcaatt   130
    cgagtacaggttgtaatggcggtacaactgtaactaataactgatcaataattatcattagttaa
    CMV enhancer

    acggggtcattagttcatagcccatatatggagttccgcgttacataacttacggtaaatggccc   195
    tgccccagtaatcaagtatcgggtatatacctcaaggcgcaatgtattgaatgccatttaccggg
    CMV enhancer

    gcctggctgaccgcccaacgacccccgcccattgacgtcaataatgacgtatgttcccatagtaa   260
    cggaccgactggcgggttgctgggggcgggtaactgcagttattactgcatacaagggtatcatt
    CMV enhancer

    cgccaatagggactttccattgacgtcaatgggtggagtatttacggtaaactgcccacttggca   325
    gcggttatccctgaaaggtaactgcagttacccacctcataaatgccatttgacgggtgaaccgt
    CMV enhancer

    gtacatcaagtgtatcatatgccaagtacgccccctattgacgtcaatgacggtaaatggcccgc   390
    catgtagttcacatagtatacggttcatgcggggggataactgcagttactgccatttaccgggcg
    CMV enhancer

    ctggcattatgcccagtacatgaccttatgggactttcctacttggcagtacatctacgtattag   455
    gaccgtaatacgggtcatgtactggaatacccctgaaaggatgaaccgtcatgtagatgcataatc
    CMV enhancer

    tcatcgctattaccatggtgatgcggttttggcagtacatcaatgggcgtggatagcggtttgac   520
    agtagcgataatggtaccactacgccaaaaccgtcatgtagttacccgcacctatcgccaaactg
    CMV enhancer | CMV promoter >

    tcacggggatttccaagtctccaccccattgacgtcaatgggagtttgttttggcaccaaaatca   585
    agtgcccctaaaggttcagaggtggggtaactgcagttaccctcaaacaaaaccgtggttttagt
    CMV promoter >

    acgggactttccaaaatgtcgtaacaactccgccccattgacgcaaatgggcggtaggcgtgtac   650
    tgccctgaaaggttttacagcattgttgaggcggggtaactgcgtttacccgccatccgcacatg
    CMV promoter >

    ggtgggaggtctatataagcagagctcgtttagtgaaccgtcagatCGCCTgGAGACGCGACTAC   715
    ccaccctccagatatattcgtctcgagcaaatcacttggcagtctaGCGGAcCTCTGCGCTGATG
    CMV promoter >                                          TagSeq

    TACGCTCGTCAGACTACTACGCTCGACTATTTTGTGTAATACCATCATATCGGCTGCTGTTAACT   780
    ATGCGAGCAGTCTGATGATGCGAGCTGATAAAACACATTATGGTAGTATAGCCGACGACAATTGA
    TagSeq | PCAT2_1 >
```

```
ATCAGTACCTGAATTTTGGAGGGAACACATTCAAACTATAGCATTTATTTAGGCTAAAAAGATAG   845
TAGTCATGGACTTAAAACCTCCCTTGTGTAAGTTTGATATCGTAAATAAATCCGATTTTTCTATC
PCAT2_1 >

TTCATTGTCAGTGTATGAAAAACCTACTGATTTTTTACGTTGATTTTATATACTGCAACTTTATT   910
AAGTAACAGTCACATACTTTTTGGATGACTAAAAAATGCAACTAAAATATATGACGTTGAAATAA
PCAT2_1 >

GAATTCTCAACAAATGGTGTTGAAAAAACTGGATGTCCTCATGAAGAAGAATGAAATTGAACACA   975
CTTAAGAGTTGTTTACCACAACTTTTTTGACCTACAGGAGTACTTCTTCTTACTTTAACTTGTGT
PCAT2_1 >

TCACATCCCATATACAAGAATCAACTCAAAATATTTCCAAATTCAAACATTATGAAGTTTGAGCT   1040
AGTGTAGGGTATATGTTCTTAGTTGAGTTTTATAAAGGTTTAAGTTTGTAATACTTCAAACTCGA
PCAT2_1 >

ATTTAGGAAGTTAAGGAGATAATCAGATCATTTCGTCAGACACCCTTAAGGCACTGATGCTCTCA   1105
TAAATCCTTCAATTCCTCTATTAGTCTAGTAAAGCAGTCTGTGGGAATTCCGTGACTACGAGAGT
PCAT2_1 >

GTTGATCTAGCAAATAGGCATCTGTCACAGAAAATAGAGAAGATATCCCAAGAGAAGAGGAGAAA   1170
CAACTAGATCGTTTATCCGTAGACAGTGTCTTTTATCTCTTCTATAGGGTTCTCTTCTCCTCTTT
PCAT2_1 >

CTCCACAATGTGAAGAGATTTCCAGAGGCATCAGCACTCCGTCTGTCACTATTGGCATCCTGTGA   1235
GAGGTGTTACACTTCTCTAAAGGTCTCCGTAGTCGTGAGGCAGACAGTGATAACCGTAGGACACT
PCAT2_1 >

CATGGTTTAGTTTATGCGTGCTGGTTAGGCTGATTTACATAATACATTATCTAGTTATACCTTAA   1300
GTACCAAATCAAATACGCACGACCAATCCGACTAAATGTATTATGTAATAGATCAATATGGAATT
PCAT2_1 >

ATAAACCTCAAAACTGTTGGGATCCGTTGACATTGATTATTGACTAGTTATTAATAGTAATCAAT   1365
TATTTGGAGTTTTGACAACCCTAGGCAACTGTAACTAATAACTGATCAATAATTATCATTAGTTA
PCAT2_1 >          CMV enhancer

TACGGGGTCATTAGTTCATAGCCCATATATGGAGTTCCGCGTTACATAACTTACGGTAAATGGCC   1430
ATGCCCCAGTAATCAAGTATCGGGTATATACCTCAAGGCGCAATGTATTGAATGCCATTTACCGG
CMV enhancer

CGCCTGGCTGACCGCCCAACGACCCCCGCCCATTGACGTCAATAATGACGTATGTTCCCATAGTA   1495
GCGGACCGACTGGCGGGTTGCTGGGGGCGGGTAACTGCAGTTATTACTGCATACAAGGGTATCAT
CMV enhancer
```

ACGCCAATAGGGACTTTCCATTGACGTCAATGGGTGGAGTATTTACGGTAAACTGCCCACTTGGC 1560
TGCGGTTATCCCTGAAAGGTAACTGCAGTTACCCACCTCATAAATGCCATTTGACGGGTGAACCG
CMV enhancer

AGTACATCAAGTGTATCATATGCCAAGTACGCCCCCTATTGACGTCAATGACGGTAAATGGCCCG 1625
TCATGTAGTTCACATAGTATACGGTTCATGCGGGGGATAACTGCAGTTACTGCCATTTACCGGGC
CMV enhancer

CCTGGCATTATGCCCAGTACATGACCTTATGGGACTTTCCTACTTGGCAGTACATCTACGTATTA 1690
GGACCGTAATACGGGTCATGTACTGGAATACCCTGAAAGGATGAACCGTCATGTAGATGCATAAT
CMV enhancer

GTCATCGCTATTACCATGGTGATGCGGTTTTGGCAGTACATCAATGGGCGTGGATAGCGGTTTGA 1755
CAGTAGCGATAATGGTACCACTACGCCAAAACCGTCATGTAGTTACCCGCACCTATCGCCAAACT
CMV enhancer | CMV promoter

CTCACGGGGATTTCCAAGTCTCCACCCCATTGACGTCAATGGGAGTTTGTTTTGGCACCAAAATC 1820
GAGTGCCCCTAAAGGTTCAGAGGTGGGGTAACTGCAGTTACCCTCAAACAAAACCGTGGTTTTAG
CMV promoter

AACGGGACTTTCCAAAATGTCGTAACAACTCCGCCCCATTGACGCAAATGGGCGGTAGGCGTGTA 1885
TTGCCCTGAAAGGTTTTACAGCATTGTTGAGGCGGGGTAACTGCGTTTACCCGCCATCCGCACAT
CMV promoter

CGGTGGGAGGTCTATATAAGCAGAGCTCGACTACTACGCTCGTCAGACTACTACGCTCGACTATT 1950
GCCACCCTCCAGATATATTCGTCTCGAGCTGATGATGCGAGCAGTCTGATGATGCGAGCTGATAA
CMV promoter | TagSeq | PCAT2_2

TTGTGTAATACCATCATATCGGCTGCTGTTAACTATCAGTACCTGAATTTTGGAGGGAACACATT 2015
AACACATTATGGTAGTATAGCCGACGACAATTGATAGTCATGGACTTAAAACCTCCCTTGTGTAA
PCAT2_2

CAAACTATAGCATTTATTTAGGCTAAAAAGATAGTTCATTGTCAGTGTATGAAAAACCTACTGAT 2080
GTTTGATATCGTAAATAAATCCGATTTTTCTATCAAGTAACAGTCACATACTTTTTGGATGACTA
PCAT2_2

TTTTTACGTTGATTTTATATACTGCAACTTTATTGAATTCTCAACAAATGGTGTTGAAAAAACTG 2145
AAAAATGCAACTAAAATATATGACGTTGAAATAACTTAAGAGTTGTTTACCACAACTTTTTTGAC
PCAT2_2

GATGTCCTCATGAAGAAGAATGAAATTGAACACATCACATCCCATATACAAGAATCAACTCAAAA 2210
CTACAGGAGTACTTCTTCTTACTTTAACTTGTGTAGTGTAGGGTATATGTTCTTAGTTGAGTTTT
PCAT2_2

TATTTCCAAATTCAAACATTATGAAGTTTGAGCTATTTAGGAAGTTAAGGAGATAATCAGATCAT 2275
ATAAAGGTTTAAGTTTGTAATACTTCAAACTCGATAAATCCTTCAATTCCTCTATTAGTCTAGTA
PCAT2_2

TTCGTCAGACACCCTTAAGGCACTGATGCTCTCAGTTGATCTAGCAAATAGGCATCTGTCACAGA 2340
AAGCAGTCTGTGGGAATTCCGTGACTACGAGAGTCAACTAGATCGTTTATCCGTAGACAGTGTCT
PCAT2_2

AAATAGAGAAGATATCCCAAGAGAAGAGGAGAAACTCCACAATGTGAAGAGATTTCCAGAGGCAT 2405
TTTATCTCTTCTATAGGGTTCTCTTCTCCTCTTTGAGGTGTTACACTTCTCTAAAGGTCTCCGTA
PCAT2_2

CAGCACTCCGTCTGTCACTATTGGCATCCTGTGACATGGTTTAGTTTATGCGTGCTGGTTAGGCT 2470
GTCGTGAGGCAGACAGTGATAACCGTAGGACACTGTACCAAATCAAATACGCACGACCAATCCGA
PCAT2_2

GATTTACATAATACATTATCTAGTTATACCTTAAATAAACCTCAAAACTAGTCAAGCTTGTTGAC 2535
CTAAATGTATTATGTAATAGATCAATATGGAATTTATTTGGAGTTTTGATCAGTTCGAACAACTG
PCAT2_2
CMV enhancer

ATTGATTATTGACTAGTTATTAATAGTAATCAATTACGGGGTCATTAGTTCATAGCCCATATATG 2600
TAACTAATAACTGATCAATAATTATCATTAGTTAATGCCCCAGTAATCAAGTATCGGGTATATAC
CMV enhancer

GAGTTCCGCGTTACATAACTTACGGTAAATGGCCCGCCTGGCTGACCGCCCAACGACCCCCGCCC 2665
CTCAAGGCGCAATGTATTGAATGCCATTTACCGGGCGGACCGACTGGCGGGTTGCTGGGGGCGGG
CMV enhancer

ATTGACGTCAATAATGACGTATGTTCCCATAGTAACGCCAATAGGGACTTTCCATTGACGTCAAT 2730
TAACTGCAGTTATTACTGCATACAAGGGTATCATTGCGGTTATCCCTGAAAGGTAACTGCAGTTA
CMV enhancer

GGGTGGAGTATTTACGGTAAACTGCCCACTTGGCAGTACATCAAGTGTATCATATGCCAAGTACG 2795
CCCACCTCATAAATGCCATTTGACGGGTGAACCGTCATGTAGTTCACATAGTATACGGTTCATGC
CMV enhancer

CCCCCTATTGACGTCAATGACGGTAAATGGCCCGCCTGGCATTATGCCCAGTACATGACCTTATG 2860
GGGGGATAACTGCAGTTACTGCCATTTACCGGGCGGACCGTAATACGGGTCATGTACTGGAATAC
CMV enhancer

GGACTTTCCTACTTGGCAGTACATCTACGTATTAGTCATCGCTATTACCATGGTGATGCGGTTTT 2925
CCTGAAAGGATGAACCGTCATGTAGATGCATAATCAGTAGCGATAATGGTACCACTACGCCAAAA
CMV enhancer
CMV promoter

GGCAGTACATCAATGGGCGTGGATAGCGGTTTGACTCACGGGGATTTCCAAGTCTCCACCCCATT 2990
CCGTCATGTAGTTACCCGCACCTATCGCCAAACTGAGTGCCCCTAAAGGTTCAGAGGTGGGGTAA
CMV promoter

GACGTCAATGGGAGTTTGTTTTGGCACCAAAATCAACGGGACTTTCCAAAATGTCGTAACAACTC 3055
CTGCAGTTACCCTCAAACAAAACCGTGGTTTTAGTTGCCCTGAAAGGTTTTACAGCATTGTTGAG
CMV promoter

CGCCCCATTGACGCAAATGGGCGGTAGGCGTGTACGGTGGGAGGTCTATATAAGCAGAGCTCGAC 3120
GCGGGGTAACTGCGTTTACCCGCCATCCGCACATGCCACCCTCCAGATATATTCGTCTCGAGCTG
CMV promoter
TagSeq

TACTACGCTCGTCAGACTACTACGCTCGACTATTTTGTGTAATACCATCATATCGGCTGCTGTTA 3185
ATGATGCGAGCAGTCTGATGATGCGAGCTGATAAAACACATTATGGTAGTATAGCCGACGACAAT
TagSeq
PCAT2_3

ACTATCAGTACCTGAATTTTGGAGGGAACACATTCAAACTATAGCATTTATTTAGGCTAAAAAGA 3250
TGATAGTCATGGACTTAAAACCTCCCTTGTGTAAGTTTGATATCGTAAATAAATCCGATTTTTCT
PCAT2_3

TAGTTCATTGTCAGTGTATGAAAAACCTACTGATTTTTTACGTTGATTTTATATACTGCAACTTT 3315
ATCAAGTAACAGTCACATACTTTTTGGATGACTAAAAAATGCAACTAAAATATATGACGTTGAAA
PCAT2_3

ATTGAATTCTCAACAAATGGTGTTGAAAAAACTGGATGTCCTCATGAAGAAGAATGAAATTGAAC 3380
TAACTTAAGAGTTGTTTACCACAACTTTTTTGACCTACAGGAGTACTTCTTCTTACTTTAACTTG
PCAT2_3

ACATCACATCCCATATACAAGAATCAACTCAAAATATTTCCAAATTCAAACATTATGAAGTTTGA 3445
TGTAGTGTAGGGTATATGTTCTTAGTTGAGTTTTATAAAGGTTTAAGTTTGTAATACTTCAAACT
PCAT2_3

GCTATTTAGGAAGTTAAGGAGATAATCAGATCATTTCGTCAGACACCCTTAAGGCACTGATGCTC 3510
CGATAAATCCTTCAATTCCTCTATTAGTCTAGTAAAGCAGTCTGTGGGAATTCCGTGACTACGAG
PCAT2_3

TCAGTTGATCTAGCAAATAGGCATCTGTCACAGAAAATAGAGAAGATATCCCAAGAGAAGAGGAG 3575
AGTCAACTAGATCGTTTATCCGTAGACAGTGTCTTTTATCTCTTCTATAGGGTTCTCTTCTCCTC
PCAT2_3

AAACTCCACAATGTGAAGAGATTTCCAGAGGCATCAGCACTCCGTCTGTCACTATTGGCATCCTG 3640
TTTGAGGTGTTACACTTCTCTAAAGGTCTCCGTAGTCGTGAGGCAGACAGTGATAACCGTAGGAC
PCAT2_3

```
TGACATGGTTTAGTTTATGCGTGCTGGTTAGGCTGATTTACATAATACATTATCTAGTTATACCT   3705
ACTGTACCAAATCAAATACGCACGACCAATCCGACTAAATGTATTATGTAATAGATCAATATGGA
PCAT2_3

TAAATAAACCTCAAAACTTATGtctagattagataaaagtaaagtgattaacagcgcattagagc   3770
ATTTATTTGGAGTTTTGAATACagatctaatctattttcatttcactaattgtcgcgtaatctcg
PCAT2_3

tgcttaatgaggtcggaatcgaaggtttaacaacccgtaaactcgcccagaagctaggtgtagag   3835
acgaattactccagccttagcttccaaattgttgggcatttgagcgggtcttcgatccacatctc

cagcctacattgtattggcacgtgcgcaacaagcagactcttatgaacatgctttcagaggcaat   3900
gtcggatgtaacataaccgtgcacgcgttgttcgtctgagaatacttgtacgaaagtctccgtta

actggcgaagcatcacacccgttcagcaccgttaccgactgagagttggcagcagtttctccagg   3965
tgaccgcttcgtagtgtgggcaagtcgtggcaatggctgactctcaaccgtcgtcaaagaggtcc

aaaatgctctgagtttccgtaaagcattactggtccatcgtgatggagcccgattgcatataggg   4030
ttttacgagactcaaaggcatttcgtaatgaccaggtagcactacctcgggctaacgtatatccc

acctctcctacgcccccccagtttgaacaagcagaggcgcaactacgctgtctatgcgatgcagg   4095
tggagaggatgcgggggggtcaaacttgttcgtctccgcgttgatgcgacagatacgctacgtcc

gttttcggtcgaggaggctcttttcattctgcaatctatcagccattttacgttgggtgcagtat   4160
caaaagccagctcctccgagaaaagtaagacgttagatagtcggtaaaatgcaacccacgtcata

tagaggagcaagcaacaaaccagatagaaaataatcatgtgatagacgctgcaccaccattatta   4225
atctcctcgttcgttgtttggtctatcttttattagtacactatctgcgacgtggtggtaataat

caagaggcatttaatattcaggcgagaacctctgctgaaatggccttccatttcgggctgaaatc   4290
gttctccgtaaattataagtccgctcttggagacgactttaccggaaggtaaagcccgactttag

attaatatttggattttctgcacagttagatgaaaaaaagcatacacccattgaggatggtaata   4355
taattataaacctaaaagacgtgtcaatctactttttttcgtatgtgggtaactcctaccattat

aaccaaaaaagaagagaaagctagcagtgtcagtgacatttgaagatgtggctgtgctctttact   4420
ttggttttttcttctctttcgatcgtcacagtcactgtaaacttctacaccgacacgagaaatga

cgggacgagtggaagaagctggatctgtctcagagaagcctgtaccgtgaggtgatgctggagaa   4485
gccctgctcaccttcttcgacctagacagagtctcttcggacatggcactccactacgacctctt

ttacagcaacctggcctccatggcaggattcctgtttaccaaaccaaaggtgatctccctgttgc   4550
aatgtcgttggaccggaggtaccgtcctaaggacaaatggtttggtttccactagagggacaacg

agcaaggagaggatccctggtaaatcgattaactaagtaaggatccagacatgataagatacatt   4615
tcgttcctctcctagggaccatttagctaattgattcattcctaggtctgtactattctatgtaa
```

```
gatgagtttggacaaaccacaactagaatgcagtgaaaaaaatgctttatttgtgaaatttgtga  4680
ctactcaaacctgtttggtgttgatcttacgtcactttttttacgaaataaacactttaaacact

tgctattgctttatttgtaaccattataagctgcaataaacaagttaacaacaacaattgcattc  4745
acgataacgaaataaacattggtaatattcgacgttatttgttcaattgttgttgttaacgtaag

atttatgtttcaggttcagggggaggtgtgggaggttttttaaagcaagtaaaacctctacaaa  4810
taaaatacaaagtccaagtccccctccacaccctccaaaaaatttcgttcattttggagatgttt

tgtggtatggctgattatgatcctgcaagcctcgtcgtcctggccggaccacgctatctgtgcaa  4875
acaccataccgactaatactaggacgttcggagcagcaggaccggcctggtgcgatagacacgtt

ggtccccggccccggacgcgcgctccatgagcagagcgcccgccgccgaggcgaagactcgggCG  4940
ccaggggccggggcctgcgcgcgaggtactcgtctcgcgggcggcggctccgcttctgagcccGC

GCGCCAATGTAGTCTTATGCAATACACTTGTAGTCTTGCAACATGGTAACGATGAGTTAGCAACA  5005
CGCGGTTACATCAGAATACGTTATGTGAACATCAGAACGTTGTACCATTGCTACTCAATCGTTGT
RSV promoter

TGCCTTACAAGGAGAGAAAAAGCACCGTGCATGCCGATTGGTGGAAGTAAGGTGGTACGATCGTG  5070
ACGGAATGTTCCTCTCTTTTTCGTGGCACGTACGGCTAACCACCTTCATTCCACCATGCTAGCAC
RSV promoter

CCTTATTAGGAAGGCAACAGACAGGTCTGACATGGATTGGACGAACCACTGAATTGCGCATTGCA  5135
GGAATAATCCTTCCGTTGTCTGTCCAGACTGTACCTAACCTGCTTGGTGACTTAACGCGTAACGT
RSV promoter

GAGATAATTGTATTTAAGTGCCTAGCTCGATACAATAAACGCCATTTGACCATTCACCACATTGG  5200
CTCTATTAACATAAATTCACGGATCGAGCTATGTTATTTGCGGTAAACTGGTAAGTGGTGTAACC
RSV promoter

TGTGCACCtaggcttaggcttttgcaaagatcgatcaagagacaggatgaggatcgtttcgccat  5265
ACACGTGGatccgaatccgaaaacgtttctagctagttctctgtcctactcctagcaaagcggta
RSV promoter

cgtttcgcatgattgaacaagatggattgcacgcaggttctccggccgcttgggtggagaggcta  5330
gcaaagcgtactaacttgttctacctaacgtgcgtccaagaggccggcgaacccacctctccgat
1 M I E Q 5 D G L H A 10 G S P A A 15 W V E R L
NeoR/KanR

ttcggctatgactgggcacaacagacaatcggctgctctgatgccgccgtgttccggctgtcagc  5395
aagccgatactgacccgtgttgtctgttagccgacgagactacggcggcacaaggccgacagtcg
20 F G Y D W 25 A Q Q T I 30 G C S D A 35 A V F R L 40 S A
NeoR/KanR
```

gcaggggcgAccggttcttttgtcaagaccgacctgtccggtgccctgaatgaactgcaagacg 5460
cgtccccgcTggccaagaaaaacagttctggctggacaggccacgggacttacttgacgttctgc
45 50 55 60
Q G R P V L F V K T D L S G A L N E L Q D
NeoR/KanR

aggcagcgcggctatcgtggctggccacgacgggcgttccttgcgcagctgtgctcgacgttgtc 5525
tccgtcgcgccgatagcaccgaccggtgctgcccgcaaggaacgcgtcgacacgagctgcaacag
65 70 75 80
E A A R L S W L A T T G V P C A A V L D V V
NeoR/KanR

actgaagcgggaagggactggctgctattgggcgaagtgccggggcaggatctcctgtcatctca 5590
tgacttcgcccttccctgaccgacgataacccgcttcacggccccgtcctagaggacagtagagt
85 90 95 100 105
T E A G R D W L L L G E V P G Q D L L S S H
NeoR/KanR

ccttgctcctgccgagaaagtatccatcatggctgatgcaatgcggcggctgcatacgcttgatc 5655
ggaacgaggacggctctttcataggtagtaccgactacgttacgccgccgacgtatgcgaactag
110 115 120 125
L A P A E K V S I M A D A M R R L H T L D
NeoR/KanR

cggctacctgcccattcgaccaccaagcgaaacatcgcatcgagcgagcacgtactcggatggaa 5720
gccgatggacgggtaagctggtggttcgctttgtagcgtagctcgctcgtgcatgagcctacctt
130 135 140 145
P A T C P F D H Q A K H R I E R A R T R M E
NeoR/KanR

gccggtcttgtcgatcaggatgatctggacgaagagcatcaggggctcgcgccagccgaactgtt 5785
cggccagaacagctagtcctactagacctgcttctcgtagtccccgagcgcggtcggcttgacaa
150 155 160 165 170
A G L V D Q D D L D E E H Q G L A P A E L F
NeoR/KanR

cgccaggctcaaggcgagcatgcccgacggcgaggatctcgtcgtgacccatggcgatgcctgct 5850
gcggtccgagttccgctcgtacgggctgccgctcctagagcagcactgggtaccgctacggacga
175 180 185 190
A R L K A S M P D G E D L V V T H G D A C
NeoR/KanR

tgccgaatatcatggtggaaaatggccgcttttctggattcatcgactgtggccggctgggtgtg 5915
acggcttatagtaccaccttttaccggcgaaaagacctaagtagctgacaccggccgacccacac
195 200 205 210
L P N I M V E N G R F S G F I D C G R L G V
NeoR/KanR

gcggaccgctatcaggacatagcgttggctacccgtgatattgctgaagagcttggcggcgaatg 5980
cgcctggcgatagtcctgtatcgcaaccgatgggcactataacgacttctcgaaccgccgcttac
215 220 225 230 235
A D R Y Q D I A L A T R D I A E E L G G E W
NeoR/KanR

```
ggctgaccgcttcctcgtgctttacggtatcgccgctcccgattcgcagcgcatcgccttctatc    6045
ccgactggcgaaggagcacgaaatgccatagcggcgagggctaagcgtcgcgtagcggaagatag
         240                     245                     250                     255
 A    D    R    F    L    V    L    Y    G    I    A    A    P    D    S    Q    R    I    A    F    Y
NeoR/KanR

gccttcttgacgagttcttctgaAGGTAACCggcctcttcatcgggaatgcgcgcgaccttcagc    6110
cggaagaactgctcaagaagacTTCCATTGGccggagaagtagcccttacgcgcgctggaagtcg
         260              265
 R    L    L    D    E    F    F    *
NeoR/KanR

atcgccggcatgtcccccctggcggacgggaagtatccagctcgaccaagcttggcgagattttca    6175
tagcggccgtacaggggggaccgcctgcccttcataggtcgagctggttcgaaccgctctaaaagt

ggagctaaggaagctaaaatggagaaaaaaatcactggatataccaccgttgatatatcccaatg    6240
cctcgattccttcgattttacctctttttttagtgacctatatggtggcaactatatagggttac

gcatcgtaaagaacattttgaggcatttcagtcagttgctcaatgtacctataaccagaccgttc    6305
cgtagcatttcttgtaaaactccgtaaagtcagtcaacgagttacatggatattggtctggcaag

agctgcattaatgaatcggccaacgcgcggggagaggcggtttgcgtattgggcgctcttccgct    6370
tcgacgtaattacttagccggttgcgcgcccctctccgccaaacgcataacccgcgagaaggcga

tcctcgctcactgactcgctgcgctcggtcgttcggctgcggcgagcggtatcagctcactcaaa    6435
aggagcgagtgactgagcgacgcgagccagcaagccgacgccgctcgccatagtcgagtgagttt

ggcggtaatacggttatccacagaatcaggggataacgcaggaaagaacatgtgagcaaaaggcc    6500
ccgccattatgccaataggtgtcttagtcccctattgcgtcctttcttgtacactcgttttccgg

agcaaaaggccaggaaccgtaaaaaggccgcgttgctggcgtttttccataggctccgcccccct    6565
tcgttttccggtccttggcatttttccggcgcaacgaccgcaaaaaggtatccgaggcgggggga
ori

gacgagcatcacaaaaatcgacgctcaagtcagaggtggcgaaacccgacaggactataaagata    6630
ctgctcgtagtgtttttagctgcgagttcagtctccaccgctttgggctgtcctgatatttctat
ori

ccaggcgtttccccctggaagctccctcgtgcgctctcctgttccgaccctgccgcttaccggat    6695
ggtccgcaaagggggaccttcgagggagcacgcgagaggacaaggctgggacggcgaatggccta
ori

acctgtccgcctttctcccttcgggaagcgtggcgctttctcatagctcacgctgtaggtatctc    6760
tggacaggcggaaagagggaagcccttcgcaccgcgaaagagtatcgagtgcgacatccatagag
ori
```

```
agttcggtgtaggtcgttcgctccaagctgggctgtgtgcacgaaccccccgttcagcccgaccg   6825
tcaagccacatccagcaagcgaggttcgacccgacacacgtgcttggggggcaagtcgggctggc
ori

ctgcgccttatccggtaactatcgtcttgagtccaacccggtaagacacgacttatcgccactgg   6890
gacgcggaataggccattgatagcagaactcaggttgggccattctgtgctgaatagcggtgacc
ori

cagcagccactggtaacaggattagcagagcgaggtatgtaggcggtgctacagagttcttgaag   6955
gtcgtcggtgaccattgtcctaatcgtctcgctccatacatccgccacgatgtctcaagaacttc
ori

tggtggcctaactacggctacactagaagaacagtatttggtatctgcgctctgctgaagccagt   7020
accaccggattgatgccgatgtgatcttcttgtcataaaccatagacgcgagacgacttcggtca
ori

taccttcggaaaaagagttggtagctcttgatccggcaaacaaaccaccgctggtagcggtggtt   7085
atggaagcctttttctcaaccatcgagaactaggccgtttgtttggtggcgaccatcgccaccaa
ori

tttttgtttgcaagcagcagattacgcgcagaaaaaaaggatctcaagaagatcctttgatcttt   7150
aaaaacaaacgttcgtcgtctaatgcgcgtctttttttcctagagttcttctaggaaactagaaa
ori

tctacggggtctgacgctcagtggaacgaaaactcacgttaagggattttggtcatgagattatc   7215
agatgccccagactgcgagtcaccttgcttttgagtgcaattccctaaaaccagtactctaatag

aaaaaggatcttcacctagatccttttaaattaaaaatgaagttttaaatcaatctaaagtatat   7280
tttttcctagaagtggatctaggaaaatttaatttttacttcaaaatttagttagatttcatata

atgagtaaacttggtctgacagttaccaatgcttaatcagtgaggcacctatctcagcgatctgt   7345
tactcatttgaaccagactgtcaatggttacgaattagtcactccgtggatagagtcgctagaca
285 280 275
* W H K I L S A G I E A I Q
AmpR

ctatttcgttcatccatagttgcctgactccccgtcgtgtagataactacgatacgggagggctt   7410
gataaagcaagtaggtatcaacggactgaggggcagcacatctattgatgctatgccctcccgaa
270 265 260 255
R N R E D M T A Q S G T T Y I V V I R S P K
AmpR

accatctggccccagtgctgcaatgataccgcgagacccacgctcaccggctccagatttatcag   7475
tggtagaccggggtcacgacgttactatggcgctctgggtgcgagtggccgaggtctaaatagtc
250 245 240 235 230
G D P G L A A I I G R S G R E G A G S K D A
AmpR
```

caataaaccagccagccggaagggccgagcgcagaagtggtcctgcaactttatccgcctccatc 7540
gttatttggtcggtcggccttcccggctcgcgtcttcaccaggacgttgaaataggcggaggtag
225 220 215 210
I F W G A P L A S R L L P G A V K D A E M
AmpR

cagtctattaattgttgccgggaagctagagtaagtagttcgccagttaatagtttgcgcaacgt 7605
gtcagataattaacaacggcccttcgatctcattcatcaagcggtcaattatcaaacgcgttgca
205 200 195 190
W D I L Q Q R S A L T L L E G T L L K R L T
AmpR

tgttgccattgctacaggcatcgtggtgtcacgctcgtcgtttggtatggcttcattcagctccg 7670
acaacggtaacgatgtccgtagcaccacagtgcgagcagcaaaccataccgaagtaagtcgaggc
185 180 175 170 165
T A M A V P M T T D R E D N P I A E N L E P
AmpR

gttcccaacgatcaaggcgagttacatgatcccccatgttgtgcaaaaaagcggttagctccttc 7735
caagggttgctagttccgctcaatgtactagggggtacaacacgttttttcgccaatcgaggaag
160 155 150 145
E W R D L R T V H D G M N H L F A T L E K
AmpR

ggtcctccgatcgttgtcagaagtaagttggccgcagtgttatcactcatggttatggcagcact 7800
ccaggaggctagcaacagtcttcattcaaccggcgtcacaatagtgagtaccaataccgtcgtga
140 135 130 125
P G G I T T L L L N A A T N D S M T I A A S
AmpR

gcataattctcttactgtcatgccatccgtaagatgcttttctgtgactggtgagtactcaacca 7865
cgtattaagagaatgacagtacggtaggcattctacgaaaagacactgaccactcatgagttggt
120 115 110 105 100
C L E R V T M G D T L H K E T V P S Y E V L
AmpR

agtcattctgagaatagtgtatgcggcgaccgagttgctcttgcccggcgtcaacacgggataat 7930
tcagtaagactcttatcacatacgccgctggctcaacgagaacgggccgcagttgtgccctatta
95 90 85 80
D N Q S Y H I R R G L Q E Q G A D V R S L
AmpR

accgcgccacatagcagaactttaaaagtgctcatcattggaaaacgttcttcggggcgaaaact 7995
tggcgcggtgtatcgtcttgaaattttcacgagtagtaaccttttgcaagaagccccgcttttga
75 70 65 60
V A G C L L V K F T S M M P F R E E P R F S
AmpR

```
ctcaaggatcttaccgctgttgagatccagttcgatgtaacccactcgtgcacccaactgatctt    8060
gagttcctagaatggcgacaactctaggtcaagctacattgggtgagcacgtgggttgactagaa
   55                   50                   45                   40                   35
E  L  I  K  G  S  N  L  D  L  E  I  Y  G  V  R  A  G  L  Q  D  E
< AmpR

cagcatcttttactttcaccagcgtttctgggtgagcaaaaacaggaaggcaaaatgccgcaaaa    8125
gtcgtagaaaatgaaagtggtcgcaaagacccactcgtttttgtccttccgttttacggcgtttt
            30                   25                   20                   15
A  D  K  V  K  V  L  T  E  P  H  A  F  V  P  L  C  F  A  A  F
< AmpR          signal sequence

aagggaataagggcgacacggaaatgttgaatactcatactcttcctttttcaatattattgaag    8190
ttcccttattcccgctgtgcctttacaacttatgagtatgagaaggaaaaagttataataacttc
      10                   5                   1
F  P  I  L  A  V  R  F  H  Q  I  S  M
< signal sequence / AmpR        < AmpR promoter

catttatcagggttattgtctcatgagcggatacatatttgaatgtatttagaaaaataaacaaa    8255
gtaaatagtcccaataacagagtactcgcctatgtataaacttacataaatctttttatttgttt
< AmpR promoter

taggggttccgcgcacatttccccgaaaagtgccacctgacgtctaagaaaccattattatcatg    8320
atccccaaggcgcgtgtaaaggggcttttcacggtggactgcagattctttggtaataatagtac
< AmpR promoter

acattaacctataaaaataggcgtatcacgaggccctttcgtc    3′
                                          ••• 8363
tgtaattggatatttttatccgcatagtgctccgggaaagcag    5′
```

	Feature	Location	Size	Direction	Type
✓	**CMV enhancer**	93 .. 472	380 bp	⊢⊣	enhancer
	/note = human cytomegalovirus immediate early enhancer				
✓	**CMV promoter**	473 .. 676	204 bp	→	promoter
	/note = human cytomegalovirus (CMV) immediate early promoter				
✓	**TagSeq**	710 .. 740	31 bp	⊢⊣	misc_feature
✓	**PCAT2_1**	741 .. 1315	575 bp	→	ncRNA
	/ncRNA_class = lncRNA				
✓	**CMV enhancer**	1329 .. 1708	380 bp	⊢⊣	enhancer
	/note = human cytomegalovirus immediate early enhancer				
✓	**CMV promoter**	1709 .. 1912	204 bp	→	promoter
	/note = human cytomegalovirus (CMV) immediate early promoter				
✓	**TagSeq**	1914 .. 1944	31 bp	⊢⊣	misc_feature
✓	**PCAT2_2**	1945 .. 2519	575 bp	→	ncRNA
	/ncRNA_class = lncRNA				
✓	**CMV enhancer**	2533 .. 2912	380 bp	⊢⊣	enhancer
	/note = human cytomegalovirus immediate early enhancer				
✓	**CMV promoter**	2913 .. 3116	204 bp	→	promoter
	/note = human cytomegalovirus (CMV) immediate early promoter				
✓	**TagSeq**	3118 .. 3148	31 bp	⊢⊣	misc_feature
✓	**PCAT2_3**	3149 .. 3723	575 bp	→	ncRNA
	/ncRNA_class = lncRNA				
✓	**RSV promoter**	4946 .. 5208	263 bp	→	promoter
	/note = Rous sarcoma virus enhancer/promoter				
✓	**NeoR/KanR**	5274 .. 6068	795 bp	→	CDS
	/gene = *aph(3')-II* (or *nptII*)				
	/product = aminoglycoside phosphotransferase from Tn5				
	/note = confers resistance to neomycin, kanamycin, and G418 (Geneticin®)				
	/translation = MIEQDGLHAGSPAAWVERLFGYDWAQQTIGCSDAAVFRLSAQGRPVLFVKTDLSGALNELQDEAARLSWLATTGVPCAAVLDVVTEAGRDWLLL GEVPGQDLLSSHLAPAEKVSIMADAMRRLHTLDPATCPFDHQAKHRIERARTRMEAGLVDQDDLDEEHQGLAPAELFARLKASMPDGEDLVVTH GDACLPNIMVENGRFSGFIDCGRLGVADRYQDIALATRDIAEELGGEWADRFLVLYGIAAPDSQRIAFYRLLDEFF*				
	264 amino acids = 29.0 kDa				
✓	**ori**	6544 .. 7132	589 bp	←	rep_origin
	/direction = LEFT				
	/note = high-copy-number ColE1/pMB1/pBR322/pUC origin of replication				
✓	**AmpR**	7303 .. 8163	861 bp	←	CDS
	▸ 2 segments				
	/gene = *bla*				
	/product = β-lactamase				
	/note = confers resistance to ampicillin, carbenicillin, and related antibiotics				
	/translation = MSIQHFRVALIPFFAAFCLPVFA,HPETLVKVKDAEDQLGARVGYIELDLNSGKILESFRPEERFPMMSTFKVLLCGAVLSRVDAGQEQLGRRIHYSQ NDLVEYSPVTEKHLTDGMTVRELCSAAITMSDNTAANLLLTTIGGPKELTAFLHNMGDHVTRLDRWEPELNEAIPNDERDTTMPVAMATTLRKLLT GELLTLASRQQLIDWMEADKVAGPLLRSALPAGWFIADKSGAGERGSRGIIAALGPDGKPSRIVVIYTTGSQATMDERNRQIAEIGASLIKHW*				
	286 amino acids = 31.5 kDa				

	Feature	Location	Size			Type
✓	**AmpR promoter**	8164 .. 8268	105 bp		←	promoter
	/gene = *bla*					

REFERENCES AND NOTES

1. S. Negrini, V. G. Gorgoulis, T. D. Halazonetis, Genomic instability—An evolving hallmark of cancer. *Nat. Rev. Mol. Cell Biol.* **11**, 220–228 (2010).

2. Y. Yao, W. Dai, Genomic instability and cancer. *J. Carcinog. Mutagen.* **5,** 1000165 (2014).

3. N. Lacoste, A. Woolfe, H. Tachiwana, A. V. Garea, T. Barth, S. Cantaloube, H. Kurumizaka, A. Imhof, G. Almouzni, Mislocalization of the centromeric histone variant CenH3/CENP-A in human cells depends on the chaperone DAXX. *Mol. Cell* **53**, 631–644 (2014).

4. S. R. Zabaronick, J. K. Tyler, The histone chaperone anti-silencing function 1 is a global regulator of transcription independent of passage through S phase. *Mol. Cell. Biol.* **25**, 652–660 (2005).

5. N. A. Pchelintsev, T. McBryan, T. S. Rai, J. van Tuyn, D. Ray-Gallet, G. Almouzni, P. D. Adams, Placing the HIRA histone chaperone complex in the chromatin landscape. *Cell Rep.* **3**, 1012–1019 (2013).

6. T. S. Rai, J. J. Cole, D. M. Nelson, D. Dikovskaya, W. J. Faller, M. G. Vizioli, R. N. Hewitt, O. Anannya, T. McBryan, I. Manoharan, J. Van Tuyn, N. Morrice, N. A. Pchelintsev, A. Ivanov, C. Brock, M. E. Drotar, C. Nixon, W. Clark, O. J. Sansom, K. I. Anderson, A. King, K. Blyth, P. D. Adams, HIRA orchestrates a dynamic chromatin landscape in senescence and is required for suppression of neoplasia. *Genes Dev.* **28**, 2712–2725 (2014).

7. S. Henikoff, M. M. Smith, Histone variants and epigenetics. *Cold Spring Harb. Perspect. Biol.* **7**, a019364 (2015).

8. S. G. Franklin, A. Zweidler, Non-allelic variants of histones 2a, 2b and 3 in mammals. *Nature* **266**, 273–275 (1977).

9. M. J. Saunders, E. Yeh, M. Grunstein, K. Bloom, Nucleosome depletion alters the chromatin structure of *Saccharomyces cerevisiae* centromeres. *Mol. Cell. Biol.* **10**, 5721–5727 (1990).

10. G. Hartley, R. J. O’neill, Centromere repeats: Hidden gems of the genome. *Genes* **10**, 223 (2019).

11. H. F. Willard, Chromosome-specific organization of human alpha satellite DNA. *Am. J. Hum. Genet.* **37**, 524–532 (1985).

12. S. Pentakota, K. Zhou, C. Smith, S. Maffini, A. Petrovic, G. P. Morgan, J. R. Weir, I. R. Vetter, A. Musacchio, K. Luger, Decoding the centromeric nucleosome through CENP-N. *eLife* **6**, e33442 (2017).

13. D. R. Foltz, L. E. T. Jansen, A. O. Bailey, J. R. Yates, E. A. Bassett, S. Wood, B. E. Black, D. W. Cleveland, Centromere-specific assembly of CENP-a nucleosomes is mediated by HJURP. *Cell* **137**, 472–484 (2009).

14. M. Shuaib, K. Ouararhni, S. Dimitrov, A. Hamiche, HJURP binds CENP-A via a highly conserved N-terminal domain and mediates its deposition at centromeres. *Proc. Natl. Acad. Sci. U.S.A.* **107**, 1349–1354 (2010).

15. L. Andronov, K. Ouararhni, I. Stoll, B. P. Klaholz, A. Hamiche, CENP-A nucleosome clusters form rosette-like structures around HJURP during G1. *Nat. Commun.* **10**, 4436 (2019).

16. E. M. Dunleavy, D. Roche, H. Tagami, N. Lacoste, D. Ray-Gallet, Y. Nakamura, Y. Daigo, Y. Nakatani, G. Almouzni-Pettinotti, HJURP is a cell-cycle-dependent maintenance and deposition factor of CENP-A at centromeres. *Cell* **137**, 485–497 (2009).

17. H. Zhao, D. Winogradoff, M. Bui, Y. Dalal, G. A. Papoian, Promiscuous histone mis-assembly is actively prevented by chaperones. *J. Am. Chem. Soc.* **138**, 13207–13218 (2016).

18. D. M. Carone, C. Zhang, L. E. Hall, C. Obergfell, B. R. Carone, M. J. O'Neill, R. J. O'Neill, Hypermorphic expression of centromeric retroelement-encoded small RNAs impairs CENP-A loading. *Chromosome Res.* **21**, 49–62 (2013).

19. J. D. Brown, S. E. Mitchell, R. J. O'Neill, Making a long story short: Noncoding RNAs and chromosome change. *Heredity* **108**, 42–49 (2012).

20. F. Ferri, H. Bouzinba-Segard, G. Velasco, F. Hubé, C. Francastel, Non-coding murine centromeric transcripts associate with and potentiate Aurora B kinase. *Nucleic Acids Res.* **37**, 5071–5080 (2009).

21. D. Quénet, Y. Dalal, A long non-coding RNA is required for targeting centromeric protein A to the human centromere. *eLife* **3**, e03254 (2014).

22. S. M. McNulty, L. L. Sullivan, B. A. Sullivan, Human centromeres produce chromosome-specific and array-specific alpha satellite transcripts that are complexed with CENP-A and CENP-C. *Dev. Cell* **42**, 226–240.e6 (2017).

23. S. Rošić, F. Köhler, S. Erhardt, Repetitive centromeric satellite RNA is essential for kinetochore formation and cell division. *J. Cell Biol.* **207**, 335–349 (2014).

24. G. O. M. Bobkov, N. Gilbert, P. Heun, Centromere transcription allows CENP-A to transit from chromatin association to stable incorporation. *J. Cell Biol.* **217**, 1957–1972 (2018).

25. A. W. Grenfell, R. Heald, M. Strzelecka, Mitotic noncoding RNA processing promotes kinetochore and spindle assembly in *Xenopus*. *J. Cell Biol.* **214**, 133–141 (2016).

26. V. Régnier, P. Vagnarelli, T. Fukagawa, T. Zerjal, E. Burns, D. Trouche, W. Earnshaw, W. Brown, CENP-A is required for accurate chromosome segregation and sustained kinetochore association of BubR1. *Mol. Cell. Biol.* **25**, 3967–3981 (2005).

27. R. K. Athwal, M. P. Walkiewicz, S. Baek, S. Fu, M. Bui, J. Camps, T. Ried, M.-H. Sung, Y. Dalal, CENP-A nucleosomes localize to transcription factor hotspots and subtelomeric sites in human cancer cells. *Epigenetics Chromatin* **8**, 1–23 (2015).

28. T. Tomonaga, K. Matsushita, S. Yamaguchi, T. Oohashi, H. Shimada, T. Ochiai, K. Yoda, F. Nomura, Overexpression and mistargeting of centromere protein-A in human primary colorectal cancer. *Cancer Res.* **63**, 3511–3516 (2003).

29. J. Nye, D. Sturgill, R. Athwal, Y. Dalal, HJURP antagonizes CENP-A mislocalization driven by the H3.3 chaperones HIRA and DAXX. *PLOS ONE* **13**, e0205948 (2018).

30. X. Sun, P. L. Clermont, W. Jiao, C. D. Helgason, P. W. Gout, Y. Wang, S. Qu, Elevated expression of the centromere protein-A(CENP-A)-encoding gene as a prognostic and predictive biomarker in human cancers. *Int. J. Cancer* **139**, 899–907 (2016).

31. R. L. Shrestha, G. S. Ahn, M. I. Staples, K. M. Sathyan, T. S. Karpova, D. R. Foltz, M. A. Basrai, Mislocalization of centromeric histone H3 variant CENP-A contributes to chromosomal instability (CIN) in human cells. *Oncotarget* **8**, 46781–46800 (2017).

32. G. S. Hewawasam, K. Dhatchinamoorthy, M. Mattingly, C. Seidel, J. L. Gerton, Chromatin assembly factor-1 (CAF-1) chaperone regulates Cse4 deposition into chromatin in budding yeast. *Nucleic Acids Res.* **46**, 4440–4455 (2018).

33. P. Heun, S. Erhardt, M. D. Blower, S. Weiss, A. D. Skora, G. H. Karpen, Mislocalization of the *Drosophila* centromere-specific histone CID promotes formation of functional ectopic kinetochores. *Dev. Cell* **10**, 303–315 (2006).

34. A. K. Saha, R. Contreras-Galindo, Y. S. Niknafs, M. Iyer, T. Qin, K. Padmanabhan, J. Siddiqui, M. Palande, C. Wang, B. Qian, E. Ward, T. Tang, S. A. Tomlins, S. D. Gitlin, M. A. Sartor, G. S. Omenn, A. M. Chinnaiyan, D. M. Markovitz, The role of the histone H3 variant CENPA in prostate cancer. *J. Biol. Chem.* **295**, 8537–8549 (2020).

35. W. Zhang, J. H. Mao, W. Zhu, A. K. Jain, K. Liu, J. B. Brown, G. H. Karpen, Centromere and kinetochore gene misexpression predicts cancer patient survival and response to radiotherapy and chemotherapy. *Nat. Commun.* **7**, 12619 (2016).

36. B. Stangeland, A. A. Mughal, Z. Grieg, C. J. Sandberg, M. Joel, S. Nygård, T. Meling, W. Murrell, E. O. Vik Mo, I. A. Langmoen, Combined expressional analysis, bioinformatics and targeted proteomics identify new potential therapeutic targets in glioblastoma stem cells. *Oncotarget* **6**, 26192–26215 (2015).

37. E. Tian, J. Sawyer, O. W. Stephens, R. Tytarenko, S. Thanendrarajan, C. Schinke, M. Zangari, F. van Rhee, F. E. Davies, B. A. Walker, G. J. Morgan, Translocations and jumping rearrangements at 8q24 result in over-expression of MYC and are key drivers of disease progression. *Blood* **128**, 115 (2016).

38. Y. Hu, J. Gaedcke, G. Emons, T. Beissbarth, M. Grade, P. Jo, M. Yeager, S. J. Chanock, H. Wolff, J. Camps, B. M. Ghadimi, T. Ried, Colorectal cancer susceptibility loci as predictive markers of rectal cancer prognosis after surgery. *Genes Chromosomes Cancer* **57**, 140–149 (2018).

39. R. Dalla-Favera, S. Martinotti, R. C. Gallo, J. Erikson, C. M. Croce, Translocation and rearrangements of the c-myc oncogene locus in human undifferentiated B-cell lymphomas. *Science* **219**, 963–967 (1983).

40. C. Wu, P. M. Bingham, K. J. Livak, R. Holmgren, S. C. Elgin, The chromatin structure of specific genes: I. Evidence for higher order domains of defined DNA sequence. *Cell* **16**, 797–806 (1979).

41. M. A. Bender, M. Bulger, J. Close, M. Groudine, β-Globin gene switching and DNase I sensitivity of the endogenous β-globin locus in mice do not require the locus control region. *Mol. Cell* **5**, 387–393 (2000).

42. K. Huppi, J. J. Pitt, B. M. Wahlberg, N. J. Caplen, The 8q24 gene desert: An oasis of non-coding transcriptional activity. *Front. Genet.* **3**, 69 (2012).

43. G. Arunkumar, A. K. Murugan, H. Prasanna Srinivasa Rao, S. Subbiah, R. Rajaraman, A. K. Munirajan, Long non-coding RNA CCAT1 is overexpressed in oral squamous cell carcinomas and predicts poor prognosis. *Biomed. Rep.* **6**, 455–462 (2017).

44. L. Huang, Y. Wang, J. Chen, Y. Wang, Y. Zhao, Y. Wang, Y. Ma, X. Chen, W. Liu, Z. Li, L. Zhao, B. Shan, X. Dong, D. Li, S. Shao, Y. Song, Q. Zhan, X. Liu, Long noncoding RNA PCAT1, a novel serum-based biomarker, enhances cell growth by sponging miR-326 in oesophageal squamous cell carcinoma. *Cell Death Dis.* **10**, 513 (2019).

45. J.-F. Xiang, Q.-F. Yin, T. Chen, Y. Zhang, X.-O. Zhang, Z. Wu, S. Zhang, H.-B. Wang, J. Ge, X. Lu, L. Yang, L.-L. Chen, Human colorectal cancer-specific *CCAT1-L* lncRNA regulates long-range chromatin interactions at the *MYC* locus. *Cell Res.* **24**, 513–531 (2014).

46. H. Handa, K. Honma, T. Oda, N. Kobayashi, Y. Kuroda, K. Kimura-Masuda, S. Watanabe, R. Ishihara, Y. Murakami, Y. Masuda, K. I. Tahara, H. Takei, T. Kasamatsu, T. Saitoh, H. Murakami, Long noncoding RNA PVT1 is regulated by bromodomain protein BRD4 in multiple myeloma and

is associated with disease progression. *Int. J. Mol. Sci.* **21**, 7121 (2020). https://doi.org/10.3390/ijms21197121.

47. J. G. DeLuca, A. Musacchio, Structural organization of the kinetochore-microtubule interface. *Curr. Opin. Cell Biol.* **24**, 48–56 (2012).

48. K. H. Miga, S. Koren, A. Rhie, M. R. Vollger, A. Gershman, A. Bzikadze, S. Brooks, E. Howe, D. Porubsky, G. A. Logsdon, V. A. Schneider, T. Potapova, J. Wood, W. Chow, J. Armstrong, J. Fredrickson, E. Pak, K. Tigyi, M. Kremitzki, C. Markovic, V. Maduro, A. Dutra, G. G. Bouffard, A. M. Chang, N. F. Hansen, A. B. Wilfert, F. Thibaud-Nissen, A. D. Schmitt, J. M. Belton, S. Selvaraj, M. Y. Dennis, D. C. Soto, R. Sahasrabudhe, G. Kaya, J. Quick, N. J. Loman, N. Holmes, M. Loose, U. Surti, R. ana Risques, T. A. Graves Lindsay, R. Fulton, I. Hall, B. Paten, K. Howe, W. Timp, A. Young, J. C. Mullikin, P. A. Pevzner, J. L. Gerton, B. A. Sullivan, E. E. Eichler, A. M. Phillippy, Telomere-to-telomere assembly of a complete human X chromosome. *Nature* **585**, 79–84 (2020).

49. S. L. Anwar, W. Wulaningsih, U. Lehmann, Transposable elements in human cancer: Causes and consequences of deregulation. *Int. J. Mol. Sci.* **18**, 974 (2017).

50. D. Quénet, D. Sturgill, M. Olson, Y. Dalal, CENP-A associated lncRNAs influence chromosome segregation in human cells. bioRxiv 097956 [Preprint]. 3 January 2017. https://doi.org/10.1101/097956.

51. Y. Liu, H. Su, J. Zhang, Y. Liu, C. Feng, F. Han, Back-spliced RNA from retrotransposon binds to centromere and regulates centromeric chromatin loops in maize. *PLOS Biol.* **18**, e3000582 (2020).

52. G. Arunkumar, D. P. Melters, Centromeric transcription: A conserved swiss-army knife. *Genes* **11**, 911 (2020).

53. R. Saffery, H. Sumer, S. Hassan, L. H. Wong, J. M. Craig, K. Todokoro, M. Anderson, A. Stafford, K. H. A. Choo, Transcription within a functional human centromere. *Mol. Cell* **12**, 509–516 (2003).

54. F. Cerutti, R. Gamba, A. Mazzagatti, F. M. Piras, E. Cappelletti, E. Belloni, S. G. Nergadze, E. Raimondi, E. Giulotto, The major horse satellite DNA family is associated with centromere competence. *Mol. Cytogenet.* **9**, 35 (2016).

55. K. E. Gascoigne, K. Takeuchi, A. Suzuki, T. Hori, T. Fukagawa, I. M. Cheeseman, Induced ectopic kinetochore assembly bypasses the requirement for CENP-A nucleosomes. *Cell* **145**, 410–422 (2011).

56. Y. Arimura, K. Shirayama, N. Horikoshi, R. Fujita, H. Taguchi, W. Kagawa, T. Fukagawa, G. Almouzni, H. Kurumizaka, Crystal structure and stable property of the cancer-associated heterotypic nucleosome containing CENP-A and H3.3. *Sci. Rep.* **4**, 7115 (2014).

57. M. Pitman, Y. Dalal, G. A. Papoian, Minimal cylinder analysis reveals the mechanical properties of oncogenic nucleosomes. *Biophys. J.* **118**, 2309–2318 (2020).

58. N. Gil, I. Ulitsky, Regulation of gene expression by cis-acting long non-coding RNAs. *Nat. Rev. Genet.* **21**, 102–117 (2020).

59. R. E. Andersen, S. J. Hong, J. J. Lim, M. Cui, B. A. Harpur, E. Hwang, R. N. Delgado, A. D. Ramos, S. J. Liu, B. J. Blencowe, D. A. Lim, The long noncoding RNA Pnky is a trans-acting regulator of cortical development in vivo. *Dev. Cell* **49**, 632–642.e7 (2019).

60. R. C. Gehrau, D. S. D'Astolfo, C. I. Dumur, J. L. Bocco, N. P. Koritschoner, Nuclear expression of KLF6 tumor suppressor factor is highly associated with overexpression of ERBB2 oncoprotein in ductal breast carcinomas. *PLOS ONE* **5**, e8929 (2010).

61. M. T. Romanish, C. J. Cohen, D. L. Mager, Potential mechanisms of endogenous retroviral-mediated genomic instability in human cancer. *Semin. Cancer Biol.* **20**, 246–253 (2010).

62. M. Gonzalez, H. He, Q. Dong, S. Sun, F. Li, Ectopic centromere nucleation by CENP-A in fission yeast. *Genetics* **198**, 1433–1446 (2014).

63. E. M. Hildebrand, S. Biggins, Regulation of budding yeast CENP-A levels prevents misincorporation at promoter nucleosomes and transcriptional defects. *PLOS Genet.* **12**, e1005930 (2016).

64. L. Harewood, P. Fraser, The impact of chromosomal rearrangements on regulation of gene expression. *Hum. Mol. Genet.* **23**, R76–R82 (2014).

65. W. Feng, A. Chakraborty, Fragility extraordinaire: Unsolved mysteries of chromosome fragile sites, in *Advances in Experimental Medicine and Biology* (Springer, 2017), vol. 1042, pp. 489–526.

66. Y. Dalal, J. Nye, D. P. Melters, The Art of War: Harnessing the epigenome against cancer. *F1000Res.* **7**, 141 (2018).

67. S. A. Quinodoz, P. Bhat, N. Ollikainen, J. W. Jachowicz, A. K. Banerjee, P. Chovanec, M. R. Blanco, A. Chow, Y. Markaki, K. Plath, M. Guttman, RNA promotes the formation of spatial compartments in the nucleus. *Cell* **184**, 5775–5790.e30 (2021).

68. D. Ray-Gallet, M. D. Ricketts, Y. Sato, K. Gupta, E. Boyarchuk, T. Senda, R. Marmorstein, G. Almouzni, Functional activity of the H3.3 histone chaperone complex HIRA requires trimerization of the HIRA subunit. *Nat. Commun.* **9**, 3103 (2018).

69. J. I. Ohzeki, J. H. Bergmann, N. Kouprina, V. N. Noskov, M. Nakano, H. Kimura, W. C. Earnshaw, V. Larionov, H. Masumoto, Breaking the HAC barrier: Histone H3K9 acetyl/methyl balance regulates CENP-A assembly. *EMBO J.* **31**, 2391–2402 (2012).

70. L. A. Sanz, S. R. Hartono, Y. W. Lim, S. Steyaert, A. Rajpurkar, P. A. Ginno, X. Xu, F. Chédin, Prevalent, dynamic, and conserved R-loop structures associate with specific epigenomic signatures in mammals. *Mol. Cell* **63**, 167–178 (2016).

71. P. K. Mishra, A. Chakraborty, E. Yeh, W. Feng, K. S. Bloom, M. A. Basrai, R-loops at centromeric chromatin contribute to defects in kinetochore integrity and chromosomal instability in budding yeast. *Mol. Biol. Cell* **32**, 74–89 (2021).

72. L. Kabeche, H. D. Nguyen, R. Buisson, L. Zou, A mitosis-specific and R loop-driven ATR pathway promotes faithful chromosome segregation. *Science* **359**, 108–114 (2018).

73. M. Nakama, K. Kawakami, T. Kajitani, T. Urano, Y. Murakami, DNA-RNA hybrid formation mediates RNAi-directed heterochromatin formation. *Genes Cells* **17**, 218–233 (2012).

74. C. Racca, S. Britton, S. Hédouin, C. Francastel, P. Calsou, F. Larminat, BRCA1 prevents R-loop-associated centromeric instability. *Cell Death Dis.* **12**, 896 (2021).

75. L. F. Liu, J. C. Wang, Supercoiling of the DNA template during transcription. *Proc. Natl. Acad. Sci. U.S.A.* **84**, 7024–7027 (1987).

76. S. H. Kim, R. Vlijm, J. Van Der Torre, Y. Dalal, C. Dekker, CENP-A and H3 nucleosomes display a similar stability to force-mediated disassembly. *PLOS ONE* **11**, e0165078 (2016).

77. F. C. Lam, Y. W. Kong, Q. Huang, T.-L. Vu Han, A. D. Maffa, E. M. Kasper, M. B. Yaffe, BRD4 prevents the accumulation of R-loops and protects against transcription–replication collision events and DNA damage. *Nat. Commun.* **11**, 4083 (2020).

78. H. Masumoto, H. Masukata, Y. Muro, N. Nozaki, T. Okazaki, A human centromere antigen (CENP-B) interacts with a short specific sequence in alphoid DNA, a human centromeric satellite. *J. Cell Biol.* **109**, 1963–1973 (1989).

79. J. G. Henikoff, J. Thakur, S. Kasinathan, S. Henikoff, A unique chromatin complex occupies young α-satellite arrays of human centromeres. *Sci. Adv.* **1**, e1400234 (2015).

80. J. A. Smolka, L. A. Sanz, S. R. Hartono, F. Chédin, Recognition of rna by the S9.6 antibody creates pervasive artifacts when imaging RNA:DNA hybrids. *J. Cell Biol.* **220**, e202004079 (2021).
